# Biomarker-Guided Cardioprotection for Patients Treated With Anthracyclines: A Randomized Clinical Trial

**DOI:** 10.1001/jamanetworkopen.2025.46201

**Published:** 2025-12-03

**Authors:** Congying Xia, Amanda M. Smith, Bénédicte Lefebvre, Faizi A. Jamal, Saro H. Armenian, Daniel Koropeckyj-Cox, Liyong Zhang, Peter P. Liu, Daniel Landsburg, Amy S. Clark, Payal D. Shah, Rebecca A. Hubbard, Anran Huang, Sophia Golec, Maureen Hewitt, Nicholas S. Wilcox, Zhen Chen, Leah Rethy, Wonyoung Jung, Kyunga Ko, Vivek Narayan, Yehoda M. Martei, Ninian N. Lang, James L. Januzzi, G. Michael Felker, Bonnie Ky

**Affiliations:** 1 Division of Cardiology, Department of Medicine, Perelman School of Medicine, University of Pennsylvania, Philadelphia; 2 Thalheimer Center for Cardio-Oncology, Abramson Cancer Center, Perelman School of Medicine, University of Pennsylvania, Philadelphia; 3 Department of Population Sciences, City of Hope Comprehensive Cancer Center, Duarte, California; 4 University of Ottawa Heart Institute, University of Ottawa, Ottawa, Ontario, Canada; 5 Division of Hematology–Oncology, Hospital of the University of Pennsylvania, Philadelphia; 6 Department of Biostatistics, Brown University School of Public Health, Providence, Rhode Island; 7 Division of Cardiology, Tufts Medical Center, Boston, Massachusetts; 8 Abramson Cancer Center, Perelman School of Medicine, University of Pennsylvania, Philadelphia; 9 School of Cardiovascular and Metabolic Health, University of Glasgow, Glasgow, Scotland, United Kingdom; 10 Division of Cardiology, Massachusetts General Hospital, Harvard Medical School, Baim Institute for Clinical Research, Boston; 11 Duke Clinical Research Institute, Duke University School of Medicine, Durham, North Carolina; 12 Division of Cardiology, Duke University School of Medicine, Durham, North Carolina; 13 Department of Biostatistics, Epidemiology and Informatics, Perelman School of Medicine, University of Pennsylvania, Philadelphia

## Abstract

**Importance:**

There are gaps in the understanding of the clinical actionability of cardiovascular biomarkers for risk stratification during cardiotoxic chemotherapy.

**Objectives:**

To gain insights into an N-terminal pro–B-type natriuretic peptide (NT-proBNP)–guided approach for cardioprotection in patients with breast cancer or lymphoma treated with anthracyclines and quantify the feasibility, tolerability, and exploratory efficacy of an NT-proBNP–guided strategy compared with usual care.

**Design, Setting, and Participants:**

The NT-proBNP guide, a multicenter, randomized (stratified 1:1 by cancer type) clinical trial, included 100 participants with breast cancer or lymphoma initiating anthracyclines from March 18, 2021, to October 20, 2023, who were followed up for 12 months.

**Interventions:**

Study participants in the NT-proBNP–guided arm had biomarker concentrations measured prior to anthracycline initiation (baseline), at each cycle, and at 3, 6, 9, and 12 months. An elevated NT-proBNP concentration triggered the initiation or titration of neurohormonal therapy. Participants in the usual care arm received routine care without prospective monitoring of NT-proBNP concentrations.

**Main Outcomes and Measures:**

The primary outcomes were feasibility and safety of the NT-proBNP–guided approach. Feasibility was defined by recruitment, retention, and medication compliance rates. Safety outcomes were assessed according to the Common Terminology Criteria for Adverse Events, version 5.0, at each visit. Exploratory outcomes included differences in blinded, centrally quantified echocardiographic measures and NT-proBNP concentrations between the 2 arms. Analysis was performed on an intention-to-treat approach.

**Results:**

Across 100 participants (mean [SD] age, 52.2 [14.4] years; 86 women [86.0%]), 74 (74.0%) had breast cancer and 26 (26.0%) had lymphoma. At 12 months, the retention rate was 92.7% (89 of 96). In the NT-proBNP–guided arm, 27 participants had NT-proBNP elevations, with a median time from baseline to first elevation of 14 days (IQR, 0-76 days), and a median time between the first NT-proBNP elevation and neurohormonal therapy prescription of 1 day (IQR, 0.5-9 days). There were no significant differences in targeted adverse events between the NT-proBNP–guided (23 events) and usual care (16 events) arms (*P* = .13). At 3 months, left ventricular ejection fraction (LVEF) was modestly higher in the NT-proBNP–guided arm compared with usual care (mean difference, 2.0% [95% CI, 0.5%-3.5%]; *P* = .007). NT-proBNP concentrations increased in both arms over the study duration, but elevations were slightly attenuated in the NT-proBNP–guided arm.

**Conclusions and Relevance:**

This randomized clinical trial of an NT-proBNP–guided approach to neurohormonal therapy in patients with cancer treated with anthracyclines demonstrates the feasibility, safety, and potential modest, early improvement in LVEF of a biomarker-guided approach. These findings provide support for further study of an NT-proBNP–guided approach to cardioprotection for patients undergoing cancer treatment.

**Trial Registration:**

ClinicalTrials.gov Identifier: NCT04737265

## Key Points

**Question:**

What is the feasibility, safety, and exploratory efficacy of using the biomarker N-terminal pro–B-type natriuretic peptide (NT-proBNP) to guide cardioprotection during cardiotoxic cancer therapy?

**Findings:**

This randomized clinical trial with 100 participants demonstrated strong recruitment and retention rates and no significant differences in adverse events between an NT-proBNP–guided approach and usual care. At 3 months, left ventricular ejection fraction was modestly higher in the NT-proBNP–guided arm compared with usual care.

**Meaning:**

These findings provide the first proof of concept that a monitoring strategy using cardiac stress biomarkers in patients receiving anthracycline chemotherapy could be feasible and safe, and may potentially affect cardiac structure and function.

## Teaser

This randomized clinical trial examines the feasibility, tolerability, and exploratory efficacy of an N-terminal pro–B-type natriuretic peptide–guided approach for cardioprotection vs usual care in patients with breast cancer or lymphoma treated with anthracyclines.

## Introduction

Anthracyclines are a backbone therapy for many malignant neoplasms, but dose-dependent cardiotoxic events remain a concern. Complications, such as systolic dysfunction and heart failure (HF), are associated with treatment interruptions, adverse cardiovascular outcomes, and worse overall survival.^1^ An evidence-based, proactive approach to identify and treat patients at increased cardiovascular risk is needed.^2^

Cardio-oncology trials evaluating the efficacy of neurohormonal antagonists for cardioprotection have yielded mixed results, likely secondary to a “one size fits all” approach that includes low-risk populations.^3,4,5^ Moreover, neurohormonal therapy tolerability and concerns over acceptability of prophylactic cardiovascular medications during active cancer therapy limits the widespread adoption of a nonselective strategy.^6^ A risk-guided cardioprotection approach using biomarkers to identify patients at higher cardiovascular risk who may benefit most from neurohormonal therapy may improve acceptability and overall cardiovascular benefit, advancing a precision medicine approach.

In cardio-oncology, abnormal natriuretic peptides are associated with an increased risk of cardiac dysfunction, major adverse cardiac events, and cardiac death.^7,8,9^ Current societal statements suggest that circulating biomarkers such as N-terminal pro–B-type natriuretic peptide (NT-proBNP) are to be used in the risk stratification of patients treated with cardiotoxic cancer therapies,^10,11^ although the evidence to support this, and how exactly measures should guide therapy, are significant knowledge gaps. It remains unknown if strategies attempting to lower NT-proBNP concentrations during active cancer therapy are safe, feasible, and accepted by patients. Last, how a biomarker-guided strategy may affect measures of cardiac function and remodeling remains unknown.

This multicenter randomized clinical trial was therefore designed to evaluate the feasibility and safety of an NT-proBNP–guided strategy to neurohormonal therapy initiation and titration in patients treated with anthracycline-based chemotherapy, as compared with usual care. A secondary objective was to explore whether this strategy could attenuate declines in left ventricular ejection fraction (LVEF), adverse cardiac remodeling, and markers of stress.

## Methods

### Study Design

The pilot study of an NT-proBNP–guided strategy of cardioprotection (NT-proBNP guide) was a prospective, multicenter, open-label, randomized clinical trial that enrolled participants from March 18, 2021, to October 20, 2023, across multiple sites at the Abramson Cancer Center at the University of Pennsylvania Health System and the City of Hope Cancer Treatment Center (trial protocol in Supplement 1). Adults aged 18 years or older, with a diagnosis of breast cancer or lymphoma, and planning for anthracycline-based chemotherapy were eligible to participate. Participants who were receiving preexistent neurohormonal therapy were not excluded, as it would affect the generalizability of the results and effectively select a very low-risk population. Moreover, the goals of this study were for the clinical trial population to be indicative of usual care. The full eligibility criteria are provided in eTable 1 in Supplement 2. All participants provided written informed consent. The study was conducted in compliance with the Declaration of Helsinki^12^ and was approved by the institutional review boards at the University of Pennsylvania and City of Hope. The trial was registered at ClinicalTrials.gov (NCT04737265). This study followed the Consolidated Standards of Reporting Trials (CONSORT) reporting guideline. Demographic characteristics, including self-reported race and ethnicity (Black, Hispanic, White, other [Asian or self-identified mixed race], and unknown race or ethnicity), were collected per National Institutes of Health reporting requirements.

### Study Randomization and Procedures

After providing written informed consent, eligible participants were randomly allocated in a 1:1 ratio to receive either real-time NT-proBNP assessment with NT-proBNP–guided neurohormonal therapy (intervention) or usual care (no real-time NT-proBNP assessment). Randomization was stratified by cancer type and conducted in randomization blocks of 4. In both arms, a blood sample was collected at baseline (prior to the initiation of anthracycline chemotherapy), prior to each anthracycline cycle, and 3, 6, 9, and 12 months after baseline for post hoc, centralized NT-proBNP quantification. In participants randomized to the NT-proBNP–guided arm, NT-proBNP concentrations were measured clinically, in real time, at these same time points (eFigure 1 in Supplement 2). Transthoracic echocardiograms were performed and questionnaires were given at baseline and at 3, 6, 9, and 12 months.

### Study Intervention

Individuals randomized to the NT-proBNP–guided arm had NT-proBNP concentrations measured at the local clinical laboratory. Participants and their physicians were informed of their NT-proBNP results. Age-adjusted thresholds of NT-proBNP above the upper limit of normal (>125 pg/mL for participants <75 years and >450 pg/mL for participants ≥75 years [to convert to nanograms per liter, multiply by 1]) were used to trigger the initiation and/or titration of neurohormonal therapy with the aim of decreasing and normalizing NT-proBNP concentrations if possible. A predefined treatment algorithm for neurohormonal therapy initiation and titration was adapted from the American College of Cardiology/American Heart Association HF guidelines^13^ and used as a guide by the study cardiologists (B.L., F.A.J., and B.K.) with oncology clinician input (eFigure 2 in Supplement 2), although modifications based on individual participant clinical characteristics and preferences and physician discretion were allowed as per the trial protocol (Supplement 1). Neurohormonal therapy was actively managed and continued to the 12-month visit. Participants randomized to the usual care arm received routine clinical care and NT-proBNP concentrations were not measured in real time.

### Biomarker Assessment

Post hoc blinded quantitation of NT-proBNP concentrations for all participants was performed at the University of Ottawa Heart Institute Biomarker Discovery and Validation Core (eMethods 1 in Supplement 2). The assay measurement range is 10 to 35 000 pg/mL and the coefficient of variation ranges from 2.9% to 6.1%.

### Echocardiography Acquisition and Quantification

Transthoracic echocardiograms were performed by dedicated sonographers for all participants (eMethods 2 in Supplement 2). Quantitation of systolic function (LVEF), structure (left ventricular [LV] volumes, mass), diastolic function (E/e’), longitudinal and circumferential strain, and ventricular-arterial coupling (Ea/Ees, ratio of effective arterial elastance [Ea], and end-systolic elastance [Ees]) was performed at the Penn Center for Quantitative Echocardiography by an experienced, certified sonographer (Z.C.) blinded to all participant characteristics.^14^ The reproducibility of these measures has been well established, with an LVEF intraobserver coefficient of variation of 4.4%.^15^

### Questionnaires

Details of the questionnaires are elaborated in eMethods 3 in Supplement 2. Self-reported symptomatic adverse events (AEs) were recorded according to the patient-reported outcomes (PROs) version of the Common Terminology Criteria for Adverse Events (PRO-CTCAE) at baseline and 3, 6, 9, and 12 months.^16^ To assess medication compliance, the PRO Measurement Information System (PROMIS) Medication Adherence Scale (PMAS) was used for participants in the NT-proBNP–guided arm who were prescribed neurohormonal therapy.^17^

### Study Outcomes

Primary outcomes were the feasibility and safety of the overall NT-proBNP–guided approach. Feasibility was defined by recruitment, retention, and medication compliance rates. Safety outcomes were assessed according to the CTCAE, version 5.0,^18^ at each visit. Grade 3 or higher AEs and their relatedness to the study intervention were assessed at each study visit. Although all AEs were assessed, targeted AEs were a priori specified for reporting, based on their potential relationship to neurohormonal therapy administration or their representation of a cardiovascular event of interest. This approach was taken to ensure robust capture of AEs of interest.^19^ PRO-CTCAEs were also summarized to inform the frequency of self-reported AEs.

Secondary outcomes included centrally quantified NT-proBNP concentrations and echocardiographic measures (LVEF, LV end-systolic volume, LV end-diastolic volume, LV mass, E/e’, longitudinal and circumferential strain, and Ea/Ees). Clinical events included all-cause mortality, new or worsened clinical HF (urgent or new office or emergency department visit or hospitalization for HF), and cardiac dysfunction (≥10% absolute LVEF decline and to a value <50%).

### Sample Size

The sample size for this trial was a priori defined based on the AE incidence rate, as the primary outcome was safety and feasibility. We sought to enroll up to 115 participants; with an expected attrition rate of up to 10%, this would yield at least 50 participants in the NT-proBNP–guided arm. Assuming an incidence of 12% for targeted AEs,^20^ this would yield a half-width of 9.7% for the 95% CI for the AE incidence rate. Moreover, this sample size would have 80% power (2-sided α = .05) to detect a difference of 0.68 in NT-proBNP concentration on a log_2_ scale (assuming a mean [SD] of 0.2 [0.7], and 23 of 50 would have an elevated NT-proBNP concentration^7^), and a 7.9% absolute difference in LVEF between the NT-proBNP–guided arm and usual care (assuming an SD of 4.9%^15^).

### Statistical Analysis

Detailed statistical analyses are provided in eMethods 4 in Supplement 2. An intention-to-treat approach with all randomized participants analyzed according to randomization arm was used for analyses of the primary outcomes. Proportions of participants with grade 3 or higher AEs by randomization arm were compared using the χ^2^ test or the Fisher exact test, as appropriate. The distribution of individual PRO-CTCAE symptoms according to grade was graphically displayed, and PRO-CTCAE was adjusted for baseline scores using the baseline subtraction method.^21^

A modified intention-to-treat approach, defined as participants with a baseline measurement and at least 1 follow-up measurement, was used in the secondary outcomes analysis that evaluated the change in NT-proBNP concentrations and echocardiographic measures in the 2 arms over time. Trajectories of NT-proBNP concentrations over time were assessed graphically by plotting the estimated marginal mean changes from baseline separately for each arm. Mean changes were estimated using linear regression models via generalized estimating equations for repeated measures. To compare the mean change in each echocardiographic outcome over time across the 2 arms, models included a factorial treatment-by-visit interaction term.

A 2-sided *P* < .05 was considered statistically significant. All statistical analyses were conducted using R, version 4.4.0 (R Project for Statistical Computing).

## Results

### Participant Characteristics

**Figure 1.**
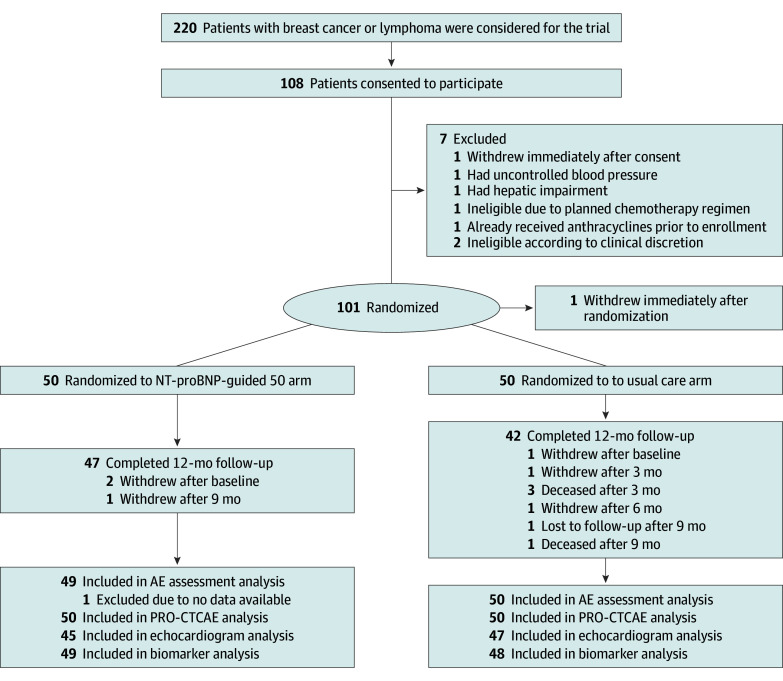
Flow Diagram Flow diagram of the N-terminal pro–B-type natriuretic peptide (NT-proBNP)-guide trial. AE indicates adverse event; PRO-CTCAE, patient-reported outcomes version of the Common Terminology Criteria for Adverse Events.

From March 2021 to October 2023, 220 patients with a diagnosis of breast cancer or lymphoma were approached, and 108 (49.1%) consented. Seven individuals were not randomized due to 1 of the following reasons: voluntary withdrawal, change in chemotherapy regimen, or uncontrolled blood pressure (Figure 1). Ultimately, 101 individuals were randomized but 1 withdrew immediately after randomization. As a result, 50 individuals were allocated to the NT-proBNP–guided arm and 50 individuals to the usual care arm. The mean (SD) age of participants was 52.2 (14.4) years and included 86 women (86.0%) and 14 men (14.0%); 10 Black participants (10.0%), 6 Hispanic participants (6.0%), 77 White participants (77.0%), 10 participants of other race or ethnicity (10.0%), and 3 participants with unknown race or ethnicity (3.0%); and 74 participants (74.0%) with a diagnosis of breast cancer (Table 1). Baseline demographic and clinical characteristics were largely comparable between the 2 arms, although a higher prevalence of hypertension was observed in the NT-proBNP–guided arm (21 [42.0%] vs 12 [24.0%]). Neurohormonal therapy at baseline was not an exclusion criterion to enhance the generalizability of our participants to reflect a usual care population. The median number of cycles of anthracycline treatment was 4 and the median equivalent dose was 240 mg/m^2^ in both arms.

**Table 1.** Baseline Demographic and Clinical Characteristics^a^

Characteristic	NT-proBNP–guided arm (n = 50)	Usual care arm (n = 50)
Demographics		
Age, mean (SD), y	54.5 (12.5)	50.0 (15.9)
Sex, No. (%)		
Female	42 (84.0)	44 (88.0)
Male	8 (16.0)	6 (12.0)
Race, No. (%)		
Black	5 (10.0)	5 (10.0)
White	38 (76.0)	39 (78.0)
Other^b^	6 (12.0)	4 (8.0)
Unknown	1 (2.0)	2 (4.0)
Ethnicity, No. (%)		
Hispanic or Latino	0	6 (12.0)
Not Hispanic or Latino	50 (100.0)	44 (88.0)
Clinical measures		
BMI, median (IQR)	28.1 (23.0-32.1)	27.2 (24.7-33.4)
Blood pressure, median (IQR), mm Hg		
Systolic	125 (112-137)	119 (111-130)
Diastolic	76 (69-83)	75 (68-80)
Heart rate, median (IQR), beats/min	75 (65-89)	83 (73-89)
eGFR, median (IQR), mL/min/1.73 m^2^	90 (76-103)	98 (84-109)
Cancer type, No. (%)		
Breast cancer	37 (74.0)	37 (74.0)
Lymphoma	13 (26.0)	13 (26.0)
Breast cancer, No./total No. (%)		
Breast cancer AJCC stages at diagnosis		
Stage I	6/37 (16.2)	3/37 (8.1)
Stage II	22/37 (59.5)	22/37 (59.5)
Stage III	9/37 (24.3)	12/37 (32.4)
Laterality		
Left	19/37 (51.4)	22/37 (59.5)
Right	17/37 (45.9)	14/37 (37.8)
Bilateral	1/37 (2.7)	1/37 (2.7)
Breast surgery, No./total No. (%)		
Partial mastectomy	14/37 (37.8)^c^	17/37 (45.9)
Mastectomy	21/37 (56.8)^c^	20/37 (54.1)
*ERBB2* status, negative	37/37 (100)	37/37 (100)
ER status, negative	17/37 (45.9)	20/37 (54.1)
PR status, negative	21/37 (56.8)	22/37 (59.5)
Lymphoma type, No./total No. (%)		
Hodgkin	5/13 (38.5)	6/13 (46.2)
Non-Hodgkin	8/13 (61.5)	7/13 (53.8)
Hodgkin lymphoma stage		
I	0	1/6 (16.7)
II	3/5 (60.0)	2/6 (33.3)
III	1/5 (20.0)	1/6 (16.7)
IV	1/5 (20.0)	2/6 (33.3)
Non-Hodgkin lymphoma stage		
I	2/8 (25.0)	1/7 (14.3)
IE	0	1/7 (14.3)
II	1/8 (12.5)	1/7 (14.3)
III	4/8 (50.0)	0
IIIE	1/8 (12.5)	1/7 (14.3)
IV	0	3/7 (42.9)
Cancer therapy		
Cumulative anthracycline dose, median (IQR), mg/m^2^	240 (240-240)	240 (234-240)
Pembrolizumab, No./total No. (%)	13/50 (26.0)^d^	20/50 (40.0)^d^
Chest radiotherapy (breast cancer), No./total No. (%)	30/37 (81.1)^d^	33/37 (89.2)
Chest radiotherapy site, No./total No. (%)		
Both	1/30 (3.3)	1/33 (3.0)
Left	17/30 (56.7)	21/33 (63.6)
Right	12/30 (40.0)	11/33 (33.3)
Cardiovascular risk factors, No. (%)		
Smoking status		
Current	0	3 (6.0)
Former	23 (46.0)	16 (32.0)
Never	27 (54.0)	31 (62.0)
History of hypertension	21 (42.0)	12 (24.0)
History of diabetes	4 (8.0)	2 (4.0)
History of hyperlipidemia	13 (26.0)	16 (32.0)
History of coronary disease	4 (8.0)	2 (4.0)
Cardiovascular medications, No. (%)		
β-Blocker	10 (20.0)	6 (12.0)
ACE inhibitor or angiotensin receptor blocker	14 (28.0)	5 (10.0)
Calcium-channel blocker	8 (16.0)	3 (6.0)
Diuretic	5 (10.0)	5 (10.0)
Statin	8 (16.0)	14 (28)

Abbreviations: ACE, angiotensin-converting enzyme; AJCC, American Joint Committee on Cancer; BMI, body mass index (calculated as weight in kilograms divided by height in meters squared); eGFR, estimated glomerular filtration rate; ER, estrogen receptor; NT-proBNP, N-terminal pro–B-type natriuretic peptide; PR, progesterone receptor.

^a^ Two participants in the NT-proBNP–guided arm received adjuvant trastuzumab emtansine after a neoadjuvant anthracycline-based regimen due to surgical pathology results being equivocal or positive for *ERBB2* (no other *ERBB2*-targeted therapies were administered and molecular status was classified as *ERBB2* negative); 2 participants received liposomal doxorubicin.

^b^ Included Asian or self-identified mixed race.

^c^ Two missing due to no available information.

^d^ One missing due to no available information.

### Study Retention, NT-proBNP Assessment, and Neurohormonal Therapy

**Figure 2.**
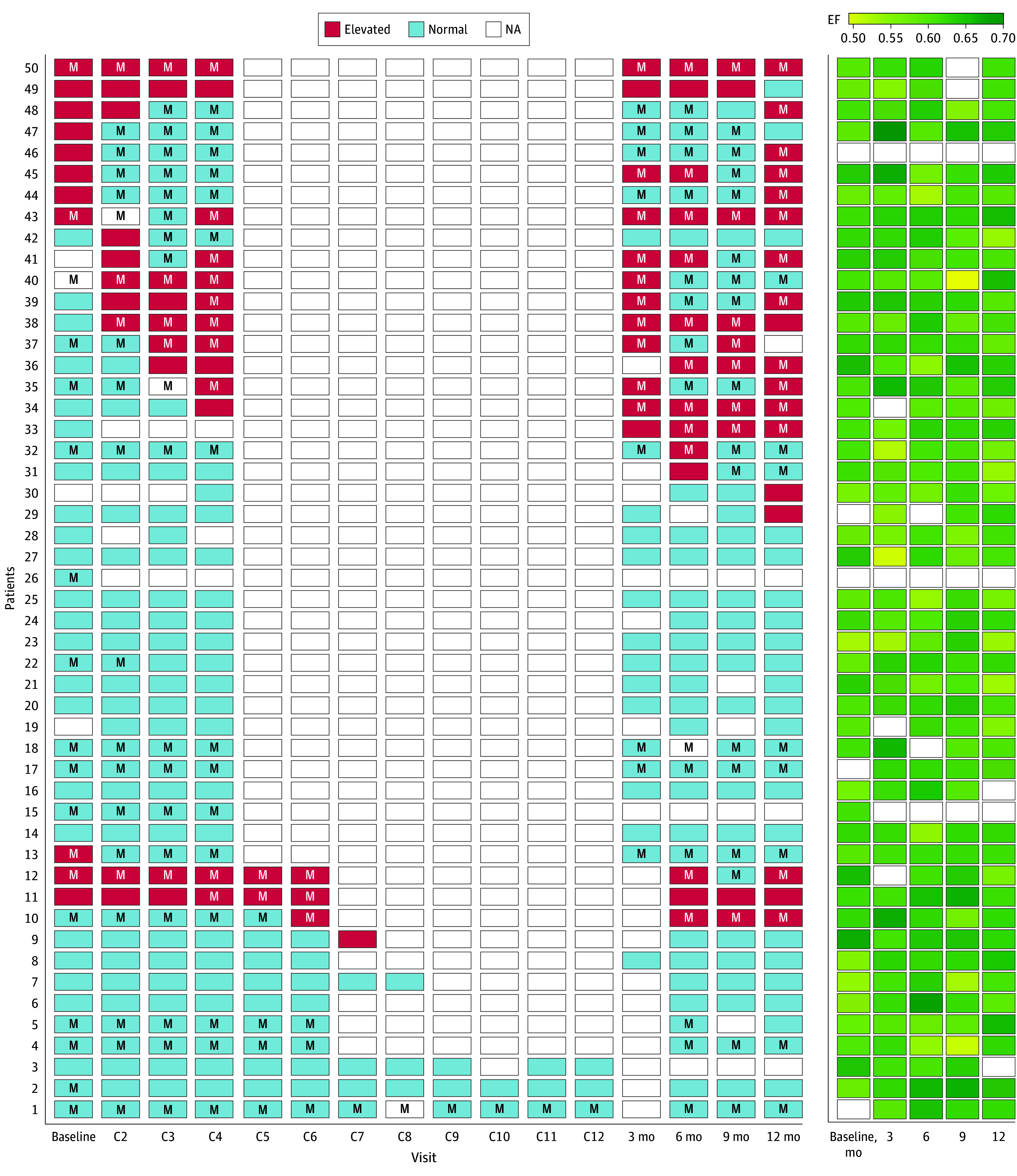
N-Terminal Pro–B-Type Natriuretic Peptide (NT-proBNP) Elevations and Neurohormonal Therapy Use NT-proBNP elevations, left ventricular ejection fraction, and neurohormonal therapy use are noted according to each participant and study visit in the NT-proBNP–guided arm. “M” denotes current neurohormonal therapy use. Red rectangles denote NT-proBNP concentration above the upper limit of normal. Each row presents an individual participant. “C” denotes anthracycline infusion cycles. There were 11 participants with baseline elevations of NT-proBNP concentrations. In total, 27 participants experienced at least 1 NT-proBNP concentration elevation. Of these, 4 did not have any initiation or titration of medications at any time point. EF indicates ejection fraction; NA, not available.

During the 12-month study duration, the overall retention rate was 92.7% (89 of 96): 94.0% (47 of 50) in the NT-proBNP–guided arm and 91.3% (42 of 46) in the usual care arm. In the NT-proBNP–guided arm, 27 participants had biomarker concentrations exceeding the upper limit of normal at least once during the study (Figure 2) with a median time from baseline to first biomarker elevation of 14 days (IQR, 0-76 days). Of these, 23 participants initiated or titrated neurohormonal therapy at a median time from baseline of 28 days (IQR, 5-53 days). The median time interval between the first NT-proBNP elevation and neurohormonal therapy prescription was 1 day (IQR, 0.5-9 days). Of the 4 study participants with NT-proBNP concentration elevations but who did not have any neurohormonal therapy initiated or titrated at any visit, 2 had their initial NT-proBNP concentration elevation at the 12-month visit, which was considered end of study. One participant consistently declined neurohormonal therapy, and 1 participant did not start neurohormonal therapy based on the clinical discretion of the study physician.

Changes and distribution of neurohormonal therapies over time are summarized in eFigure 3 in Supplement 2, and dosages across all visits are summarized in eTable 2 in Supplement 2. At 12 months, participants in the NT-proBNP–guided arm were more likely to be taking angiotensin-converting enzyme inhibitors or angiotensin receptor blockers (19 of 47 [40.4%] vs 2 of 42 [4.8%]; *P* < .001) and β-blockers (11 of 47 [23.4%] vs 3 of 42 [7.1%]; *P* = .04), compared with participants in the usual care arm. Overall, adherence to neurohormonal therapy was high, with a median PMAS total score for medication adherence of 31 (range, 17-33).

Not all NT-proBNP concentration elevations corresponded to blood pressure elevations, and not all blood pressure elevations corresponded to NT-proBNP concentration elevations. In the NT-proBNP–guided arm, among visits with an elevated NT-proBNP concentration, only 24.8% (25 of 101) were accompanied by a systolic blood pressure of 140 mm Hg or greater. Among visits with a systolic blood pressure of 140 mm Hg or greater, 39.1% (25 of 64) were accompanied by an NT-proBNP concentration elevation; 60.9% (39 of 64) were not.

### Safety Outcomes and PROs

A total of 39 study participants (23 in the NT-proBNP–guided arm and 16 in usual care; *P* = .13) had a targeted AE at any time point. Hypotension was observed in 4 participants in the NT-proBNP–guided arm and none in the usual care arm (Table 2), although median blood pressures were similar between the 2 arms. Only 1 participant’s hypotension was adjudicated as possibly related to the study intervention, while all other AEs were either unlikely related or unrelated. Qualitatively, there were no substantial differences in PRO-CTCAEs between the 2 arms over time (eFigure 4 in Supplement 2), although participants in the NT-proBNP–guided arm were less likely to report symptomatic palpitations throughout the study.

**Table 2.** Frequency of Grade 3 or Greater Targeted Adverse Events^a^

Adverse event	Total, No. (%) (N = 99^b^)	NT-proBNP–guided arm, No. (%) (n = 49)	Usual care arm, No. (%) (n = 50)	*P* value
Any targeted adverse event	39 (39.4)	23 (46.9)	16 (32.0)	.13
Acute kidney injury	4 (4.0)	2 (4.1)	2 (4.0)	>.99
Bradycardia	0	0	0	NA
Chest pain (cardiac)	1 (1.0)	1 (2.0)	0	.49
Cough	0	0	0	NA
Dehydration	3 (3.0)	2 (4.1)	1 (2.0)	.62
Dizziness	3 (3.0)	3 (6.1)	0	.12
Dyspnea	5 (5.1)	3 (6.1)	2 (4.0)	.68
Edema	0	0	0	NA
Fatigue	13 (13.1)	7 (14.3)	6 (12.0)	.74
Headache	2 (2.0)	2 (4.1)	0	.24
Heart failure	1 (1.0)	1 (2.0)	0	.49
Hypertension	18 (18.2)	11 (22.4)	7 (14.0)	.28
Hypotension	4 (4.0)	4 (8.2)	0	.06
Left ventricular systolic dysfunction	1 (1.0)	1 (2.0)	0	.49
Palpitations	0	0	0	NA
Presyncope	0	0	0	NA
Stroke	0	0	0	NA
Syncope	6 (6.1)	3 (6.1)	3 (6.0)	>.99

Abbreviations: NA, not applicable; NT-proBNP, N-terminal pro–B-type natriuretic peptide.

^a^ Rates are for participants who developed or worsened in severity at any time point throughout the study for grade 3 or greater adverse events. One participant who withdrew from the NT-proBNP–guided arm immediately after baseline had not completed any adverse event reporting data; this participant was classified as missing and was not included in the analysis.

^b^ There were 99 evaluable patients for assessment of adverse events.

### Secondary Outcomes of NT-proBNP and Cardiac Function and Remodeling Trajectories

**Figure 3.**
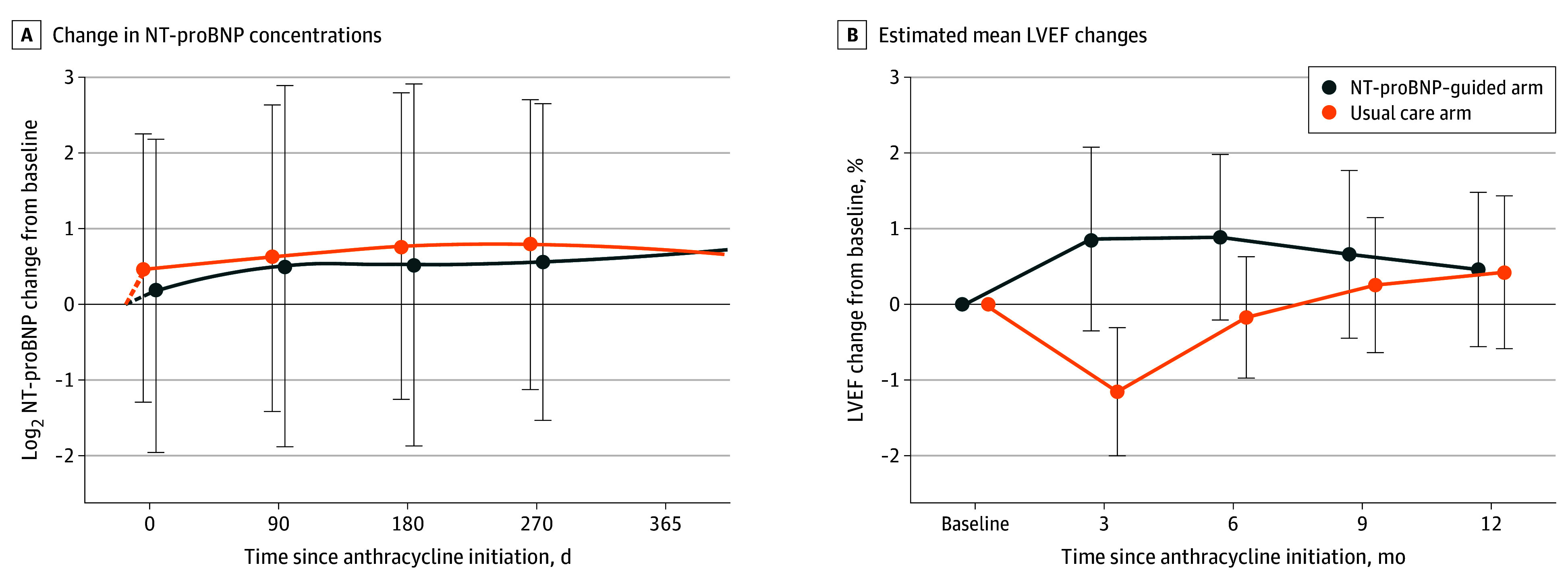
N-Terminal Pro–B-Type Natriuretic Peptide (NT-proBNP) Concentrations and Left Ventricular Ejection Fraction (LVEF) Trajectories By Treatment Arm A, Trajectories of change in NT-proBNP concentrations in the NT-proBNP–guided arm and usual care arm. Error bars are estimated means with 95% CIs at 2 weeks and 3, 6, 9, and 12 months after baseline, obtained from generalized estimating equations. NT-proBNP was log_2_ transformed, where each 1-unit increase indicates a doubling. B, Estimated mean changes in LVEF in the NT-proBNP–guided arm and usual care arm. Solid dots with error bars are estimated mean (95% CI) obtained from generalized estimating equations.

Baseline median NT-proBNP concentrations were 69 pg/mL (IQR, 32-121 pg/mL) in the NT-proBNP–guided arm compared with 54 pg/mL (IQR, 29-125 pg/mL) in the usual care arm. NT-proBNP concentrations increased in both arms over the duration of the study, but the magnitude of the increase tended to be lower in the biomarker-guided arm, particularly at earlier time points, compared with usual care (Figure 3A). At 2 weeks, the increase in NT-proBNP concentrations in the NT-proBNP–guided arm was lower than the increase in the usual care arm (estimated log_2_ difference, −0.42 [95% CI, −0.84 to −0.001]). However, the treatment-by-time interaction was not statistically significant (*P* = .16).

At baseline, the LVEF in the NT-proBNP–guided arm (59.1% [IQR, 56.6%-61.1%]) was not different from the LVEF among participants in the usual care arm (57.9% [IQR, 55.9%-61.1%]). At 3 months, LVEF was slightly greater in the NT-proBNP–guided arm compared with the usual care arm (mean difference, 2.0% [95% CI, 0.5%-3.5%]; *P* = .007). However, the difference between the 2 arms diminished over time, with a nonsignificant treatment-by-time interaction (*P* = .18) (Figure 3B). There were no significant differences in other secondary echocardiographic outcomes, although the directionality of the coefficients for E/e’, longitudinal strain, and circumferential strain tended toward an attenuation of dysfunction in the NT-proBNP–guided arm (eTable 3 in Supplement 2). Results remained similar after further adjustment for baseline use of neurohormonal therapy and treatment with pembrolizumab (eTable 4 in Supplement 2) and in a sensitivity analysis restricted to participants with NT-proBNP elevations in each arm (eTable 5 in Supplement 2).

### Clinical Events

Four deaths occurred, and all were in the usual care arm due to cancer progression. Two participants in the NT-proBNP–guided arm and 1 participant in the usual care arm experienced worsening or new-onset clinical HF. Two participants with prevalent HF at baseline were randomized to the NT-proBNP–guided arm and remained stable throughout the study. No participant had a decrease in LVEF of 10% to less than 50% while on study.

## Discussion

In this randomized clinical trial, an NT-proBNP–guided strategy to identify and treat patients at elevated risk with neurohormonal therapy was feasible, safe, and did not result in worsening of PROs among patients with breast cancer or lymphoma undergoing anthracycline chemotherapy. The study demonstrated strong recruitment and retention rates (the latter greater in the NT-proBNP–guided arm), high medication compliance, and no significant differences in AEs across the 2 arms. In the NT-proBNP–guided arm, 27 participants had NT-proBNP elevations with a median time to first elevation from baseline of 14 days (IQR, 0-76 days), and 23 of these participants initiated or uptitrated neurohormonal therapy (median time between first NT-proBNP elevation and neurohormonal therapy prescription, 1 day [IQR, 0.5-9 days]). During chemotherapy, NT-proBNP concentrations increased in both arms, although this increase was attenuated in the NT-proBNP–guided arm, particularly at early time points. At 3 months, LVEF was modestly higher (2.0% [95% CI, 0.5%-3.5%]) in the NT-proBNP–guided arm, although this effect was attenuated by 12 months. To our knowledge, the results from this pilot trial are the first to establish a proof of concept that a monitoring strategy for individuals undergoing cardiotoxic chemotherapy using a stress biomarker is safe, leads to more comprehensive cardioprotective treatment, and may have an effect on cardiac function.

The strategy of using NT-proBNP to guide neurohormonal therapy was safe and well tolerated, with no significant differences in targeted AEs compared with usual care. This finding is important; the effect of using natriuretic peptides to titrate neurohormonal therapy in a population with active cancer without any other evidence of overt cardiac dysfunction has never been tested, to our knowledge. Hypotension was observed in 4 participants in the NT-proBNP–guided arm; of these, only 1 was adjudicated as possibly related to the study intervention. We acknowledge that the intervention in this study and titration of neurohormonal therapy may have been less aggressive, as evidenced by the modest differences in NT-proBNP concentrations between the 2 arms, and the sample size of this pilot study may have limited the ability to detect less frequent AEs; in other studies, much higher rates of AEs (71.4%) have been reported, and dizziness and syncope more frequently reported in intervention groups that received combined neurohormonal therapy.^22^ Besides being safe, the guided approach did not result in worsening of PROs; prior cardio-oncology trials have not incorporated PRO-CTCAE in their outcomes assessment, despite their established value.^23^ Finally, the retention and compliance rates in this study were high (91.3%-94.0%), particularly compared with prior cardio-oncology trials with high withdrawal rates (17%) by 6 months,^24^ emphasizing the patient acceptability and feasibility of our approach. Our findings add to the existent literature as biomarker-guided strategies to identify patients at higher cardiovascular risk undergoing cardiotoxic chemotherapy have yielded conflicting results, including the most recent Cardiac CARE (High-Sensitivity Cardiac Troponin I–Guided Combination Angiotensin Receptor Blockade and Beta Blocker Therapy to Prevent Cardiac Toxicity in Cancer Patients Receiving Anthracycline Chemotherapy) trial, in which the troponin-triggered initiation of neurohormonal therapy did not demonstrate an improvement in LVEF in the short term,^22^ in contrast with prior studies.^25^ However, in support of NT-proBNP, participants with elevated NT-proBNP in the PREVENT-HF (Pharmacologic Reversal of Ventricular Remodeling in Childhood Cancer Survivors at Risk for Anthracycline-Related Heart Failure) study showed significantly improved cardiac remodeling at 2 years in the carvedilol arm compared with placebo.^26^

In our trial, a modestly higher LVEF at an early time point (3 months) was observed. These results may be of clinical relevance, as early LVEF declines are well established as an important finding as they are strongly associated with the risk of subsequent cardiac dysfunction.^15^ Preserving LVEF during the early stages of cancer therapy may help prevent unnecessary dose reduction or omission of anthracyclines, which could otherwise lead to worse survival.^27^ We also, however, acknowledge the attenuation of this LVEF difference by 12 months, which we hypothesize may have been secondary to the small sample size and the death of 4 patients in the usual care arm, which precluded echocardiographic assessment. It may also speak to the need for a longer study intervention, as we also noted 2 new NT-proBNP elevations at this late time point. We also cannot exclude the possibilities that our usual care arm was healthier, as has been reported in other clinical trials,^28^ or that our neurohormonal therapy titrations should have been more aggressive in reducing NT-proBNP concentrations, given the modest differences in concentrations between the 2 arms.

Although guidelines suggest the use of NT-proBNP in cardio-oncology,^11^ there is a lack of trial data to inform this recommendation. To our knowledge, this is the first prospective, multicenter randomized clinical trial to investigate the feasibility, safety, and efficacy of a strategy using NT-proBNP monitoring to initiate and titrate neurohormonal therapy to lower NT-proBNP concentrations in those without any other evidence of anthracycline-related cardiac dysfunction. We felt that demonstration of feasibility and safety was a necessary first step prior to embarking on a larger pivotal clinical trial, given that the clinical utility of an NT-proBNP–guided strategy to neurohormonal therapy initiation and titration has never been tested, and there are both patient and clinician concerns about additional noncancer medications, tolerability, and safety in a population with active cancer receiving chemotherapy.

### Strengths and Limitations

We note several strengths, including the use of a broad spectrum of neurohormonal therapies, compared with prior cardio-oncology trials that focused on 1 to 2 neurohormonal antagonists.^5,20,22,24^ We demonstrated strong feasibility with our enrollment and retention (91.3%-94.0%) and safety, with no serious AEs or PRO-CTCAEs attributed to NT-proBNP assessment or neurohormonal antagonists. Additional strengths included centralized, blinded quantification of NT-proBNP and echocardiographic parameters. Overall, our work provides the necessary scientific premise for additional research into a potential new paradigm in cardio-oncology care.

Limitations of this study exist. These include the relatively small sample size, which may have affected our ability to detect significant differences across various outcomes. Although we powered our study based on targeted AEs and did not detect significant differences across the 2 arms, we cannot exclude the possibility that a larger sample size may have led to statistically significant differences across these outcomes. Second, we included both patients with breast cancer and patients with lymphoma, resulting in some heterogeneity, although we incorporated a stratified randomization. Third, despite randomization, hypertension was more prevalent in the NT-proBNP–guided arm, although this may have biased our findings toward the null. Fourth, the study did not exclude those treated with neurohormonal therapy prior to study enrollment. This was intentional, as the goals were to recruit a trial population indicative of those cared for in everyday practice, enhancing the generalizability of the study findings. Fifth, a potential limitation may have been the open-label trial design; this design may have led to unconscious bias as physicians and patients were aware of study allocation. However, both measurements of NT-proBNP concentrations and echocardiographic outcomes were conducted by centralized core laboratories blinded to participant characteristics and randomization. Sixth, the study follow-up period was 12 months, and a longer study intervention and follow-up is important to assess the effect of this approach on late LVEF decreases.

## Conclusions

This randomized clinical trial found that, in patients treated with cardiotoxic anthracycline chemotherapy, an NT-proBNP–guided cardioprotection strategy was feasible and safe and resulted in a modest attenuation of NT-proBNP concentration elevations and early LVEF decreases compared with usual care. These findings provide support for further study of an NT-proBNP–guided approach to cardioprotection in patients undergoing cancer treatment.

## Notes

Supplement 1.
Trial Protocol and Statistical Analysis Plan

Supplement 2. **eMethods 1.** Biomarker Assessment **eMethods 2.** Echocardiography Acquisition and Quantification **eMethods 3.** Questionnaires **eMethods 4.** Statistical Analyses **eTable 1.** Eligibility Criteria **eTable 2.** Distribution of Total Daily Dose (mg) of Neurohormonal Antagonist Therapy for Participants in the NT-proBNP-Guided Arm Across All Study Timepoints **eTable 3.** Estimated Mean Change from Baseline in Echocardiographic Parameters Over Time Between the Two Arms **eTable 4.** Estimated Mean Change From Baseline in Echocardiographic Parameters Over Time Between the Two Arms, Adjusted for Baseline Use of Neurohormonal Therapy and Treatment With Pembrolizumab **eTable 5.** Estimated Mean Change From Baseline in Echocardiographic Parameters Over Time Between the Two Arms **eFigure 1.** Study Design **eFigure 2.** Recommended Algorithm for Neurohormonal Therapy in the NT-proBNP-Guided Study Arm **eFigure 3.** Neurohormonal Therapy Use by Treatment Arm Over Time **eFigure 4.** Patient-Reported Outcomes for Cardiac Symptoms by Treatment Arm
eReferences.

Supplement 3.
Data Sharing Statement
