# Supplementary material for: Biomarker-Guided Cardioprotection for Patients Treated With Anthracyclines: A Randomized Clinical Trial
Source: JAMA Netw Open. 2025 Dec 3;8(12):e2546201. doi: 10.1001/jamanetworkopen.2025.46201 (PMC12676363; doi:10.1001/jamanetworkopen.2025.46201)
Supplement: Supplement 1. — Trial Protocol and Statistical Analysis Plan [file jamanetwopen-e2546201-s001.pdf]

CLINICAL RESEARCH PROTOCOL

**INVESTIGATIONAL PRODUCT(S):**		1. Individually dosed Carvedilol (3.125mg - 25mg, twice daily orally)<br>2. Individually dosed Lisinopril (2.5mg-40mg, daily orally)/individually dosed Candesartan (4mg-32mg, daily orally)<br>3. Individually dosed Eplerenone (12.5mg daily orally)
**STUDY NUMBER(S):**	**IRB Number**	844092
	**UPCC Number**	25920
	**NCT Number**	NCT04737265
**PROTOCOL(S) TITLE:**		A Randomized, Open Label Pilot Trial of a Biomarker Guided Strategy of Cardioprotection in Patients with Lymphoma or Breast Cancer Treated with Anthracyclines
**IND NUMBER:**		IND exempt per OCR
**REGULATORY SPONSOR:**		Bonnie Ky, MD, MSCE<br>3400 Civic Center Blvd, 11-105 SCTR<br>Philadelphia, PA 19104
**FUNDING SPONSOR(S):**		National Institutes of Health/National Heart, Lung, and Blood Institute
**MEDICAL DIRECTOR**		Bonnie Ky, MD, MSCE
**ORIGINAL PROTOCOL DATE:**		10/22/2020
**VERSION NUMBER:**		6
**VERSION DATE:**		7/14/2023

1 Table of Contents

CLINICAL RESEARCH PROTOCOL 1

PRINCIPAL INVESTIGATOR SIGNATURE 5

1 Study Summary 7
1.1 Synopsis 7
1.2 Key Roles and Study Governance 9
1.3 Schema 9

2 Introduction and Rationale 10
2.1 Study Rationale 10
2.2 Background 10
2.2.1 Cardioprotective Strategies for Cancer Patients 10
2.2.2 Preliminary Data 11
2.2.3 Pharmacokinetics, Pharmacodynamics and Toxicology 14
2.2.4 Assessment for Potential Study Products Drug-Drug Interactions 18
2.2.5 Clinical Adverse Event Profile 19
2.2.6 Dosing Rationale 19
2.3 Risk/Benefit Assessment 19
2.3.1 Known Potential Risks 19
2.3.2 Known Potential Benefits 24
2.3.3 Assessment of Potential Risks and Benefits 24

3 Study Objectives and Endpoints 25

4 Study Plan 27
4.1 Study Design 27
4.2 Scientific Rationale for Study Design 27
4.3 Justification for Dose 28
4.4 End of Study Definition 28

5 Study Population 28
5.1 Inclusion Criteria 28
5.2 Exclusion Criteria 28
5.3 Lifestyle Considerations 29
5.4 Screen Failures 29
5.5 Strategies for Recruitment and Retention 30

6 Study Intervention 31
6.1 Study Intervention 31
6.1.1 Study Intervention Description 31
6.1.2 Dosing and Administration of Neurohormonal Antagonists 31
6.2 Preparation/Handling/Storage/Accountability 32
6.2.1 Acquisition and accountability 32
6.2.2 Formulation, Appearance, Packaging, and Labeling 32

6.2.3 Product Storage and Stability ... *32*
6.2.4 Preparation ... *32*
6.3 Measures to Minimize Bias: Randomization and Blinding ... 32
6.4 Study Intervention Compliance ... 33
6.5 Concomitant Therapy ... 33
6.6 Rescue Medicine ... 33
6.7 Study Intervention Discontinuation and Participant Discontinuation or Withdrawal ... 33
6.7.1 Discontinuation of Study Intervention ... *33*
6.7.2 Participant Discontinuation/Withdrawal from the Study ... *33*
6.7.3 Data Collection/Follow-up for Withdrawn Subjects ... *34*
6.8 Lost to Follow-Up ... 34
7 Study Assessment and Procedures ... 36
7.1 Study Procedures ... 37
7.1.1 Overview of Study Procedures ... *37*
7.1.2 Screening and Randomization ... *37*
7.1.3 Baseline Visit ... *38*
7.1.4 Intervention Arm: Neurohormonal antagonist initiation visit ... *38*
7.1.5 Intervention Arm: Neurohormonal antagonist titration visit ... *39*
7.1.6 Intervention Arm: Unscheduled Titration Visit ... *40*
7.1.7 Anthracycline Infusion Visit ... *41*
7.1.8 3-, 6-, 9-, and 12-Month Visits ... *41*
7.1.9 Off-Intervention Follow-Up ... *41*
7.1.10 Standard Care Arm ... *42*
7.2 Study Evaluations and Measurements ... 42
7.2.1 Clinical Covariates ... *42*
7.2.2 Vital Signs ... *43*
7.2.3 Laboratory Evaluations ... *43*
7.2.4 Pregnancy Testing ... *43*
7.2.5 Other Evaluations, Measures ... *43*
7.3 Safety and Other Assessments ... 45
7.4 Adverse Events and Serious Adverse Events ... 45
7.4.1 Definition of Adverse Events (AE) ... *45*
7.4.2 Definition of Serious Adverse Events (SAE) ... *46*
7.4.3 Classification of an Adverse Event ... *46*
7.4.4 Time Period and Frequency for Event Assessment and Follow-Up ... *47*
7.4.5 Adverse Event Recording ... *47*
7.4.6 Adverse Event Reporting ... *48*
7.4.7 Serious Adverse Event Reporting ... *48*
7.4.8 Reporting Events to Participants ... *49*
7.4.9 Events of Special Interest ... *49*
7.4.10 Reporting of Pregnancy ... *49*
7.5 Unanticipated Problems ... 50
7.5.1 Definition of Unanticipated Problems (UP) ... *50*

7.5.2 Unanticipated Problem Reporting ... *50*
7.5.3 Reporting Unanticipated Problems to Participants ... *51*

8 Statistical Considerations ... 51
8.1 Statistical Hypotheses ... 51
8.2 Sample Size Determination ... 51
8.3 Populations for Analyses ... 51
8.4 Statistical Analyses ... 52
8.4.1 General Approach ... *52*
8.4.2 Analysis of the Primary Efficacy Endpoint(s) ... *52*
8.4.3 Analysis of the Secondary Endpoint(s) ... *52*
8.4.4 Safety Analyses ... *53*
8.4.5 Baseline Descriptive Statistics ... *53*
8.4.6 Planned Interim Analyses ... *53*
8.4.7 Sub-Group Analyses ... *53*
8.4.8 Tabulation of Individual Participant Data ... *53*
8.4.9 Exploratory Analyses. ... *53*

9 Supporting Documentation and Operational Considerations ... 53
9.1 Regulatory, Ethical, and Study Oversight Considerations ... 53
9.1.1 Informed Consent Process ... *53*
9.1.2 Study Discontinuation and Closure ... *55*
9.1.3 Future Use of Stored Specimens and Data ... *55*
9.1.4 Monitoring ... *55*
9.1.5 Quality Assurance and Quality Control ... *56*
9.1.6 Data Handling and Record Keeping ... *56*
9.1.7 Protocol Deviations ... *57*
9.1.8 Publication and Data Sharing Policy ... *58*
9.1.9 Conflict of Interest Policy ... *58*
9.2 Protocol Amendment History ... 58

10 References ... 61

PRINCIPAL INVESTIGATOR SIGNATURE

STUDY SPONSOR:	National Heart, Lung, and Blood Institute
STUDY TITLE:	The Feasibility of a Biomarker Guided Strategy in Anthracycline Cardiotoxicity: A Randomized, Open-Label Pilot Trial of a Biomarker Guided Strategy for Cardioprotection in Patients with Lymphoma or Breast Cancer Treated with Anthracyclines (NT-proBNP Guide)
STUDY ID	pending
PROTOCOL VERSION	v6 7/17/2023

I have read the referenced protocol. I agree to conduct the study in accordance to this protocol, in compliance with the Declaration of Helsinki, Good Clinical Practices (GCP), and all applicable regulatory requirements and guidelines.

Principal Investigator Name	______________________	Signature	______________________
Affiliation:	______________________	Date	______________________

Abbreviations

AE	Adverse Event
ACE-I	Angiotensin-Converting Enzyme Inhibitor
ARB	Angiotensin II Receptor Blocker
BB	Beta-Blocker
CV	Cardiovascular
CTCAE	Common Terms and Criteria for Adverse Events
CTX	Cardiotoxicity
HF	Heart Failure
LVEF	Left Ventricular Ejection Fraction
CFR	Code of Federal Regulations
NT-proBNP	The N-terminal Pro-hormone of Brain Natriuretic Peptide
MP	Monitoring Plan
CONSORT	Consolidated Standards of Reporting Trials
CRF	Case Report Form
FDA	Food and Drug Administration
GCP	Good Clinical Practice
HIPAA	Health Insurance Portability and Accountability Act
IRB	Institutional Review Board
MOP	Manual of Procedures
NIH	National Institutes of Health
OHRP	Office for Human Research Protections
PI	Principal Investigator
QC	Quality Control
SAE	Serious Adverse Event
SoA	Schedule of Activities
SOP	Standard Operating Procedure
UP	Unanticipated Problem
US	United States

1 Study Summary

1.1 Synopsis

**Title:**	A Randomized, Open-Label Pilot Trial of Biomarker-Guided Cardioprotection in Patients with Lymphoma or Breast Cancer Treated with Anthracyclines
**Short Title:**	NTproBNP-Guide Pilot
**Study Description:**	We propose a randomized, open-label pilot trial of a biomarker-guided strategy using NT-proBNP to identify and treat patients with a high risk of cancer therapy-related cardiotoxicity. Patients will be enrolled and randomized prior to initiation of anthracycline-based therapy and followed for 12 months with blood samples, echocardiography, and patient reported outcomes surveys. The overall hypothesis is that a biomarker guided treatment strategy that initiates neurohormonal antagonists in breast cancer or lymphoma patients who have increases in NT-proBNP prior to, during, or after anthracyclines will be feasible, well-tolerated, and result in attenuation of cardiotoxicity, compared to standard care.
**Objectives:**	Primary Objective: To determine if a biomarker guided strategy using NT-proBNP to identify and treat high cardiovascular risk patients is feasible and well-tolerated in breast cancer or lymphoma patients treated with doxorubicin.<br>Secondary Objectives: To determine the change in NT-proBNP with neurohormonal antagonists in patients with an NT-proBNP>ULN in the biomarker guided arm, and to explore if a biomarker guided strategy, compared with standard care, results in decreased cardiotoxicity in breast cancer or lymphoma patients treated with doxorubicin.
**Primary Endpoint:**	• Rates of recruitment and retention<br>• Rates of adherence and compliance<br>• Maximum tolerated dosages of neurohormonal antagonists<br>• Rates of adverse events by CTCAEv5.0
**Secondary Endpoints:**	• Change in NT-proBNP following neurohormonal blockade in patients in the biomarker guided arm.<br>• Change in left ventricular ejection fraction (LVEF) over 12 months<br>• 12-month incidence of cardiotoxicity (defined as left ventricular ejection fraction decline ≥10% to less than 50%)<br>• Incidence of clinical heart failure (adjudicated by central committee)<br>• Frequency of treatment interruptions due to cardiotoxicity<br>• Change is self-reported quality of life<br>• Incidence of early discontinuation/dose reduction of anthracycline chemotherapy

**Exploratory Endpoints:**	• Changes in echocardiography derived functional measures (E/e', longitudinal and circumferential strain, VA coupling (Ea/Ees)) • Total dose of anthracycline chemotherapy delivered
**Study Population:**	Up to 115 adult patients diagnosed with stage I-III breast cancer or stage I-IV lymphoma treated with anthracyclines will be enrolled for up to 102 randomized.
**Phase:**	Pilot
**Enrolling Participants:**	Abramson Cancer Center, Philadelphia PA
**Description of Study Intervention:**	Patients will be randomized to either a biomarker guided strategy or standard care, stratified by cancer type and concurrent trastuzumab use. In the biomarker-guided arm, patients will have NT-proBNP levels drawn prior to anthracycline chemotherapy, at each anthracycline chemotherapy cycle and at all study visits (3, 6, 9 and 12 months). NT-proBNP > ULN will trigger the initiation of heart failure therapy with counseling from a cardiologist or other physician designated by the PI, based on a pre-specified algorithm. NTproBNP will not be monitored in the standard care arm. For both arms, echocardiograms and questionnaires will occur at baseline, 3, 6, 9, 12 months (from anthracycline chemotherapy initiation). Visits will coincide with chemotherapy cycles as much as possible; in breast cancer, chemotherapy cycles occur every 2 weeks for 4 months in doxorubicin without trastuzumab, and every 2-6 weeks for 12 months in doxorubicin with trastuzumab. In lymphoma, chemotherapy occurs every 3 to 4 weeks for 3 to 6 months.
**Study Duration:**	2 years
**Participant Duration:**	12 months

1.2 Key Roles and Study Governance

Study Principal Investigator
Bonnie Ky, MD, MSCE
University of Pennsylvania
3400 Civic Center Blvd, 11-105 SCTR<br>Philadelphia, PA 19104
267-977-3126
Bonnie.ky@pennmedicine.upenn.edu

1.3 Schema

Study Overview is shown in Figure 1 below.

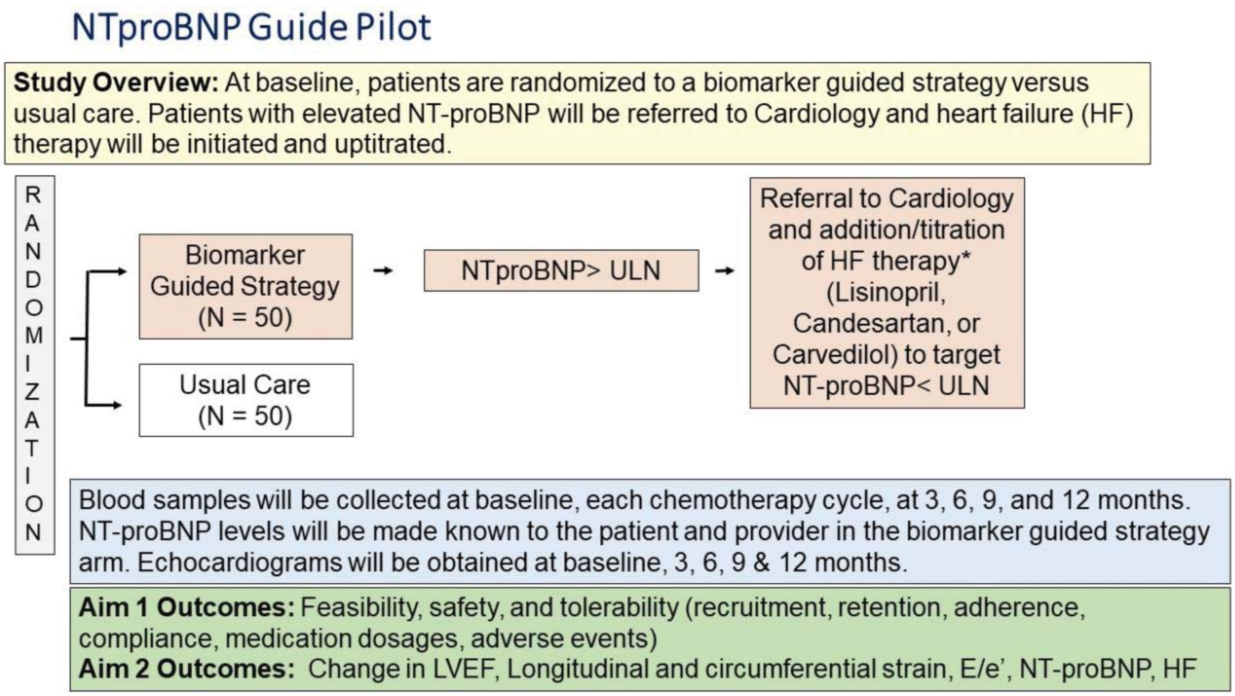

2 Introduction and Rationale

2.1 Study Rationale

Anthracyclines are used widely in the treatment of multiple cancers and have led to important oncologic survival gains in breast cancer and lymphoma. However, these agents carry a significant risk of cardiovascular (CV) morbidity and mortality. Doxorubicin-induced CV dysfunction, defined by declines in left ventricular ejection fraction (LVEF), occurs in 10-15% of patients at 240mg/m^2.[1] Cardiotoxic effects worsen with higher dosages, and when anthracyclines are used in combination with trastuzumab and/or chest radiation therapy. Doxorubicin and trastuzumab in combination result in LVEF declines in up to 33% and severe heart failure (HF) in 2-4%.[2] The development of LVEF declines and HF results in treatment interruptions, inferior oncologic outcomes, and worse overall survival.[2,3] In breast cancer, the late mortality risk of CV disease exceeds that of cancer.[4] In lymphoma, there is a nearly 3-fold increased risk of HF with anthracyclines compared to controls.[5–7] The NIH and professional societies have recognized the need to understand which cancer patients are at increased risk for cardiotoxicity (CTX) and to develop strategies to mitigate this risk (NIH PA 18-013).[8,9] Trials of prophylactic cardioprotection have suggested modest positive effects,[10–14] likely secondary to a "one size fits all" approach that includes those at low CTX risk. We hypothesize that widely available CV biomarkers can guide cardioprotection, advancing a personalized approach focused on the identification and treatment of those at increased CTX risk.

2.2 Background

Anthracyclines are used widely in breast cancer and as an essential strategy in lymphoma but can have potentially devastating effects on the cardiovascular (CV) system. Breast cancer affects every 1 in 8 women, with over 245,000 new cases each year.[15] In a population of over 3.5 million survivors, late CV mortality actually exceeds oncologic mortality.[4,16,17] There are over 82,000 new cases of lymphoma each year. Cardiotoxic therapies, such as anthracyclines still play a vital role in the current treatment era, particularly in these two cancers.[18,19] Doxorubicin-induced LVEF declines occur in approximately 10-15% of patients. Asymptomatic LVEF declines result in Stage B heart failure (HF), cancer therapy interruptions, and worse outcomes.[20–22] Overt HF secondary to anthracyclines carries a particularly poor prognosis. Trastuzumab, a humanized monoclonal antibody that disrupts ErbB2 (HER2/neu) signaling, has revolutionized the care of HER2+ cancer, but results in worse cardiotoxicity (CTX) when administered with anthracyclines.[13,23] Chest radiation therapy also worsens anthracycline CTX. In this study, we include both breast cancer and lymphoma patients given: 1) preliminary data suggesting significant associations between anthracyclines and CTX in both populations, 2) biologic plausibility and epidemiologic data to support a similar CTX effect in both cancer groups with anthracyclines, and 3) our desire to ultimately conduct a Phase III trial comprised of both populations. Notably, the ongoing PREVENT trial (NCT01988571) of statin cardioprotection in cancer patients receiving anthracyclines consists of patients with breast cancer or lymphoma.

2.2.1 Cardioprotective Strategies for Cancer Patients

Multiple small studies in cardio-oncology evaluating ACE-Is, ARBs and beta blockers have yielded mixed results with a very mild overall attenuation of LVEF declines in "all comers"

treated with anthracyclines or trastuzumab.[10–14] These RCTs have primarily consisted of low CV risk populations, with only ~ 4% with hypertension in contrast to a typical prevalence of 30% encountered in everyday clinical care. We hypothesize this "one size fits all" strategy is the reason for the lack of substantial benefit of cardioprotective strategies.[11,12] This strategy also results in the treatment of a population who may not derive benefit and who may not tolerate the medications. Prophylactic use of cardioprotective strategies in *all* patients likely results in increased patient burden, negative side effects, reduced adherence, and poor tolerability. In the CECCY RCT, only ~9 to 18% in the carvedilol and placebo groups achieved desired dosages, emphasizing the importance of targeting those who would derive the greatest benefit.[14]
Our scientific premise is that there is a critical need to develop personalized strategies to identify and treat patients at increased risk for cancer therapy CTX. A biomarker guided strategy in cardio-oncology has the potential to improve CV and oncologic care. Risk stratification can identify subgroups that would derive the most benefit from pharmacologic cardioprotection; prevent the interruption or cessation of life-saving cancer therapies secondary to CTX; improve long-term CV outcomes; and enable effective utilization of cancer and CV therapies. Biomarker-based strategies to identify high CV risk individuals for preventive or therapeutic interventions are used in everyday practice (e.g. lipid profiles, troponins [Tn], natriuretic peptides [NT-proBNP]),[24,25] but not yet in cardio-oncology.

Previously reported biomarker studies in cardio-oncology focused primarily on Tn screening and suffer from a lack of external validity. A body of work in cardioprotection prophylaxis with ACE-I based upon Tn in patients receiving high dose chemotherapy has failed to be replicated. Our own Preliminary Data (Section 2.2.2.1) do not provide consistent evidence to support the use of Tn. In contrast, our data support a potential role for natriuretic peptides, and motivate this investigation into the potential benefit of natriuretic peptide screening in cardio-oncology. Indeed, natriuretic peptide screening in a general population at risk for HF suggests potential benefit: STOP-HF suggested that natriuretic peptide screening in at-risk populations, as compared to standard care, resulted in a significant reduction in LV systolic and diastolic dysfunction and HF.[26]

2.2.2 Preliminary Data

2.2.2.1 Preliminary Results from the Penn CCT Cohort

The Penn Cardiotoxicity of Cancer Therapy (Penn CCT) cohort study (R01 HL118018, NCT01173341, IRB #811155, Ky PI) is a large, single-center longitudinal prospective cohort of breast cancer patients treated with doxorubicin and/or trastuzumab that provides motivation for this proposal. This resource is the foundation of our research into echocardiographic and biomarker phenotyping and CTX risk.[27–35] Our work has consistently demonstrated that there are biologic and functional perturbations that occur within the first year of cancer therapy, providing rationale for the use of cardioprotection during this period. The average nadir in LVEF change at 1 year in those receiving doxorubicin with or without trastuzumab is 5.1%, occurring at a median of 4.6 months (mean 5.5 months). At 1 year, 9.7% suffer from CTX; 3.5% have NYHA Class II/III HF.

Confidential and Proprietary Information of the University of Pennsylvania

Figure 2: Changes in LVEF with Doxorubicin and/or Trastuzumab. Changes in LVEF over 3 years from cancer therapy initiation. Loess curves (95% CI) for treatment groups.

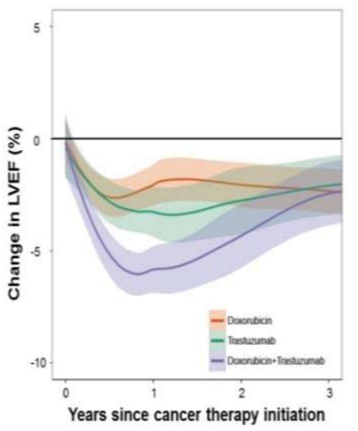

Figure 3: Changes in E/e' with Doxorubicin and/or Trastuzumab. Changes in E/e' over 4 years from cancer therapy initiation. Loess curves (95% CI) for treatment groups.

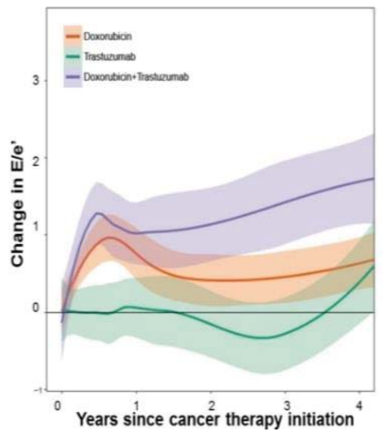

In Penn CCT, we quantified the severity of LVEF declines over time with doxorubicin and/or trastuzumab (Figure 2). Our findings, published in Circulation (Dr. Ky PI, Dr. Margulies co-I), indicate that systolic volumes, longitudinal and circumferential strain, and VA coupling indices (Ea/Ees) determine LVEF decline and recovery with doxorubicin and/or trastuzumab.[29] We have also used this cohort to determine that persistent reductions in measures of diastolic function, and increases in E/e' ($p<0.01$) occur with doxorubicin (**Figure 3**).[36]

Figure 4: Changes in hsTnT and NT-proBNP with Doxorubicin and/or Trastuzumab. Changes biomarkers over 3 years from cancer therapy initiation. Loess curves (95% CI) displayed for treatment groups.

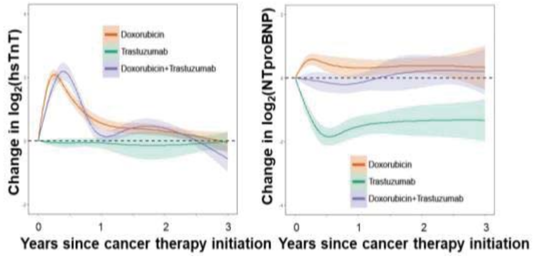

These changes are predictive of subsequent LVEF declines. Our published findings in *JACC Imaging* also suggest that doxorubicin results in early and persistent abnormalities in 3D strain (global circumferential, longitudinal, principal strain).[27] Altogether, these studies support the hypothesis that changes in cardiac function tend to occur early (within 1 year) and are detectable. Our proposed pharmacologic strategies to attenuate NT-proBNP elevations have also been shown to result in improvements in all of these indices (LVEF, volumes, diastolic function) in HF[37–39] and in CCT (Figure 4).

We have enrolled 625 patients into the Penn CCT cohort study. Our findings and productivity support the scientific premise and feasibility of this proposal. Our analyses identify changes in high-sensitivity TnT (hsTnT) and NT-proBNP with cancer therapy (Figure 4). NT-proBNP is strongly associated with CTX risk[31] (Table 1). Baseline biomarker measures were not consistently associated with CTX. Moreover, TnT was not consistently associated with CTX risk, except at a single time point post-doxorubicin completion. Our data do not support the strategy of routine hsTnT screening. In contrast, we have observed consistent associations between increases in NT-proBNP and LVEF declines. We propose that routine serial assessment of NT-proBNP has the greatest potential utility in the surveillance of cancer patients treated with anthracyclines. The frequency of NT-proBNP elevations, associations with CTX at 1 year, and test characteristics

according to any prior NT-proBNP elevation >125 ng/L and >150ng/L are shown in **Table 1** and **Table 2.** Based upon the frequency and timing of elevations and test characteristics, we favor a cut point that is more sensitive, and our PPV of 33.5% is 3-fold greater than the baseline risk of 9.7%. However, while NT-proBNP of 125ng/L is the upper limit of normal (ULN) for patients less than 75 years of age, this increases to 450ng/L in patients 75 and older. Although the cohort below did not include patients over 75 years, there are no data to suggest that patients 75 years and older would benefit from initiation of neurohormonal blockade with an NT-proBNP within normal limits. We propose an NT-proBNP > ULN (NTproBNP > 125 ng/L for patients < 75 years old and NT-proBNP > 450 ng/L for patients 75 years old and older) as a discriminator of increased CV risk.

Table 1: Test Characteristics According to NT-proBNP Thresholds

Biomarker Threshold	HR (95% CI)	Sensitivity (%)	Specificity (%)	PPV (%)	NPV (%)
NT-proBNP>125	1.74 (1.08, 2.82)	39.4	78.2	33.5	82.2
NT-proBNP>150	1.97 (1.19, 3.26)	28.6	89.1	42.2	81.7

Table 2: Frequency of NT-proBNP Biomarker Elevations

Biomarker Threshold	N (%)		Days to earliest elevation from baseline (Median (IQR))	
	Dox	Dox+Tras	Dox	Dox+Tras
NT-proBNP >125 ng/L	91/199 (45.7)	24/53 (45.3)	31 [0, 70]	55 [0, 99]
NT-proBNP >150 ng/L	69/199 (34.7)	21/53 (39.6)	63 [28, 122]	70 [0, 282]

2.2.2.2 Biomarker Studies in Lymphoma Patients Receiving Anthracyclines

In a multi-center prospective cohort of 586 patients with lymphoma (24%) or breast cancer receiving anthracyclines (PREDICT, NCT01311843, Dr. Ky co-I), patients underwent serial biomarker evaluation at baseline, before each cycle, and at 6 and 12 months. Using point-of-care BNP (not NT-proBNP), 17% had a BNP>100 ng/L and 11% suffered from CTX. BNP>100 ng/L at baseline or during therapy was associated with a > 3-fold odds of developing CTX. The sensitivity associated with a BNP>100 ng/L was 35%, specificity 85%, PPV 22% and NPV 92%.[40] These test characteristics are similar to that shown in Table 2 for breast cancer. These findings support inclusion of lymphoma patients in our pilot RCT. Biologic plausibility and epidemiologic data suggest that the cardiotoxic effects of anthracyclines persist across multiple cancer populations, and we do not expect there to be differential relationships between NT-proBNP and CTX across cancer type. However, the purpose of the R21 is to verify this and extend the external validity of our findings.

2.2.2.3 Neurohormonal Blockade as A Cardioprotective Strategy in High Risk Patients

Biomarker guided strategies to titrate medications in HF have suggested potential promise in chronic HF.[37–39] These widely used, guideline-based medications in HF are highly effective in improving indices of cardiac remodeling, decreasing CV hospitalizations and mortality.[21,41] There are longstanding data in HF to support the use of ACE-I, ARBs, and beta blockers to decrease natriuretic peptide levels.[21,26] Moreover, a subanalysis of our own Penn CCT cohort data support our hypothesis that therapies such as carvedilol prevent the adverse changes in LVEF, longitudinal strain, and NT-proBNP (**Figure 5**).

Figure 5: Effects of Carvedilol on Cardiac Function and Mechanics in Penn CCT Positive changes observed with LVEF and negative changes in longitudinal strain and NT-proBNP in carvedilol treated patients (blue) indicate beneficial changes.

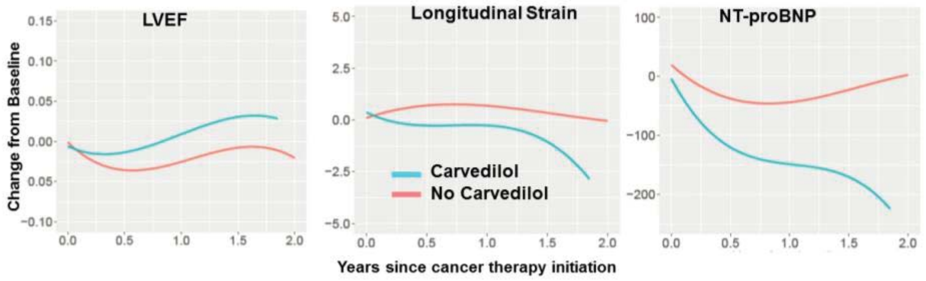

We propose the use of neurohormonal blockade with ACE-I, ARBs, beta-blockers, or aldosterone antagonists as a cardioprotective strategy in high risk patients with elevated NT-proBNP levels given the data in HF that indicate a benefit, particularly in decreasing NT-proBNP levels. We will follow a detailed algorithm to facilitate the administration of these medications.

2.2.3 Pharmacokinetics, Pharmacodynamics and Toxicology

While multiple beta-blockers, ACE-I, ARB and aldosterone antagonists have been studied and used to treat HF, we will prioritize the use of the following formulations: carvedilol (beta-blocker), Lisinopril (ACE-I), candesartan (ARB) and eplerenone (aldosterone antagonist).
Carvedilol, Lisinopril, eplerenone, and candesartan are safe, widely prescribed, guideline recommended, and FDA approved HF therapies with demonstrated benefits in CV outcomes (mortality, HF hospitalizations), remodeling (LVEF, longitudinal and circumferential strain, diastolic function), and patient symptoms.

I. Carvedilol phosphate (Coreg) is an alpha/beta-adrenergic blocking agent indicated for the treatment of: (1) mild to severe chronic heart failure; (2) left-ventricular dysfunction following myocardial infarction in clinically stable patients; and (3) hypertension.

a. PHARMACOLOGY: carvedilol is a racemic mixture in which nonselective beta-adrenergic blocking activity is present in the S(-) enantiomer and alpha-1-adrenergic blocking activity in both the R(+) and S(-) enantiomers. It has no intrinsic sympathomimetic activity. Blocking beta-adrenergic activity causes vasodilation and a reduction in peripheral

vascular resistance which results in a reduction in cardiac output, tachycardia, renin production, and reflex orthostatic tachycardia. Blocking alpha-adrenergic activity results in a reduction in standing blood pressure, more so than supine blood pressure. Vasodilation, increase in stroke volume reduction in pulmonary capillary wedge pressure, pulmonary artery pressure, heart rate, systemic vascular resistance and right arterial pressure may all contribute to the beneficial effects seen in congestive heart failure.

b. PHARMACOKINETICS:

i. Absorption: Carvedilol is rapidly absorbed systemically, and plasma concentrations achieved are correlated to the dose. It is extensively absorbed with a bioavailability of 25-30% due to first-pass metabolism. Food delays the rate but not the extent of absorption. Taking with food can minimize the effects of orthostatic hypotension. The onset and peak of antihypertensive effect are approximately 30 minutes and 1-2 hours, respectively.

ii. Distribution: Carvedilol is highly protein bound (>95%). There is substantial distribution into extravascular tissue with a steady state volume of distribution of 115L.

iii. Metabolism: Carvedilol undergoes extensive hepatic metabolism primarily by cytochrome P450 (CYP) 2D6 and 2C9, with a minor contribution from CTP3A4, 2C19, 1A2, and 2E1 resulting in the formation of 3 active metabolites with beta receptor blocking activity. The metabolite 4-hydroxyphenyl is approximately 13 times more potent than the parent compound for beta-andrenergic blockade, however, concentrations of the metabolites are approximately one tenth that of the parent compound. The 3 active metabolites all exhibit weak vasodilatory activity. Plasma concentrations in the elderly and in those with cirrhotic liver disease are 4-7 times higher.

iv. Elimination: Glucuronidated and sulfated secondary metabolites of carvedilol are excreted primarily through bile into the feces. Following oral administration, the mean systemic elimination half-life ranges from 7-10 hours.

II. Lisinopril (PRINIVIL) is an angiotensin-converting enzyme inhibitor indicated for the treatment of: (1) chronic heart failure; (2) prevention of mortality post myocardial infarction; and (3) hypertension.

a. PHARMACOLOGY: Lisinopril inhibits angiotensin converting enzyme (ACE) in human subjects and animals. ACE is a peptidyl dipeptidase that catalyzes the conversion of angiotensin I to the vasoconstrictor substance, angiotensin II. Angiotensin II also stimulates aldosterone secretion by the adrenal cortex. The beneficial effects of Lisinopril in hypertension and heart failure appear to result primarily from suppression of the renin angiotensin-aldosterone system. Inhibition of ACE results in decreased plasma angiotensin II which leads to decreased vasopressor activity and to decreased aldosterone secretion. ACE is identical to kininase, an enzyme that degrades bradykinin. Whether increased levels of bradykinin, a potent vasodepressor peptide, play a role in the therapeutic effects of Lisinopril remains to be elucidated.

b. PHARMACOKINETICS:

i. Absorption: Lisinopril does not undergo metabolism and is excreted unchanged entirely in the urine. Based on urinary recovery, the mean extent of absorption of Lisinopril is approximately 25 percent, with large inter-subject variability (6-60 percent) at all doses tested (5-80 mg). Lisinopril absorption is not influenced by the presence of food in the gastrointestinal tract.
ii. Distribution: Lisinopril does not appear to be bound to other serum proteins. The absolute bioavailability of Lisinopril is reduced to about 16 percent in patients with stable NYHA Class II-IV congestive heart failure, and the volume of distribution appears to be slightly smaller than that in normal subjects.
iii. Metabolism: Lisinopril does not undergo metabolism.
iv. Elimination: Lisinopril is excreted unchanged entirely in the urine. Impaired renal function decreases elimination of Lisinopril, which is excreted principally through the kidneys, but this decrease becomes clinically important only when the glomerular filtration rate is below 30 mL/min. Following oral administration of PRINIVIL, peak serum concentrations of Lisinopril occur within about 7 hours, although there was a trend to a small delay in time taken to reach peak serum concentrations in acute myocardial infarction patients. Declining serum concentrations exhibit a prolonged terminal phase which does not contribute to drug accumulation. This terminal phase probably represents saturable binding to ACE and is not proportional to dose. Upon multiple dosing, Lisinopril exhibits an effective half-life of 12 hours.

III. Eplerenone (INSPRA) is an aldosterone antagonist indicated for the treatment of: (1) chronic heart failure; and (2) hypertension.

a. PHARMACOLOGY: Eplerenone binds to the mineralocorticoid receptor and blocks the binding of aldosterone, a component of the renin-angiotensin-aldosterone-system (RAAS). Aldosterone synthesis, which occurs primarily in the adrenal gland, is modulated by multiple factors, including angiotensin II and non-RAAS mediators such as adrenocorticotropic hormone (ACTH) and potassium. Aldosterone binds to mineralocorticoid receptors in both epithelial (e.g., kidney) and nonepithelial (e.g., heart, blood vessels, and brain) tissues and increases blood pressure through induction of sodium reabsorption and possibly other mechanisms. Eplerenone has been shown to produce sustained increases in plasma renin and serum aldosterone, consistent with inhibition of the negative regulatory feedback of aldosterone on renin secretion. The resulting increased plasma renin activity and aldosterone circulating levels do not overcome the effects of eplerenone. Eplerenone selectively binds to human mineralocorticoid receptors relative to its binding to recombinant human glucocorticoid, progesterone, and androgen receptors.

b. PHARMACOKINETICS:

i. Absorption: Mean peak plasma concentrations of eplerenone are reached approximately 1.5 to 2 hours following oral administration. Absorption is not affected by food. The absolute bioavailability of eplerenone is 69% following administration of a 100 mg oral tablet. Both peak plasma levels (Cmax) and area under the curve (AUC) are dose proportional for doses of 25 mg to 100 mg and less than proportional at doses above 100 mg. Upon repeat dosing, steady state levels are reached within 2 days.

ii. Distribution: The plasma protein binding of eplerenone is about 50% and it is primarily bound to alpha 1-acid glycoproteins. The apparent volume of distribution at steady state ranged from 42 to 90 L. Eplerenone does not preferentially bind to red blood cells.
iii. Metabolism: Eplerenone metabolism is primarily mediated via CYP3A4. No active metabolites of eplerenone have been identified in human plasma.
iv. Elimination: Less than 5% of an eplerenone dose is recovered as unchanged drug in the urine and feces. Following a single oral dose of radiolabeled drug, approximately 32% of the dose was excreted in the feces and approximately 67% was excreted in the urine. The elimination half-life of eplerenone is approximately 3 to 6 hours. The apparent plasma clearance is approximately 10 L/hr.

IV. Candesartan cilexetil is an angiotensin II receptor (AT1) blocker indicated for the treatment of: (1) chronic heart failure and (2) hypertension.

a. PHARMACOLOGY: Candesartan blocks angiotensin II binding to AT1 receptors. Angiotensin II is formed from angiotensin I in a reaction catalyzed by angiotensin-converting enzyme (ACE, kininase II). Angiotensin II is the principal pressor agent of the renin-angiotensin system, with effects that include vasoconstriction, stimulation of synthesis and release of aldosterone, cardiac stimulation, and renal reabsorption of sodium. Candesartan blocks the vasoconstrictor and aldosterone-secreting effects of angiotensin II by selectively blocking the binding of angiotensin II to the AT1 receptor in many tissues, such as vascular smooth muscle and the adrenal gland. Its action is, therefore, independent of the pathways for angiotensin II synthesis. There is also an AT2 receptor found in many tissues, but AT2 is not known to be associated with cardiovascular homeostasis. Candesartan has much greater affinity (>10,000-fold) for the AT1 receptor than for the AT2 receptor. Blockade of the angiotensin II receptor inhibits the negative regulatory feedback of angiotensin II on renin secretion, but the resulting increased plasma renin activity and angiotensin II circulating levels do not overcome the effect of candesartan on blood pressure

b. PHARMACOKINETICS:

i. Absorption: Candesartan cilexetil, a prodrug, is hydrolyzed to candesartan during absorption from the gastrointestinal tract. Following administration of candesartan cilexetil, the absolute bioavailability of candesartan was estimated to be 15%. After tablet ingestion, the peak serum concentration (Cmax) is reached after 3 to 4 hours. Food with a high fat content does not affect the bioavailability of candesartan after candesartan cilexetil administration.
ii. Distribution: The volume of distribution of candesartan is 0.13 L/kg. Candesartan is highly bound to plasma proteins (>99%) and does not penetrate red blood cells. The protein binding is constant at candesartan plasma concentrations well above the range achieved with recommended doses. In rats, it has been demonstrated that candesartan crosses the blood-brain barrier poorly, if at all. It has also been demonstrated in rats that candesartan passes across the placental barrier and is distributed in the fetus.
iii. Metabolism: Candesartan cilexetil is rapidly and completely bioactivated by ester hydrolysis during absorption from the gastrointestinal tract to candesartan, a selective AT1 subtype angiotensin II receptor antagonist. Candesartan is mainly excreted

unchanged in urine and feces (via bile). It undergoes minor hepatic metabolism by O-deethylation to an inactive metabolite.
iv. Elimination: The elimination half-life of candesartan is approximately 9 hours. After single and repeated administration, the pharmacokinetics of candesartan are linear for oral doses up to 32 mg of candesartan cilexetil. Candesartan and its inactive metabolite do not accumulate in serum upon repeated once-daily dosing.

Participants who are being treated with beta-blockers, ACE-I/ARB and/or aldosterone antagonists for a pre-existing condition (e.g hypertension) will not be excluded from the study; pre-existing medications (Table 3) will be uptitrated in the intervention arm as consistent with clinical guidelines.

Table 3: Acceptable beta-blockers, ACE-I/ARBs, and aldosterone antagonists

Beta Blocker	ACE-I	ARB	Aldosterone Antagonist
Acebutolol	Benazepril	Azilsartan	Spironolactone
Atenolol	Captopril	Eprosartan	
Betaxolol	Cilazapril	Irbesartan	
Bisoprolol	Enalapril	Losartan	
Carteolol	Enalaprilat	Olmesartan	
Esmolol	Fosinopril	Telmisartan	
Labetalol	Moexipril	Valsartan	
Levobunolol	Perindopril		
Metoprolol	Quinapril		
Nadolol	Ramipril		
Nebivolol	Trandolapril		
Pindolol			
Propranolol			
Sotalol			
Timolol			

2.2.4 Assessment for Potential Study Products Drug-Drug Interactions

Although we expect these neurohormonal antagonists to be safe, as they are widely utilized in HF, we will review toxicities and drug interactions. At each visit, we will assess concomitant medications and adverse events (AEs) with Common Terminology Criteria for Adverse Events (CTCAE) v5.

Study medications will be recorded in the patient's EMR and patients in the intervention arm who are initiated on neurohormonal antagonists will be given a wallet card to share with all providers and pharmacists. Patients will also be instructed to notify the study team of any changes in their medications. Potential drug-drug interactions will be managed on a case by case basis, in consultation with the PI and clinical care team. If a patient is prescribed a medication which may interact with a study medication, the PI or delegated co-investigator will contact the clinical care team to see if there is a clinically appropriate alternative; if there is no

appropriate alternative, the relevant study medication will be substituted for another acceptable drug in the same class which does not pose a drug-drug interaction risk.

2.2.5 Clinical Adverse Event Profile

Clinical adverse events include but are not limited to reduced renal function and elevation of serum potassium.

2.2.6 Dosing Rationale

Patients in the biomarker guided strategy group who have NT-proBNP > ULN at any visit/lab draw prior to the 12-month visit will be referred for a medication initiation/titration visit with the study team/PI-appointed affiliated physician. The PI/treating team will have discretion in determining the optimal medication titration plan based on individual factors. The basic algorithm presented in **Figure 6** will be provided and will form the basis for treatment. This is further detailed in section 7.1.4 and 7.1.5.

This dosing is consistent with guideline directed clinical practice for patients with heart failure[42]. Individuals already treated with beta-blockers, ACE-I/ARB, and/or aldosterone antagonists (**Table 3**) may remain on their current therapies and have the dose titrated based on NT-proBNP levels.

2.3 Risk/Benefit Assessment

2.3.1 Known Potential Risks

I. Risks associated with carvedilol

**Common, May be Serious** *(> 20%)*	**Occasional, May be Serious** *(4-20%)*	**Rare and Serious** (≤ 3%)
Tiredness	Asthenia (weakness or lack of energy)	Syncope (fainting)
Dizziness	Edema (swelling)	Angina (chest pain)
	Bradycardia (low heart rate)	Atrioventricular Block
	Hypotension (low blood pressure)	Erythema multiforme
	Hypertension (high blood pressure)	Stevens-Johnson Syndrome
	Heart Failure	Toxic Epidermal Necrolysis
	Headache	Aplastic Anemia
	Diarrhea	Intraoperative Floppy Iris Syndrome
	Nausea	Visual Changes
	Vomiting	Status Asthmaticus (severe asthma)
	Hyperglycemia (low blood sugar)	
	Hypercholesterolemia (high cholesterol)	

	Arthralgia (joint pain)	
	Cough	
	Rales	

Reproductive risks: Pregnancy category C. Animal (rats and rabbits) reproduction studies have shown adverse events. There is no adequate data on the use of carvedilol in pregnant women. It is not known whether carvedilol is excreted in human milk and infant risk cannot be ruled out. Study will obtain a pregnancy test as part of screening (if subject is able to become pregnant and one is not available in the medical record). Subjects in the Elevated Risk Group assigned to intervention and who are of potential child-bearing status will be instructed to use adequate birth control and will be offered counseling on what this entails. Such subjects will be instructed to notify the study doctor immediately if they are or think they may be pregnant while on study drug. If a patient becomes pregnant while on the study, patient will be taken off study drug; if appropriate, the PI will coordinate transition to other antihypertensive regimen with patient's clinical care team.

Drug interactions: Refer to the current FDA-approved package insert for the most comprehensive and up to date information on drug interactions. Due to potential drug interactions, a complete patient medication list will be reviewed and recorded prior to initiation and at all study visits. Investigational carvedilol will be recorded in subject's EMR at current dose. The following drugs have been reported as inhibitors or inducers of CYP2D6 which is the major P450 enzyme that metabolizes carvedilol: Bupropion (Wellbutrin), Fluoxetine (Prozac), Paroxetine (Paxil), Quinidine (Quinidex), Duloxetine (Cymbalta) and Digoxin. If a patient on the intervention arm with NT-proBNP > ULN is taking a strong or clinically significant moderate inhibitor, carvedilol will not be prescribed, and another acceptable beta-blocker will be selected instead.

II. Risks associated with Lisinopril

**Common, May be Serious**<br><br>*(> 10%)*	**Occasional, May be Serious**<br>*(4-10%)*	**Rare and Serious**<br><br>(≤ 3%)
Hypotension	Syncope	Angioedema
Dizziness	Chest pain	Stevens-Johnson Syndrome
Increased serum creatinine	Hypotension (low blood pressure)	Toxic Epidermal Necrolysis
	Diarrhea	Vasculitis
	Headache	Hemolytic anemia
	Fatigue	Renal Insufficiency
	Vertigo	Aplastic Anemia
	Hyperkalemia	Vision loss
	Gout	Cutaneous pseudolymphoma
	Diabetes Mellitus	
	Cough	

Reproductive risks: Pregnancy category D. Lisinopril can cause injury and death to the developing fetus and should not be used during pregnancy. It is not known if Lisinopril is present in breast milk. Study will obtain a pregnancy test as part of screening (if subject is able to become pregnant and one is not available in the medical record). Subjects assigned to intervention and who are of potential child-bearing status will be instructed to use adequate birth control and will be offered counseling on what this entails. Such subjects will be instructed to notify the study doctor immediately if they are or think they may be pregnant while on study drug. If a patient becomes pregnant while on Lisinopril, patient will be taken off study drug; if appropriate, the PI will coordinate transition to other antihypertensive regimen with patient's clinical care team.

Drug interactions: Refer to the current FDA-approved package insert for the most comprehensive and up to date information on drug interactions. Due to potential drug interactions, a complete patient medication list will be reviewed and recorded prior to initiation and at all study visits. Investigational Lisinopril will be recorded in subject's EMR at current dose.

III. Risks associated with eplerenone

**Common, May be Serious** (> 10%)	**Occasional, May be Serious** (4-10%)	**Rare and Serious** (≤ 3%)
Hyperkalemia	Dizziness	Hyponatremia
Hypertriglyceridemia	Increased serum creatinine	Abnormal vaginal hemorrhage
		Angioedema

Reproductive risks: Pregnancy category B. There are no adequate and well-controlled studies in pregnant women. It is not known if eplerenone is present in breast milk. Study will obtain a pregnancy test as part of screening (if subject is able to become pregnant and one is not available in the medical record). Subjects assigned to intervention and who are of potential child-bearing status will be instructed to use adequate birth control and will be offered counseling on what this entails. Such subjects will be instructed to notify the study doctor immediately if they are or think they may be pregnant while on study drug. If a patient becomes pregnant while on eplerenone, patient will be taken off study drug; if appropriate, the PI will coordinate transition to other antihypertensive regimen with patient's clinical care team.

Drug interactions: Refer to the current FDA-approved package insert for the most comprehensive and up to date information on drug interactions. Due to potential drug interactions, a complete patient medication list will be reviewed and recorded prior to initiation and at all study visits. Investigational eplerenone will be recorded in subject's EMR at current dose. Concomitant use of inhibitors or inducers of CYP3A4 will be carefully monitored in those randomized to intervention and reviewed by the study PI prior to initiation of eplerenone.

The use of an ARB and aldosterone antagonist are part of guidelines directed therapy in heart failure[42]. The risks primarily relate to hyperkalemia and renal insufficiency and we will carefully monitor the patients' basic metabolic panel after initiation of this combination. Patients' basic

metabolic panel will be monitored at 1 week after initiation of combination therapy, followed by monthly for 3 months and then every 3 months while on study. Moreover, this combination will not be used in patients with a creatinine > 3 mg/L.

IV. Risks associated with candesartan:

**Common, May be Serious**<br><br>*(> 10%)*	**Occasional, May be Serious**<br>*(4-10%)*	**Rare and Serious**<br><br>(≤ 3%)
Hypotension	Dizziness	Angioedema
Renal function abnormalities	Hyperkalemia	Agranulocytosis
	Upper respiratory tract infection	Hepatitis
	Headache	Hyponatremia
		Leukopenia
		Neutropenia

Reproductive risks: Pregnancy category D. Candesartan can cause injury and death to the developing fetus and should not be used during pregnancy. It is not known if candesartan is present in breast milk. Study will obtain a pregnancy test as part of screening (if subject is able to become pregnant and one is not available in the medical record). Subjects assigned to intervention and who are of potential child-bearing status will be instructed to use adequate birth control and will be offered counseling on what this entails. Such subjects will be instructed to notify the study doctor immediately if they are or think they may be pregnant while on study drug. If a patient becomes pregnant while on candesartan, patient will be taken off study drug; if appropriate, the PI will coordinate transition to other antihypertensive regimen with patient's clinical care team.

Risk of Study Procedures

Echocardiography is a safe and non-invasive imaging modality. There are no serious risks associated with transthoracic echocardiography. The additional strain imaging acquired during this study poses no foreseeable serious risks to subjects. It is possible that subjects may experience mild, temporary discomfort from the ultrasound probe being pressed against the chest or from lying in the recumbent position for the duration of the study.

Blood draws: Participants in this study will undergo blood sampling for biomarker assessment. Blood may also be drawn as part of screening (if results are not available within the medical record).

Occasionally there are risks associated with blood draws such as bruising, swelling, black and blue marks, fainting and/or infection at the site. Subjects may also experience a decrease in hemoglobin and hematocrit (red blood cell number, called anemia) from having blood drawn frequently. To minimize discomfort and inconvenience, blood sampling will be combined with a routine clinical blood draw whenever possible.

Blood will be collected and banked as follows:

- Baseline: 16ml (4ml SST, 12 ml EDTA)

- 3-, 6, 9, and 12-month visits: 12ml (4ml SST, 8ml EDTA)
- Anthracycline infusion visits (when not combined with another visit): 5.5ml (3.5ml SST, 2ml EDTA)
- These blood draws will occur on both the standard of care and the intervention group. Banked blood will be used to measure NT-proBNP as well as other cardiac biomarkers including Troponin T, GDF-15, and MPO in post-hoc batches. All measurements will be made in a blinded fashion.

For patients in the intervention group, up to 4ml of blood may be drawn at each study visit, anthracycline chemotherapy infusion, and titration visit for clinical measurement of NT-proBNP and metabolic panel; whenever possible, these will be combined with clinical lab draws, so it will not always be necessary to draw 'extra' blood for these tests. We will not collect more that 50mL in an 8-week period for the purposes of this study.

Symptoms Survey: There are no medical risks associated with answering survey questions. However, a patient may become uncomfortable providing personal information. Any question that makes a patient uncomfortable may be skipped.

Other Risks of Participating in Study
All subjects (regardless of arm assignment) will receive the same prospective monitoring. The PI will contact patients with the results of any echocardiogram done for research purposes, and we will reassure patients throughout the study that if there are any clinically significant findings from study procedures, we will tell them and their care team.

Genetic Information: Plasma, serum, and buffy coats from blood samples will be banked for future use. Blood samples banked from this study may be used as part of an exploratory analysis to help generate hypotheses as to if there are any single nucleotide polymorphisms or variations in the exome that are associated with an increased risk of cardiotoxicity development. We may also use blood samples for Whole Genome Sequencing. We will determine the most cost-effective approach to genotyping at the time of analysis as this technology continues to evolve. Given the exploratory nature of these analyses, we do not plan to report any of these results to patients or enter the information into the EMR.

One possible risk is the loss of confidentiality of genetic information. This research includes genetic testing. The researchers believe that the risks that a subject will be identified solely from genetic information are very small, but the risk may change in the future as new methods of tracing information are developed.

There can be a risk to knowing genetic information. New health information about inherited traits that might affect subjects, or their blood relatives could be found during a research study. Although we are not able to know all the risks from taking part in research on inherited traits, we believe that the risks to subjects and their families are very low. Samples will be coded, and results of genetic testing will not be shared with subjects or entered into their medical record. A related possible risk is disclosure of genetic information that could lead to discrimination in insurance or employment. Very rarely health or genetic information could be misused by employers, insurance companies, and others. For example, it could make it harder to get or

Confidential and Proprietary Information of the University of Pennsylvania

keep a job or insurance, or life insurance companies may charge a higher rate based on this information. We believe the chance these things will happen is very small, but we cannot make guarantees.

Time: As a result of taking part in this study, patients may lose time at work or home and spend more time in the doctor's office than usual. As much as possible, study visits will be planned on days subjects will be at the medical center for clinical care and scheduled to minimize the "extra" time patients are asked to spend here.

Other: In addition, there may be other unforeseeable risks and inconveniences associated with participation in this study.

2.3.2 Known Potential Benefits

There may be no benefits to participating in this study.

This study may benefit future cancer patients by furthering efforts to detect early signs of cardiotoxicity and is an important early step in developing biomarker guided cardioprotective strategies for cancer patients.

2.3.3 Assessment of Potential Risks and Benefits

While this study does involve certain risks to subjects, the potential benefits derived from this study significantly outweigh the risks associated with this research.

3 Study Objectives and Endpoints

OBJECTIVES	ENDPOINTS	JUSTIFICATION FOR ENDPOINTS
Primary		
To determine if a biomarker guided strategy using NT-proBNP to identify and treat high CV risk patients is feasible and well tolerated in patients with breast cancer or lymphoma treated with doxorubicin	Recruitment rate (percent of eligible patients who are randomized)<br><br>Retention rate (percent of randomized patients who complete the study per protocol)<br><br>Adherence rate (percent of study activities completed in window)<br><br>Compliance by PROMIS Scale v1.0 for patients in the biomarker-guided arm initiated on neurohormonal antagonists<br><br>Compliance rate by study diary for patients in the biomarker-guided arm with NT-proBNP>ULN initiated on neurohormonal antagonists<br><br>Maximum tolerated dosages of neurohormonal antagonist medications for patients in the biomarker-guided arm with NT-proBNP>ULN<br><br>Rates of grade 2 or greater AEs by CTCAEv5.0	Our primary outcomes of interest are the feasibility, safety, and tolerability of the biomarker guided intervention in the study population.
Secondary		
To determine the change in NT-proBNP with neurohormonal antagonists in patients with an NT-proBNP>ULN in the biomarker guided arm	Change in NT-proBNP following initiation of neurohormonal antagonists in patients with NT-proBNP > ULN in the biomarker guided arm	We want to estimate the treatment effect in the biomarker guided treatment arm.
To determine if the biomarkers guided strategy results in decreased CTX compared to standard care.	Change in LVEF over 12 months<br><br>Incidence of CTX (defined as LVEF decline $\geq$ 10% to < 50%) over 12 months	We employ several outcomes measures to assess for the rate of CTX. Our preliminary data

OBJECTIVES	ENDPOINTS	JUSTIFICATION FOR ENDPOINTS
	Incidence of new/worsened clinical HF (defined as urgent or new office or ED visit or hospitalization of HF, adjudicated by the Clinical HF Events Committee according to ACC/AHA standards) over 12 months<br><br>Frequency of cancer treatment interruptions due to CTX	indicate that these are clinically relevant and are commonly used in the cardio-oncology literature.
Tertiary		
Explore the effect of a biomarker guided strategy on echocardiography derived functional measures	Change in E/e' over 12 months<br><br>Change in longitudinal strain over 12 months<br><br>Change in circumferential strain over 12 months<br><br>Change in VA coupling measures over 12 months	Our preliminary data suggest a possible role as surrogate measurements of CTX
Explore the effect of a biomarker guided strategy on other cardiac biomarkers of interest	Change in high-sensitivity Troponin (hsTnT) over 12 months<br><br>Changes in Growth Differentiation Factor 15 (GDF-15) over 12 months<br><br>Changes in myeloperoxidase (MPO) over 12 months	Our preliminary data suggest a possible role as surrogate measurements of CTX.
Explore the impact of a biomarker guided strategy on patient reported outcomes and quality of life measures	Change in Godin Leisure Time Activity Score over 12 months<br><br>Change in MD Anderson Symptoms Inventory – Heart Failure (MDASI-HF) score over 12 months<br><br>Change in PROMIS-Fatigue Score over 12 months<br><br>Change in PROMIS Global Health	These instruments are well validated and widely used in cardio-oncology, heart failure, and cancer research.

OBJECTIVES	ENDPOINTS	JUSTIFICATION FOR ENDPOINTS
	Change in PRO-CTCAE	
Explore the impact of a biomarker guided strategy on completion of anthracycline chemotherapy	Incidence of treatment interruptions in administration of anthracycline chemotherapy (defined as anthracycline held or discontinued secondary to side effects or toxicity)	We want to assess whether the intervention increases or decreases the total dose of anthracycline administered
Explore the relationship between anthracycline dose and biomarker elevations	The association between anthracycline dose (doxorubicin equivalent) and NT-proBNP levels	

4 Study Plan

4.1 Study Design

We hypothesize that a biomarker guided treatment strategy that initiates neurohormonal antagonists in breast cancer and lymphoma patients who develop increases in NT-proBNP prior to, during, or after anthracyclines will be feasible and well-tolerated, and result in attenuation of CTX, compared to standard care. To test this, we propose a randomized, open label pilot study of 115 breast cancer or lymphoma patients treated with doxorubicin randomized to a biomarker guided strategy. Randomization will be 1:1, stratified by cancer type and trastuzumab therapy. Patients in the biomarker guided arm will have NT-proBNP levels checked prior to start of anthracycline chemotherapy, at each anthracycline chemotherapy cycle, and at the 3-month 6-month, 9-month, and 12-month visits. Results of NT-proBNP test will be entered in the EMR and released to patients. A result of NT-proBNP > ULN at any visit (apart from the 12-month "End of Study" visit) will trigger an additional visit and the initiation of neurohormonal antagonists based on the algorithm described in **Figure 6**, although personalization of algorithm will be allowed. Medications will be titrated as needed and continued through the 12-month visit. At 12 months, medications will be discontinued or continued at the discretion of the treating provider; the medication plan will be discussed with the patient and the patient's clinical care team.

If NT-proBNP is not available at all, and only BNP is available, we will use the ULN of BNP as the cutpoint for titration in the biomarker guided strategy group.

No interim analyses are planned for this pilot study.

4.2 Scientific Rationale for Study Design

There will be no placebo control given this is a feasibility study. The standard care group will not receive a prospective intervention. The effects of neurohormonal antagonist therapy on clinical characteristics are secondary outcomes, and will be further explored in a future randomized, placebo-controlled trial.

Stratified randomization by cancer type and by trastuzumab will ensure balance with respect to these and treatment-related factors between arms; block randomization will be used and will help ensure baseline characteristics are similarly distributed.

4.3 Justification for Dose

Patients in the biomarker guided group will be referred for neurohormonal antagonist initiation at the first NT-proBNP > ULN (**Figure 6**). This NT-proBNP cutoff is based on preliminary data from our group (**Table 1 and Table 2**) and the treatment algorithm is based on guidelines for the treatment of HF.[42]

4.4 End of Study Definition

A participant is considered to have completed the study if he or she has completed all phases of the study including the last visit or the last scheduled procedure shown in the Schedule of Activities (SoA), Appendix Section 12.1.

5 Study Population

5.1 Inclusion Criteria

In order to be eligible to participate in this study, an individual must meet all the following criteria:

- Provision of written informed consent and HIPAA authorization
- Stated willingness to comply with all study procedures and availability for the duration of the study
- Male or female, ≥ 18 years of age
- Diagnosed with breast cancer or lymphoma (any subtype), planned to receive an anthracycline based chemotherapy regimen. Patients may be enrolled up to their first dose of anthracycline even if they have already received other chemotherapeutic or targeted agents as part of neo-adjuvant or adjuvant systemic therapy.
  Note: The treating oncologist will determine whether patient will receive anthracyclines.

5.2 Exclusion Criteria

An individual who meets any of the following criteria will be excluded from participation in this study:

- Diagnosed with Stage IV breast cancer
- Uncontrolled blood pressure defined by SBP > 180mmHg on two or more occasions and taking three or more antihypertensives within 1 month prior to enrollment.
- Baseline systolic blood pressure < 90mmHg within 1 month prior to enrollment (if multiple blood pressures are available in the medical record within 1 month prior to enrollment, the average SBP will be considered)
- Women must not be pregnant or breast-feeding due to the potential harm to an unborn fetus and possible risk for adverse events in nursing infants with some anti-hypertensives, including angiotensin receptor blockers. All females of childbearing

potential must have a blood test or urine study within 10 days prior to enrollment to rule out pregnancy. All females of childbearing potential must be strongly advised to use accepted and effective methods of contraception or to abstain from sexual intercourse for the duration of their participation in the study. A female of childbearing potential is defined as any woman, regardless of sexual orientation or whether they have undergone tubal ligation, who meets the following criteria: 1) has achieved menarche at some point, 2) has not undergone a hysterectomy or bilateral oophorectomy; or 3) has not been naturally postmenopausal (amenorrhea following cancer therapy does not rule out childbearing potential) for at least 24 consecutive months (i.e., has had menses at any time in the preceding 24 consecutive months).

- Patient with prior or concurrent malignancy whose natural history of treatment, in the opinion of the investigator, has the potential to interfere with the safety or efficacy assessment of the investigational regimen
- Patient must not have any of the following
    - Severe hepatic impairment, defined as serum bilirubin > ULN, or AST or ALT > 5.0 ULN on most recent labs prior to enrollment. Results of serum bilirubin, AST, and ALT must be checked for screening if no results available in the EMR within 28 days prior to enrollment.
    - end-stage renal failure on dialysis
    - hyperkalemia with a potassium > 5.5 mEq/l on most recent labs prior to enrollment. Serum potassium must be checked for screening if no results available in the EMR within 28 days prior to enrollment.
    - a history of kidney transplant
    - an eGFR < 30 ml/min/1.73m^2 at most recent check prior to enrollment. Creatinine must be checked for screening if no results available in the EMR within 28 days prior to enrollment
    - cardiogenic shock
    - decompensated heart failure requiring the use of IV inotropic therapy
- Non-English speaking

Note: Patient with a noted severe allergic reaction or absolute contraindication to one medication or medication class listed in **Table 3** is not necessarily excluded from this trial; treatment plans for patients with any allergy or contraindication to a proposed medication will be made in consultation with the Study PI. Another class of agents will be used if there is a severe or absolute contraindication to one of the medicines in one class. Moreover, the patient treated with pre-existing medications that may interact with proposed BP medications is not necessarily excluded; the clinical significance of any potential drug interactions can also be addressed with the Study PI.

5.3 Lifestyle Considerations

Not applicable.

5.4 Screen Failures

A minimal set of screen failure information is required to ensure transparent reporting of screen failure participants, to meet the Consolidated Standards of Reporting Trials (CONSORT) publishing requirements and to respond to queries from regulatory authorities. As this is a pilot study in which feasibility is a primary objective, we will define a screen failure as a patient with breast cancer or lymphoma who is scheduled to receive treatment with anthracyclines at a participating site and whose record is reviewed for potential eligibility. A screening log will be maintained in a REDCap database with access controlled by the PI. No identifiable or demographic information will be retained, but we will record eligibility (yes/no), consent status (yes/no), randomization status (yes/no), reason for ineligibility, and reason for declining.

For patients who sign consent and are not subsequently randomized, we will additionally retain minimal information, including demography, any screening procedures completed, and any serious adverse event (SAE).

Patients who are randomized and subsequently have a change in treatment plan so that they will not receive any dose of anthracyclines will be considered screen failures post-consent and will be withdrawn from the study. We will allow for replacement in this case.

5.5 Strategies for Recruitment and Retention

Patients will be recruited from the breast cancer and hematology/oncology clinics. The study team will use a detailed review of the electronic medical record to identify patients with breast cancer or lymphoma who will be newly beginning treatment with Anthracycline-based therapy and who meet eligibility criteria. After obtaining permission from the treating oncologist, the study team will contact the patient to assess interest and gather additional information about eligibility criteria (as necessary). If interested, patients will be scheduled for a screening visit. The screening visit will consist of consent, a medical history, and pregnancy test (if applicable and none is available in patient's medical record within 10 days), and final determination of eligibility.

Strategies to enhance recruitment include listing the study on the institutions' online registry of clinical trials (searchable by patients and providers); presentations to engage additional oncology providers; and flyers distributed at clinics.

Our patient retention strategies begin with the introduction to the study and rely on our frequent communication with patients about the protocol, the purpose of the study, and the value they bring by participating in research. We have also designed the study to coincide to a significant extent with clinical care to minimize the burden on patients. We plan to send an IRB-approved newsletter to enrolled patients annually until all study activities and primary analyses are complete. The newsletter will update patients on study progress, encourage them to complete study activities, reinforce contact information for the study team, and thank patients for their participation.

The purpose of this study is to understand if a biomarker guided strategy is feasible and if patients are willing to enroll and remain compliant with study procedures. If recruitment is slow

and jeopardizes study goals and milestones, we will use this knowledge to inform the design of the larger Phase III study.

In appreciation for their time and contribution to the study, patients will receive a payment for completing each milestone visit. Payments will be made using the Greenphire Clincard; social security numbers will be collected for this purpose only. Patients will receive $25 for the baseline, 3-, 6, 9, and 12- month visits. In addition, the study will use the Clincard to reimburse for parking if the study visit occurs on a day when the patient has no other appointments.

6 Study Intervention

6.1 Study Intervention

6.1.1 Study Intervention Description

We propose a biomarker guided intervention in which patients have NT-proBNP levels checked prior to start of anthracycline chemotherapy, at each cycle of anthracycline chemotherapy, and at the 3, 6, 9, and 12-month visits. If NT-proBNP > ULN at any study visit or chemotherapy cycle other than the 12-month visit, patients will be referred for an additional study visit with a delegated physician investigator and initiated on neurohormonal antagonists at the discretion of the physician investigator based on the algorithm in **Figure 6**. Although NTproBNP is preferred, a BNP may be ordered if NTproBNP is not available. In this case, BNP > ULN will trigger initiation/titration of neurohormonal blockade.

6.1.2 Dosing and Administration of Neurohormonal Antagonists

For patients with elevated NT-proBNP, dosing will be based the algorithm presented in **Figure 6** and modified based on patient-characteristics in accordance with guidelines for hypertension/heart failure management.

Participants in the intervention arm with NT-proBNP>ULN at any visit (other than the 12 month visit) will be referred for management. The following algorithm will be recommended but not required, see Section 12.2 for details:

Figure 6: <u>Structured Drug Titration for the Biomarker Guided Strategy Group*</u>
Step 1: Determine if NT-proBNP>ULN at any study visit (except month 12) or any chemotherapy session.
Step 2: If SBP>90mmHg and HR>55bpm then follow the following algorithm:
-Begin Carvedilol 3.125mg twice daily and Lisinopril 2.5mg daily (or Candesartan 4mg daily if ARB preferred to ACE-I)
-Up titrate Carvedilol and Lisinopril (or Candesartan) as tolerated to achieve an NT-proBNP≤ULN ng/L assuming SBP>90mmHg and HR>55. If HR<55 no further up titration of carvedilol. If SBP<90 no further up titration of either therapy
Step 3: At follow-up if NT-proBNP>ULN and SBP>100mmHg after up titration of Carvedilol and Lisinopril (or Candesartan), consideration will be made for eplerenone 12.5mg daily

* ***<u>Additional discretion will be allowed by the cardiology provider.)</u>***

Initiation: If any NT-proBNP>ULN (intervention arm) patient will be referred for a medication initiation visit with the PI/PI-appointed physician proxy. If SBP > 90mmHg and HR>55 bpm, carvedilol 3.125mg twice daily and Lisinopril 2.5mg daily (OR Candesartan 4mg daily) will be initiated.

Titration: Carvedilol and Lisinopril (or candesartan) will be uptitrated in a stepwise fashion (doubling the dose, as tolerated) to achieve an NT-proBNP ≤ ULN. Maximum recommended doses: carvedilol 25mg twice daily; Lisinopril 40mg daily, (candesartan 32mg daily).

Timing: Drug imitation and titration visits will occur within 3 weeks following any NT-proBNP>ULN.

In-person versus telephone visits: Titrations may be performed via the telephone or telemedicine visit, as per standard clinical practice, if there is access to blood pressure and heart rate measures. This will be at the discretion of the cardiologist caring for the patient.

6.2 Preparation/Handling/Storage/Accountability

6.2.1 Acquisition and accountability

Medications will be ordered by a designated physician investigator and filled through the patient's clinical pharmacy.

6.2.2 Formulation, Appearance, Packaging, and Labeling

Medications relevant to this study will be dispensed in tablet form, in standard prescription bottles labeled for the individual study subject. Preparation will follow traditional clinical pharmacy practice, pursuant to a prescription.

The active tablets, as noted above, will be purchased commercially and not altered in any way.

6.2.3 Product Storage and Stability

Product will be stored pursuant to policies at commercial pharmacies.

6.2.4 Preparation

Preparation will follow traditional pharmacy practice, pursuant to a prescription

6.3 Measures to Minimize Bias: Randomization and Blinding

Patients will be randomized to a biomarker guided strategy versus standard care (1:1), using stratification by cancer type and by trastuzumab therapy. We propose stratification by cancer type to ensure balance across the two subgroups. We propose stratification by trastuzumab given varying treatment durations (doxorubicin alone for 4 months; doxorubicin + trastuzumab for 14 months). A block randomization will help to ensure balance.

Patients and providers will not be blinded to intervention arm or to medications initiated given the primary focus of this study is the feasibility of neurohormonal antagonist initiation/titration compared with usual care.

6.4 Study Intervention Compliance

Compliance will be assessed using drug diary, and patient questionnaire. Compliance will be assessed at visits at 3, 6, 9, and 12 months and during additional visits while dose is being titrated. As part of each compliance assessment, patients will also be assessed using the PROMIS Scale v1.0.

Patients will be considered noncompliant if they received < 80% or >120% of the cumulative prescribed dose for any period during the titration phase, or for any two periods on stable dose. If a patient is noncompliant, the study team will re-instruct patients in correct administration of medicine and will counsel patient regarding importance of taking study drug as instructed. If a patient is noncompliant during the titration phase, the dose will not be titrated until patient has been compliant for at least 2 weeks.

6.5 Concomitant Therapy

All concomitant medications will be recorded at each study visit. The clinical significance of any potential drug interactions will be addressed by the Study PI or delegated physician investigator; alternative therapies will be recommended as needed, consistent with clinical guidelines.

6.6 Rescue Medicine

N/A

6.7 Study Intervention Discontinuation and Participant Discontinuation or Withdrawal

6.7.1 Discontinuation of Study Intervention

Discontinuation from intervention does not mean discontinuation from the study, and remaining study procedures should be completed as indicated by the study protocol. If a clinically significant finding is identified (including, but not limited to changes from baseline) after enrollment, the investigator or qualified designee will determine if any change in participant management is needed. Any new clinically relevant finding will be reported as an adverse event (AE). Patients in whom medications are discontinued due to an AE will remain in the study. The data to be collected at the time of study intervention discontinuation will include the following: maximum neurohormonal antagonist doses achieved and reason for intervention discontinuation.

6.7.2 Participant Discontinuation/Withdrawal from the Study

Subjects may withdraw from the study at any time without impact to their care. They may also be discontinued from the study at the discretion of the Investigator for lack of adherence to

intervention or study procedures or visit schedules, or because of AEs/SAEs. The Investigator may also withdraw subjects who violate the study plan, or to protect the subject for reasons of safety or for administrative reasons. It will be documented whether each subject completes the clinical study. Subjects assigned to intervention arm and who withdraw early will have one final visit to follow up regarding adverse events. Subjects may withdraw or be withdrawn from treatment but continue to participate in the observational portion of the study.

6.7.3 Data Collection/Follow-up for Withdrawn Subjects

Subjects assigned to intervention arm who initiate study medication and who withdraw consent to participate in the study during the study intervention will be asked for permission to have the study team contact them approximately 30 days after their final dose of the study drug to assess for adverse events.

Subjects may be withdrawn from treatment but choose to continue to participate in other study activities. In this case, subjects will continue study visits as if they were in the control arm.

For subjects who withdraw and are in the control arm, there will be no final visit and no data will be collected following withdrawal.

An investigator may discontinue or withdraw a participant from the study for the following reasons:

- Significant non-compliance (defined as 3 or more missed study visits, or repeated instances (3 or more) with study medication noncompliance as described above Section 6.4), a patient may be withdrawn from treatment at the PI's discretion.
- If any clinical adverse event (AE), laboratory abnormality, or other medical condition or situation occurs such that continued participation in the study would not be in the best interest of the participant
- Disease progression which prevents study visit attendance

The reason for participant discontinuation or withdrawal from the study will be recorded on the Off-Study Case Report Form (CRF). Subjects who sign the informed consent form and are randomized but do not receive scheduled anthracycline therapy may be replaced. Subjects who sign the informed consent form and are randomized, undergo anthracycline therapy, and subsequently withdraw, or are withdrawn or discontinued from the study, will not be replaced.

6.8 *Lost to Follow-Up*

A participant will be considered lost to follow-up if he or she fails to return for 3 consecutive scheduled visits and is unable to be contacted by the study site staff.
The following actions must be taken if a participant fails to return to the clinic for a required study visit:

- The site will attempt to contact the participant and reschedule the missed visit within 1-2 weeks and counsel the participant on the importance of maintaining the assigned visit schedule and ascertain if the participant wishes to and/or should continue in the study.
- Before a participant is deemed lost to follow-up, the investigator or designee will make every effort to regain contact with the participant (where possible, 3 telephone calls and,

if necessary, a certified letter to the participant's last known mailing address or local equivalent methods). These contact attempts should be documented in the participant's medical record or study file.

- Should the participant continue to be unreachable, he or she will be considered to have withdrawn from the study with a primary reason of lost to follow-up.

7 Study Assessment and Procedures

Table 5: Study Activities by Visit Type

	Pre-screening contact	Screening[1]	Baseline[3]	Anthracycline Infusion Visits	Intervention Visits[5]	3-, 6-, 9- and 12-Month Visits	Final AE Assessment[6]
Assess Interest	X						
Give copy of consent form	X						
Schedule Screening	X						
Consent		X					
Medical History		X					
Serum or Urine Pregnancy		X[2]					
Eligibility		X					
Clinical Data Collection		X	X		X	X	X
Randomization		X					
Questionnaires			X			X	
Family/Social History			X				
Research Blood Draw			16ml	5.5ml[7]		12ml	
Echocardiogram			X[4]			X[4]	
Adverse Event Assessment			X		X	X	X
Clinical NT-proBNP (intervention only)			X	X	X	X	
Compliance Assessment (intervention only)					X	X	X

[1] May be done same day as baseline visit
[2] For women of reproductive potential when a negative pregnancy test is not available in the medical record within the prior 10 days
[3] First day of anthracycline chemotherapy
[4] If patient has standard of care echocardiogram at these time points, the study team may obtain a copy in place of a research echocardiogram
[5] For subjects in the intervention arm with NT-proBNP > ULN. Initiation visit will happen 2 weeks after initial elevated NT-proBNP. Subsequent visits will occur every 2 weeks until titration process completed. Unscheduled Titration visits will occur as needed.
[6] Subjects in the intervention arm started on study medication. Final AE assessment will occur 30-45 days after final dose of medication on study.
[7] Blood draws which occur at anthracycline chemotherapy sessions will occur *prior to* any chemotherapy-related infusion. If an anthracycline cycle occurs in window for the baseline, 3, 6, 9, or 12-month visit, it will be combined with that visit and no additional blood will be collected.

7.1 Study Procedures

7.1.1 Overview of Study Procedures

The exact number and timing of visits and duration of intervention will vary depending on which cancer therapy regimen patients are prescribed by the treating oncologist and their arm assignment.

There are some time points that will apply to all subjects:

- Screening, consent, and baseline visit will take place prior to start of anthracycline chemotherapy. Consent may take place remotely, via the telephone or a video visit.
- Blood will be collected at all anthracycline chemotherapy sessions and banked for research assays
- All subjects will have study visits at 3, 6, 9 months and a final study visit approximately 1 year after the start of chemotherapy

Subjects who are randomized to NT-proBNP guided therapy *with* NT-proBNP>ULN will have study activities at additional time points:

- NTproBNP will be checked at each study visit, and at each anthracycline chemotherapy cycle.
- Subjects will have initiation/titration visits within 2 (+/- 1) weeks of any NT-proBNP>ULN; to the greatest extent possible, these will be done in combination with regularly scheduled clinic visits.
- Laboratory monitoring and vitals check will occur within 2 (+/- 1) weeks after any mediation initiation/titration visits.
- Subjects will remain on study medication until the 12-month study visit (365 days +/- 28 days from first anthracycline treatment).
- Subjects will have a final AE assessment (by phone or in person) 30-45 days after the final dose of medication on study.
- At 12 months, medications will be discontinued or continued at the discretion of the study PI.

Table 5 describes what study activities will be completed at each type of visit.

7.1.2 Screening and Randomization

The screening visit may be done the same day as the baseline visit. Consent may be obtained prior to the screening visit but will not be obtained until study staff has verified patient meets inclusion criteria.

- Consent – will be obtained by research coordinator or attending physician. This can be obtained during a phone or video visit with patient.
- Medical history
- Serum or urine pregnancy test - if woman of reproductive potential and negative pregnancy test is not available in the medical record within the preceding 10 days
- Complete metabolic panel may be drawn for screening purposes if AST/ALT, serum bilirubin, creatinine, and/or potassium are not available in the EMR within the necessary window.

After the screening activities are complete, the PI (or appropriately trained and delegated attending co-I) will confirm eligibility. Subjects will be randomized using a stratified (trastuzumab yes/no; breast cancer/lymphoma) module in REDCap.

7.1.3 Baseline Visit

The baseline visit will take place on the first day of chemotherapy, prior to and during infusion. At this visit, the following activities will be completed:

- Collect clinical data
- Symptoms Questionnaires
- Research blood draw (16 mL)
- Family and Social History
- Research or Standard of Care Clinical Echocardiogram - if the pre-chemo echocardiogram does not include the necessary images of for longitudinal and circumferential strain and 3D analysis of the left ventricle, the study will attempt to arrange for a research echocardiogram to capture these images and ensure that complete echo data is available for all patients at baseline. The research echocardiogram may be done at any time between consent and first anthracycline infusion. For subjects in the doxorubicin and trastuzumab breast cancer treatment group, the baseline echo should occur after last trastuzumab and prior to first anthracycline.
- Patients randomized to the intervention arm will have NTproBNP checked

7.1.4 Intervention Arm: Neurohormonal antagonist initiation visit

Subjects in intervention arm will be asked to come in within 3 weeks after first NTproBNP>ULN for medication initiation. If possible, this will be combined with a clinic or infusion visit. See **Figure 7** for detailed initiation plan. Modifications to the algorithms can be made at the discretion of the cardiology provider.

In individuals not on beta-blocker, ACE-I/ARB or aldosterone antagonist therapy at baseline, individuals will be started on carvedilol 3.125mg twice daily and Lisinopril 2.5mg daily (or candesartan 4mg daily) if systolic blood pressure >90mmHg and HR>55 bpm. Provided necessary labs and vitals are available in the EMR, initiation visit may be conducted remotely (by telephone/telemedicine visit).

Figure 7: Initiation of Study Medication

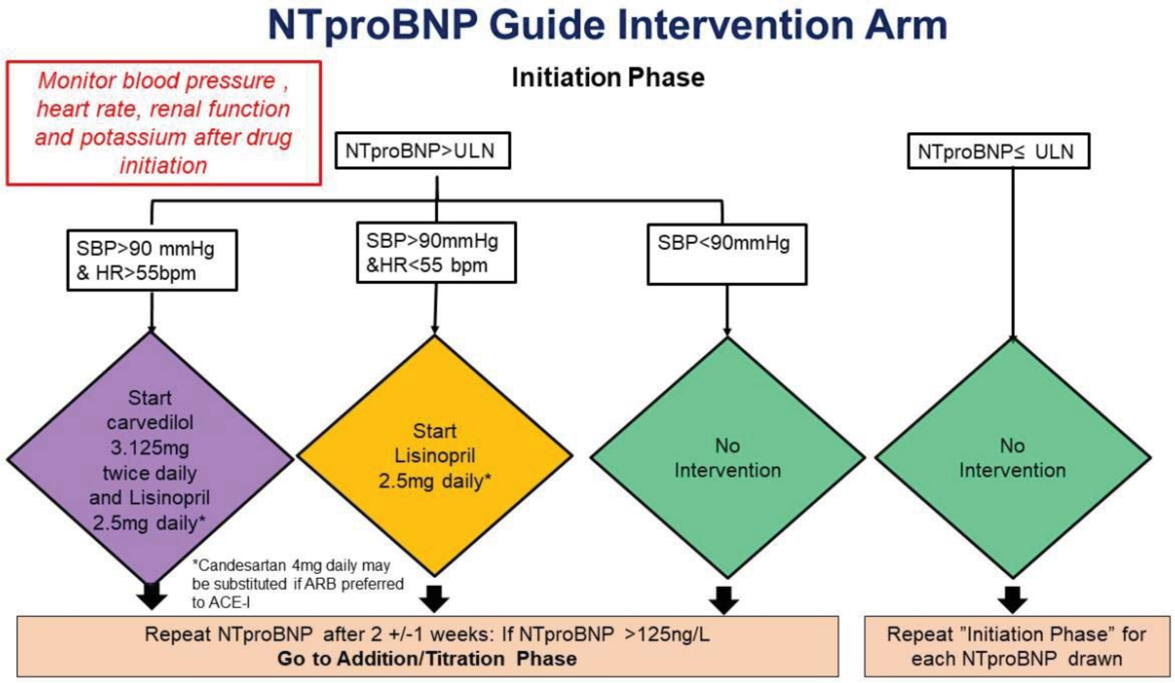

7.1.5 Intervention Arm: Neurohormonal antagonist titration visit

Subjects in intervention arm will have a NT-proBNP collected 2 +/-1 weeks after medication initiation/titration. If a metabolic panel is not available in the EMR in this window, a basic metabolic panel will be performed at the same time. Study team will also assess for medication compliance and AEs/SAEs. Whenever possible, this will be combined with a clinic or infusion visit. Provided necessary labs and vitals are available in the EMR, titration visit may be conducted remotely (by telephone, telemedicine visit, and/or patient portal).

If NT-proBNP > ULN, the dose will be titrated according to **Figure 8**. The dose of carvedilol will be doubled (e.g. 3.125mg to 6.25mg twice daily) and dose of Lisinopril will be doubled (2.5mg to 5.0mg daily) (or candesartan from 4mg to 8mg daily) if **all** below conditions are met:

- SBP ≥ 90mmHg (if multiple blood pressures available since last visit, average SBP will be considered);
- HR ≥ 55 bpm (if multiple heart rates available since last visit, average HR will be considered);
- No AEs/SAEs whose relationship to the study medication is classified as possible, probable, or highly probable; and
- Compliance between 80% and 120% (inclusive)

If HR <55 BPM but SBP >90mmHg Lisinopril or candesartan but not carvedilol will be up titrated. If one or more of the above conditions is not met, PI will determine whether to continue at current dose, up titrate to next dose level or hold one or more study drugs.

If SBP ≥ 100mmHg and NTproBNP>ULN after up titration of carvedilol and Lisinopril (or candesartan), we will consider the addition of eplerenone 12.5mg daily. If a metabolic panel is not available in the medical record, one will be checked 7 days (+/- 3 days) after initiation of eplerenone; then at least monthly for 3 months, and then every 3 months while patient is on study.

Modifications to the algorithms can be made at the discretion of the cardiology provider.

Figure 8: Titration of Study Medication

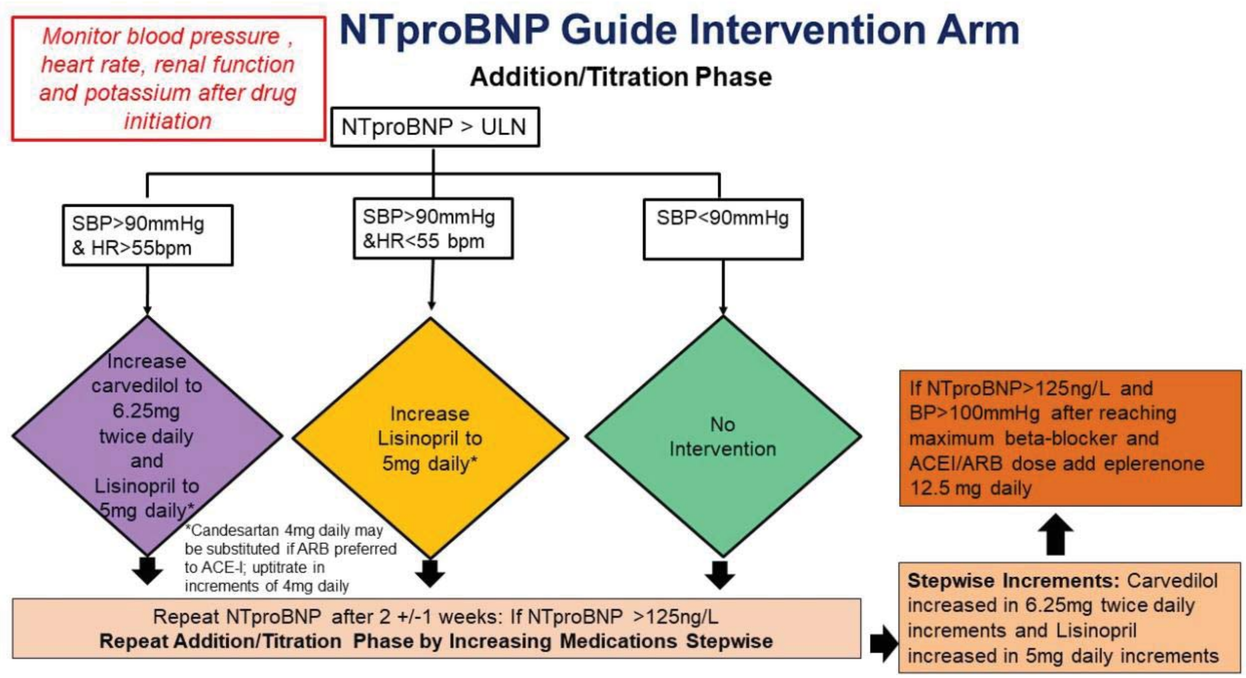

The Addition/Titration process will continue while NTproBNP > ULN or until the provider determines further titration is not appropriate (for example, if patient is already at maximally tolerated therapy or guideline recommended target dose).

If NT-proBNP ≤ ULN, no changes will be made to the study medications and no further titration visits will be scheduled. If a subsequent NT-proBNP > ULN, the Addition/Titration Process will resume.

7.1.6 Intervention Arm: Unscheduled Titration Visit

If excessive (SBP < 90mmHg) and symptomatic hypotension and/or excessive (HR < 40bpm) and symptomatic bradycardia occurs, the patient will be asked to come in for an unscheduled titration visit. At this visit, study team will check vitals, review compliance and AEs/SAEs. Depending upon clinical provider, a BMP or other lab work may be evaluated.

The PI will determine whether to continue at current dose, titrate to next lower dose level, or hold medication, and will determine timing of next titration visit (1-3 weeks).

7.1.7 Anthracycline Infusion Visit

We will arrange for a research blood draw on the day of each anthracycline-based infusion, prior to infusion. At the anthracycline chemotherapy cycle infusion visits, 5.5mL of blood will be collected and banked. In addition, a clinical NT-proBNP will be ordered, drawn and resulted for patients on the intervention arm. Some or all of the anthracycline infusion visits may be combined with the baseline, 3- and/or 6-month visits. When this happens, the additional 5.5mL of blood will not be collected.

7.1.8 3-, 6-, 9-, and 12-Month Visits

The exact timing of the 3-, 6-, 9-, and 12-month visits will depend on patients' treatment regimen. Visits should be completed within +/- 28 days of targeted time point. Some of these visits may be combined with Anthracycline Infusion Visits.

For subjects in the doxorubicin and trastuzumab breast cancer treatment group, the 3-month visit between doxorubicin and trastuzumab should be completed after final treatment with doxorubicin and before first treatment with trastuzumab.

During 3-, 6-, 9-, and 12-month visits, the following study activities will be completed:

- Collect clinical data (including images from standard of care echocardiogram if done)
- Symptoms Questionnaires
- Research blood draw (12 mL)
- AE Assessment
- Compliance Assessment (for subjects randomized to NTproBNP guided intervention on medication)
- Research echocardiogram if no standard of care echocardiogram available at this time point
- NTproBNP (intervention arm only)

At the time of the 12-month visit the study PI or delegated co-investigator will make a recommendation about continuing or discontinuing the neurohormonal antagonist medication regimen for all subjects randomized to the intervention arm who started study medications. This determination will be made on a case by case basis, taking into consideration the results of the echo and NTproBNP, as well as patient's vitals, patient reported symptoms and the patient's changes in cardiac function over the course of the study. If the study investigator recommends that the patient continue the medication regimen, they will discuss with the patient and clinical care team to ensure appropriate transition to clinical follow-up.

7.1.9 Off-Intervention Follow-Up

For subjects randomized to the intervention arm who start study medications, a final AE assessment will be conducted 30-45 days following the final dose of study medication, for AEs/SAEs that occur within 30 days following final dose. For patients who were recommended to continue the medication regimen after completing study intervention, the study team will also confirm that the patient has established care with a provider who will manage medications going forward. This may be done over the phone or in person and will also involve medical record review.

A final compliance assessment may also be done at this time point, if not already completed.

7.1.10 Standard Care Arm

Subjects in the standard care group will have visits, as noted above.; NT-proBNP levels will not be monitored in "real-time" as they are in the Intervention Arm. If a patient on the standard of care arm develops signs or symptoms of anthracycline-induced heart failure, the treating oncologist will determine clinically appropriate follow-up, which may include measurement of NT-proBNP, initiation of cardiac medications, and/or referral to Cardio-Oncology or another cardiologist.

7.2 Study Evaluations and Measurements

7.2.1 Clinical Covariates

Detailed clinical covariates will be obtained from questionnaires and medical record review including:

- Date of birth
- Height (baseline only)
- Weight (all visits when clinical data is collected)
- Any history of disease, and in particular history of cardiac disease (collected at baseline, and updated at each visit when clinical data is collected)
- Blood pressure and heart rate (all visits when clinical data is collected)
- Medication use (all visits when clinical data is collected)
- Clinical lab results not drawn specifically for this study, including pregnancy test, metabolic panel, CBC, and heme profile, BNP/proBNP/NTproBNP, Troponin I/Troponin T, and other cardiac biomarkers if drawn (all visits when clinical data is collected)
- Results of cardiovascular testing, including electrocardiogram, echocardiogram, MUGA, stress tests (all types), cardiac MRI, and cardiac catheterization (all visits which include either clinical data collection or AE assessment)
- BRCA status, if known (at end of study)
- Stage and grade of cancer (at baseline)
- Cancer therapy (including surgery, chemotherapy, non-chemo systemic therapies, and radiotherapy) (at all visits where clinical data is collected)
- Details of hospitalizations and procedures (at all visits where clinical data is collected or AE assessment is performed)
- Family history of cancer and cardiac disease (at baseline)
- Tobacco use (at baseline)

7.2.2 Vital Signs

Blood pressure and heart rate may be taken at all medication initiation/titration visits, including any unscheduled titration visits. Blood pressure will be taken sitting after 5 minutes of rest. If patient reports symptoms of orthostatic hypotension, blood pressure may also be taken standing to evaluate for orthostatic vital signs.

7.2.3 Laboratory Evaluations

NT-proBNP levels will be drawn at all study visits and anthracycline chemotherapy sessions to be measured in the clinical lab in the intervention arm only. If NTproBNP is not available a BNP may be ordered instead. If results of a metabolic panel are not available in the EMR for the time point, a metabolic panel will be obtained at screening and after any initiation/titration of ACE-I, ARB, or aldosterone antagonist.

Research blood samples will be obtained as follows and banked for research purposes:

- Baseline: 16ml (4ml SST, 12 ml EDTA)
- 3-, 6, 9, and 12-month visits: 12ml (4ml SST, 8ml EDTA)
- Anthracycline infusion visits (when not combined with another visit): 5.5ml (3.5ml SST, 2ml EDTA)

Samples will be immediately processed and from these samples, plasma, serum, and buffy coat will be banked for future use. This protocol encompasses the measurement of specific biomarkers, but the objective is also to include these samples in our biobank for future research in cardiotoxicity of cancer therapies and risk-guided interventions, and patients will be consented to have their blood used for this purpose. Blood sampling for research purposes will occur in conjunction with clinical blood draws when possible. Blood sampling will be done in the usual fashion with standard phlebotomy precautions.

NT-proBNP and metabolic panel for patients on the intervention arm will be performed in the local clinical laboratory according to their standard procedures. The results will be entered into the EMR and reviewed by the PI or delegated physician co-investigator. Results of the NT-proBNP and metabolic panel will be available to patients on the intervention arm.

All other assays will be performed post hoc, in batches, to minimize the effects of confounding. These assays will be performed in the Translational Core Lab, in the lab of Dr. Ky in the Smilow Translational Research Center, or in the lab of collaborators. We plan on testing for NTproBNP, hsTnT, MPO, and GDF-15, as well as other cardiac biomarkers to be determined as knowledge advances.

7.2.4 Pregnancy Testing

If a pregnancy test is not available in the medical record within 10 days prior to enrollment, a serum or urine pregnancy test will be performed for subjects of reproductive potential as part of the screening visit.

7.2.5 Other Evaluations, Measures

Transthoracic Echocardiogram:
Resting echocardiograms (Vivid E9 or E95, GE Healthcare) with conventional measures of systolic and diastolic function, in conjunction with post hoc quantitation of novel measures of strain and strain rate will be obtained at study visits when subjects do not have a standard of care, clinically ordered echocardiogram. In addition to the standard echocardiographic protocol used in clinical practice, we will obtain additional imaging, including a more comprehensive assessment of longitudinal and radial measures of cardiac function and contractility via ascertainment of myocardial velocities, strain, and strain rate using high frame rate 2D and color tissue Doppler which will be used for research purposes only. For strain imaging, views over 5 cardiac cycles will be obtained from the 2-, 3-, and 4-chamber LV apical views, as well as via the parasternal short and long axis. These images may add up to 10 minutes to the total acquisition time. Off-line strain analyses will be performed on the EchoPAC system (EchoPAC, GE Healthcare, and Milwaukee, WI) or Tomtec System (Cardiac Performance Analyses). Results of the echocardiogram, according to current clinical standards, will be conveyed to the patient and entered in the patient's chart. Any clinically relevant findings will also be conveyed to the treating provider. In particular, findings that represent a significant cardiac event (such as LVEF reduction to less than 50%) will be conveyed to the treating provider (see Attachment for Echo Protocol). At times, clinical echocardiograms may be obtained as part of standard clinical practice and these studies will be obtained and used for quantitative analysis.

All echocardiograms will be rigorously analyzed by the Penn Center for Quantitative Echocardiography (blinded to clinical characteristics, arm assignment, and treatment assignment). The echocardiographic characterization will generate a rich dataset of measures of systolic function (LVEF, longitudinal and circumferential strain), structure (volumes, mass), diastolic function (E/e'), and VA coupling (Ea/Ees, Ea).

Symptoms and Activity Questionnaires
At baseline and at each study visit, we will collect symptoms, activity and quality of life data from all patients.

The MD Anderson Symptoms Inventory – Heart Failure: The MDASI-HF is a valid and reliable 27-item self-report instrument for symptoms in cancer patients.[45]

PROMIS-Fatigue: The PROMIS Fatigue is a 7-item scale and items assess subjective feelings of tiredness and interference in one's ability to execute daily activities and function. PROMIS-Fatigue has demonstrated stability, internal consistency, and convergence with other relevant scales assessing fatigue.[46–52] The PROMIS-Fatigue instrument is shorter than analogous instruments.

PROMIS Global Health: The PROMIS Global Health measure includes 10 items to assess physical, mental, and social health. Reliability and validation of the PROMIS Global Health measure has been documented.[53]

NCI PRO-CTCAE: The NCI Common Terminology Criteria for Adverse Events (CTCAE) provides criteria for the standardization of adverse event classification. A PRO-CTCAE item

library was developed to improve on the precision and validity of symptomatic adverse event reporting.[54,55] We will administer the following targeted PRO-CTCAE items at all study visits.

- Shortness of breath
- Cough
- Chest Pain
- Arm or Leg Swelling
- Pounding or racing heartbeat (palpitations)
- Fatigue, Tiredness, or Lack of Energy
- Dizziness

Godin Leisure Time Exercise Questionnaire: The Godin Leisure Time Exercise Questionnaire is a self-administered, 4-item instrument assessing the frequency and intensity of physical activity. It is well validated and reliable[56,57].

Drug Diary and Compliance – Subjects randomized to intervention initiated on neurohormonal antagonists only
Subjects will be asked to maintain a drug diary to track doses that are taken/missed, and any symptoms that they experience while on study medication. To minimize confusion regarding individualized dosing, the study team will customize the drug diary for each patient, at each period during the study, and will give patient new drug diaries whenever the dose is changed or when a refill is provided. The drug diary template is attached.

The compliance of subjects randomized to biomarker monitoring and initiated on neurohormonal antagonists will be assessed using the PROMIS Scale v1.0 at each study visit. This is a validated, standardized[58] scale which provides a method for clinicians to assess patients' self-reported compliance levels and to plan appropriate interventions.

7.3 Safety and Other Assessments

At each visit, we will assess for safety as well as for adverse events. Adverse events (CTCAE v5.0 criteria) will be reported according to relevant institutional policies and FDA requirements. Before neurohormonal antagonists are initiated, blood pressure and pulse will be assessed; if either blood pressure or pulse is below the thresholds presented in treatment algorithms Figures 6 and 7, medication doses will not be up-titrated. Additionally, laboratory tests assessing serum potassium and kidney function will be conducted after medication changes.

7.4 Adverse Events and Serious Adverse Events

7.4.1 Definition of Adverse Events (AE)

An adverse event (AE) is any symptom, sign, illness, or experience that develops or worsens in severity during the course of the study. Inter-study illnesses or injuries should be regarded as adverse events. Abnormal results of diagnostic procedures are considered to be adverse events if the abnormality:

- results in study withdrawal
- is associated with a serious adverse event

- is associated with clinical signs or symptoms
- leads to additional treatment or to further diagnostic tests
- is considered by the investigator to be of clinical significance

7.4.2 Definition of Serious Adverse Events (SAE)

Adverse events are classified as serious or non-serious. A serious adverse event is any AE that is:

- fatal
- life-threatening
- requires or prolongs hospital stay
- results in persistent or significant disability or incapacity
- required intervention to prevent permanent impairment or damage
- a congenital anomaly or birth defect
- an important medical event

Planned hospitalizations, such as for mastectomy or inpatient chemotherapy, will not be considered adverse events. However, any events which complicate or prolong planned hospitalizations will be considered SAEs.

7.4.3 Classification of an Adverse Event

7.4.3.1 Severity of Event

Adverse events are classified as serious or non-serious and will be classified based on CTCAE v5.0.

Important medical events are those that may not be immediately life threatening but are clearly of major clinical significance. They may jeopardize the subject and may require intervention to prevent one of the other serious outcomes noted above. For example, drug overdose or abuse, a seizure that did not result in in-patient hospitalization, or intensive treatment of bronchospasm in an emergency department would typically be considered serious.

All adverse events that do not meet any of the criteria for serious should be regarded as non-serious adverse events.

7.4.3.2 Relationship to Study Intervention

The relationship of the study protocol to the event will be classified by the PI or other physician co-Investigator as follows:

- Not related: The event is clearly related to factors such as the participant's clinical state, not with therapeutic interventions associated with the study protocol.
- Remote: The event was most likely related to factors such as the participant's clinical state, not with therapeutic interventions associated with the study protocol.
- Possible: The event follows a reasonable temporal sequence from initiation of the intervention and/or is consistent with known events related to intervention components but is possibly related to factors such as the participant's clinical state.
- Probable: The event follows a reasonable temporal sequence from initiation of the

intervention and/or is consistent with known events related to intervention components and cannot be reasonably explained by factors such as the participant's clinical state.

- Highly Probable: The event follows a reasonable temporal sequence from initiation of the intervention and/or is consistent with known events related to intervention components and cannot be reasonably explained by factors such as the participant's clinical state. In addition, the event occurs immediately following a clinical intervention and/or is consistent with known events related to intervention components.

7.4.3.3 Expectedness

The expectedness of an AE with respect to the study intervention will be classified by the PI or other physician co-investigator as expected if it is a known adverse event of carvedilol, Lisinopril, candesartan, and/or eplerenone (described in the protocol and consent form). An event which is not expected is considered unexpected.

The expectedness of an AE with respect to the condition studied (breast cancer or lymphoma treated with anthracyclines) will be classified by the PI or other physician co-investigator as:

- Not related to underlying condition
- Related to condition studied
- Related to other co-morbidity (co-morbidity will be specified)

7.4.4 Time Period and Frequency for Event Assessment and Follow-Up

At each contact with the subject during the intervention phase and for 30 days following the final dose of study medication, the study team will seek information on adverse events by review of the medical record, specific questioning and, as appropriate, by examination. Information on all adverse events will be recorded immediately in the source document and in the appropriate adverse event module of the CRF. All clearly related signs, symptoms, and abnormal diagnostic procedures results should be recorded in the documentation, although they should be grouped under one diagnosis.

All adverse events occurring during the intervention period will be recorded. The clinical course of each event will be followed until resolution, stabilization, or until it has been determined that the study intervention or participation is not the cause. Serious adverse events that are still ongoing at the end of the intervention period will be followed up to determine the outcome. Any serious adverse event that occurs within 30 days following the final dose medication and is at least possibly related to the study intervention or to study participation will be recorded and reported.

7.4.5 Adverse Event Recording

The following AEs will be recorded in the appropriate CRF in the study database:

- Targeted AEs (as listed in Table 6) grade 2 or higher.
- Expected AEs grade 2 or higher (regardless of attribution)
- Any other AE which is reportable, as defined in Section 7.4.7.

Table 6: CTCAE v5.0 Terms for Cardiovascular and Other Solicited Events

Cardiovascular Disorders	Other Relevant Terms
Chest Pain – cardiac	Acute Kidney Injury
Dyspnea	Cough
Edema Limbs	Dehydration
Hypotension	Dizziness
Hypertension	Dyspnea
Heart Failure	Fatigue
Left Ventricular Systolic Dysfunction	Headache
Palpitations	Presyncope
	Stroke

7.4.6 Adverse Event Reporting

The investigators will conform to the adverse event reporting timelines, formats, and requirements of the various entities to which they are responsible, including the FDA, University of Pennsylvania IRB, and the Abramson Cancer Center CTSRMC.

At a minimum, the below information will be provided at the time of the initial report:

- Study identifier
- Study Center
- Subject number
- A description of the event
- Date of onset
- Current status
- Whether study intervention was discontinued
- The reason why the event is classified as serious
- Investigator assessment of the association between the event and study intervention

Additionally, all other recorded events (unanticipated problems, adverse reactions, and subject complaints) will be reported with respect to institutional and federal policies.

7.4.7 Serious Adverse Event Reporting

AEs/SAEs will be reported to the Penn IRB in accordance with IRB policy (Table 7Table).

Table 7: Penn IRB AE/SAE Reporting Requirements

**Relatedness**	**Expectedness**	**Reportable to Penn IRB**	**When to Report**
Unrelated or Unlikely Related	Expected or Unexpected	**NO**	N/A
Possibly, Probably, or Definitely Related	Expected	**NO**	N/A
Possibly Related	Unexpected	**YES, IF:** the event suggests that the research places subjects at a greater risk than was	EXPEDITED REPORTING WITHIN 10 Business Days

		previously known or recognized	Summarize at Continuing Review
Probably or Definitely Related	Unexpected	**YES**	EXPEDITED REPORTING WITHIN 10 Business Days<br><br>Summarize at Continuing Review
Probably or Definitely Related Death	Unexpected	**YES**	EXPEDITED REPORTING WITHIN 10 Business Days<br><br>Summarize at Continuing Review

All events meeting the Abramson Cancer Center DOCM reporting requirements will also be entered into the PennCTMS AE/SAE form in accordance with DOCM policy.

All grade 3 or higher events, regardless of expectedness or attribution must be reported to the Penn ACC DOMC within 10 days by the Penn coordinating center. All unexpected deaths must be reported within 2 business days of knowledge.

7.4.8 Reporting Events to Participants

It is possible that during the research study, the research staff may notice an unexpected finding(s). Should this occur, the finding(s) will be considered by the appropriate personnel and the site PI will determine if the patient should be informed. These possible finding(s) may or may not be significant and may lead to anxiety about a condition and to further work-up by the subject's physician.

7.4.9 Events of Special Interest

Not applicable.

7.4.10 Reporting of Pregnancy

Women who are pregnant or breast-feeding at the time of screening will not be included in this study. If a pregnancy is confirmed in a subject after enrollment in spite of required regular contraception, and the fetus is exposed to study drug and/or process, the following procedures should be followed to ensure subject safety:
Pregnant patient will be immediately withdrawn from the intervention. Data on fetal outcome are collected for regulatory reporting and drug safety evaluation. Follow-up should be conducted for each pregnancy to determine outcome, including spontaneous or voluntary termination, details of the birth, and the presence or absence of any birth defects, congenital abnormalities, or maternal and/or newborn complications.

7.5 Unanticipated Problems

7.5.1 Definition of Unanticipated Problems (UP)

The Office for Human Research Protections (OHRP) considers unanticipated problems involving risks to participants or others to include, in general, any incident, experience, or outcome that meets all the following criteria:

- Unexpected in terms of nature, severity, or frequency given (a) the research procedures that are described in the protocol-related documents, such as the Institutional Review Board (IRB)-approved research protocol and informed consent document; and (b) the characteristics of the participant population being studied;
- Related or possibly related to participation in the research ("possibly related" means there is a reasonable possibility that the incident, experience, or outcome may have been caused by the procedures involved in the research); and
- Suggests that the research places participants or others at a greater risk of harm (including physical, psychological, economic, or social harm) than was previously known or recognized.

7.5.2 Unanticipated Problem Reporting

Unanticipated problems (UPs) such as:

- Post-marketing withdrawal of a drug, device, or biologic used in a research protocol due to safety concerns.
- FDA ban of a drug, device, or biologic used in a research protocol due to safety concerns.
- Complaint of a participant when the complaint indicates unexpected risks, or the complaint cannot be resolved by the research team
- Breach of confidentiality
- Incarceration of a participant when the research was not previously approved under Subpart C and the investigator believes it is in the best interest of the subject to remain on the study
- Premature closure of a study (e.g., due safety, lack of efficacy, feasibility, financial reasons, etc.)

Such events should be reported by the investigator to the reviewing Institutional Review Board (IRB). The UP report will include the following information:

- Protocol identifying information: protocol title and number, PI's name, and the IRB project number;
- A detailed description of the event, incident, experience, or outcome;
- An explanation of the basis for determining that the event, incident, experience, or outcome represents an UP;
- A description of any changes to the protocol or other corrective actions that have been taken or are proposed in response to the UP.

To satisfy the requirement for prompt reporting, UPs will be reported using the following timeline:

- UPs that are serious adverse events (SAEs) will be reported as any other SAE.
- Any other UP will be reported to the IRB within 10 business days of the investigator becoming aware of the problem.

- All UPs should be reported to appropriate institutional officials (as required by an institution's written reporting procedures), the supporting agency head (or designee), and the Office for Human Research Protections (OHRP) in accordance to the institutional policy.

Applicable events can be reported to the FDA using Form FDA3500A or in narrative format. The report must be sent to the correct division. Specific information that must be included in the reports can be found in 21 CFR 312.32 or in 21 CFR 812.150.
Reports should be submitted to the Center for Drug Evaluation and Research (CDER) at 1-800-FDA-1088

Central Document Room
Center for Drug Evaluation and Research
Food and Drug Administration
5901-B Ammendale Rd
Beltsville, MD 20705-1266

7.5.3 Reporting Unanticipated Problems to Participants

Not applicable.

8 Statistical Considerations

8.1 Statistical Hypotheses

A biomarker guided strategy of neurohormonal antagonist initiation and titration will be feasible (recruitment, retention rate), tolerable (quality of life) and have minimal adverse events (AEs). Our secondary hypothesis is that initiation of neurohormonal antagonists in breast cancer and lymphoma patients will lead to a reduction of cardiotoxicity, cancer treatment interruptions, and clinical heart failure.

8.2 Sample Size Determination

One main objective is to establish feasibility of a large-scale clinical trial. We therefore base estimates for this pilot study on the prevalence of safety outcomes with adequate precision and the power to detect a change in NT-proBNP and LVEF in the biomarker guided, treated arm. In total, we seek to enroll up to 115 patients to randomize up to 102 eligible subjects. This targeted sample size yields an estimated 50 patients in the biomarker guided strategy, and of these, we expect 23 to have elevated NT-proBNP levels > ULN. Assuming an incidence of 12% for all adverse events (hypotension, dizziness, fatigue, bradycardia) with neurohormonal antagonists[14], we will be able to estimate the 95% CI for the incidence rate of all adverse events with a half-width of 9.7%. We have 80% power (2-sided p-value= 0.05) to detect a 0.68 change in NT-proBNP on a log scale (assuming a mean of 0.2 and SD of 0.68) with treatment in the biomarker guided arm (NT-proBNP>ULN), and a 7.9% absolute difference in LVEF between the treated biomarker guided arm (NT-proBNP>ULN) and standard care.

8.3 Populations for Analyses

An intent-to-treat approach will be used in all analyses. The intent-to-treat population used for our primary statistical analyses will include all randomized patients. We will use a 2-sided p-

value of 0.05 in all tests. Within each study arm, we will quantify recruitment and retention, proportion of patients with NT-proBNP>ULN at any point during follow-up, adherence and maximum tolerated dosages of neurohormonal antagonists in the patients in the biomarker guided arm with an NT-proBNP>ULN (study diary, PROMIS Compliance questionnaire), and adverse events (CTCAE v5). We will define these rates and specifically compare rates between the biomarker guided strategy arm and the standard care arm, using t-tests for continuous variables and chi-squared tests for categorical variables, or non-parametric analogs as appropriate. Stratified block randomization by cancer type and by trastuzumab will ensure balance with respect to these and treatment-related factors between arms; block randomization will help ensure baseline characteristics are also similarly distributed.

8.4 Statistical Analyses

8.4.1 General Approach

The first step will involve summarizing the characteristics of all patients (and according to study arm, risk) using standard descriptive statistics (means, standard deviations (SD), medians, interquartile ranges for continuous variables, counts, proportions for categorical variables) and graphical methods. An intent-to-treat approach will be used in all analyses. The intent-to-treat population used for our primary statistical analyses will include all randomized patients. We will use a 2-sided p-value of 0.05 in all tests

8.4.2 Analysis of the Primary Efficacy Endpoint(s)

Within each study arm, we will quantify recruitment and retention, proportion of patients with NT-proBNP>ULN at any point during follow-up, adherence and maximum tolerated dosages of neurohormonal antagonists in the patients in the biomarker guided arm with an NT-proBNP>ULN (study diary, PROMIS Compliance questionnaire), and adverse events (CTCAE v5). We will define these rates and specifically compare rates between the biomarker guided strategy arm and the standard care arm, using t-tests for continuous variables and chi-squared tests for categorical variables, or non-parametric analogs as appropriate. Stratified block randomization by cancer type and by trastuzumab will ensure balance with respect to these and treatment-related factors between arms; randomization will help ensure baseline characteristics are also similarly distributed.

8.4.3 Analysis of the Secondary Endpoint(s)

We will determine the magnitude of NT-proBNP change with neurohormonal antagonists in the biomarker guided arm. We will generate preliminary efficacy estimates for the effect of a biomarker guided strategy on LVEF change; CTX incidence (LVEF decline ≥10% to <50%); clinical HF; frequency of treatment interruptions due to CTX to obtain effect size and variance estimates to inform the design of a subsequent Phase III RCT. We will explore changes in longitudinal and circumferential strain, E/e’, VA coupling (Ea/Ees) to understand their potential role as surrogate outcomes in a subsequent RCT. Time-to-event analyses of secondary outcomes (CTX, HF, treatment interruptions) will be conducted using log rank tests to compare arms; with censoring at last follow-up or death. We will explore comparisons between those patients with an elevated NT-proBNP>ULN in the biomarker guided arm versus standard care, and according to cancer type, trastuzumab, or sex.

8.4.4 Safety Analyses

One main objective is to establish feasibility of a large-scale clinical trial. We therefore base estimates for this pilot study on the prevalence of safety outcomes with adequate precision and the power to detect a change in NT-proBNP and LVEF in the biomarker guided, treated arm. In total, we seek to enroll up to 115 patients. This targeted sample size yields an estimated 50 patients in the biomarker guided strategy, and of these, we expect 23 to have elevated NT-proBNP levels > ULN (**Table 2**). Assuming an incidence of 12% for all adverse events (hypotension, dizziness, fatigue, bradycardia) with neurohormonal antagonists[16], we will be able to estimate the 95% CI for the incidence rate of all adverse events with a half-width of 9.7%. We have 80% power (2-sided p-value= 0.05) to detect a 0.68 change in NT-proBNP on a log scale (assuming a mean of 0.2 and SD of 0.68) with treatment in the biomarker guided arm (NT-proBNP>ULN), and a 7.9% absolute difference in LVEF between the treated biomarker guided arm (NT-proBNP>ULN) and standard care

8.4.5 Baseline Descriptive Statistics

The first step will involve summarizing the characteristics of all patients (and according to study arm, risk) using standard descriptive statistics (means, standard deviations (SD), medians, interquartile ranges for continuous variables, counts, proportions for categorical variables) and graphical methods.

8.4.6 Planned Interim Analyses

N/A

8.4.7 Sub-Group Analyses

We will explore comparisons between those patients with an elevated NT-proBNP>ULN in the biomarker guided arm versus standard care, and according to cancer type, trastuzumab, or sex.

8.4.8 Tabulation of Individual Participant Data

Individual participant data will not be listed.

8.4.9 Exploratory Analyses.

We will explore changes in longitudinal and circumferential strain, E/e’, VA coupling (Ea/Ees) to understand their potential role as surrogate outcomes in a subsequent RCT.

9 Supporting Documentation and Operational Considerations

9.1 Regulatory, Ethical, and Study Oversight Considerations

9.1.1 Informed Consent Process

9.1.1.1 Consent/Assent and Other Informational Documents Provided to Participants

Consent forms describing in detail the biomarker guided strategy, study procedures, and risks are given to the participant and written documentation of informed consent is required prior to starting intervention.

9.1.1.2 Consent Procedures and Documentation

The study will use a combined Informed Consent and HIPAA Authorization Form.

After the subject has been identified by the oncologist, study staff will conduct a phone or in-person interview (script attached) to assess subject interest in the study and review exclusion criteria. If patient is interested and is found to be ineligible, study staff will describe the study in detail to make the patient aware of the procedures, intervention, and time commitment involved with the study. During or immediately following this conversation, the subject will be given a copy of the informed consent form to review (coordinator will email or mail to patient with permission, or will make arrangements to meet patient at next visit). During this conversation, patients will be encouraged to discuss the study with their treating oncologist and with other providers, including primary care provider or cardiologist. Patients will be given the opportunity to ask study staff questions about the study during this conversation and in follow-up conversations. They will also be offered an opportunity to discuss the study with the PI on the phone or in person prior to the screening visit. If interested, patients will be given the option of scheduling the screening visit during this conversation. If they prefer to consider the study further before scheduling the screening visit, study staff will ask for permission to follow-up with the patient at a specified time to see if they would like to schedule the screening visit.

Written consent will be obtained by research coordinator or attending physician investigator in person, in a private exam or consult room. A copy of the signed consent form will be given to the patient. No study procedures will be completed until written consent is obtained. Typically, consent will be obtained at the screening visit. The person obtaining consent will document consent in the electronic medical record.

If no in-person screening visit is necessary, consent may be obtained via remote process. In this case, a phone call or telemedicine visit will be scheduled with the patient to review the consent form; this discussion may include a caregiver at patient's request. If the patient has an email address, signature will be obtained, and consent documented via REDCap's e-consent framework. If the patient does not have access to an email address and time permits, the patient may be sent the ICF/HIPAA form by mail with a return label. In either case, the patient will not be considered to have consented to the study until the team receives a signed ICF/HIPAA form back from the patient.

The person obtaining consent will review the details of the study with the patient and will remind the patient that participation is voluntary and will not change the treatment they would otherwise receive. Person obtaining consent will ensure all questions regarding the study are answered to the patient's satisfaction. Person obtaining consent will document consent at the time the patient signs consent and will include in the documentation how consent was obtained.

9.1.1.3 Optional consent to participate in CCT2 (IRB# 849691 / UPCC 11121, Ky PI) – PENN PATIENTS ONLY

Patients enrolling at Penn who are eligible for both this protocol and IRB# 849691 / UPCC 11121 (CCT2, Ky PI) may consent to participate in CCT2 as an optional study on the #844092 ICF/HIPAA form. There is considerable overlap in study procedures and visit time points between the two studies. The person obtaining consent will review the details of the optional study, including explaining which study visits and activities overlap and which would be done only for the optional study. For patients with lymphoma who are not eligible for the optional

study, the person obtaining consent will explain that this section of the consent does not apply to them. The person obtaining consent will include whether or not patient agrees to participate in the optional study (if applicable) in the consent documentation.

9.1.2 Study Discontinuation and Closure

This study may be temporarily suspended or prematurely terminated by the Sponsor or the PI at any site if there is sufficient reasonable cause. Written notification, documenting the reason for study suspension or termination, will be provided by the suspending, or terminating party to study participants, investigator, funding agency, and regulatory authorities. If the study is prematurely terminated or suspended, the Principal Investigator (PI) will promptly inform study participants, the Institutional Review Board (IRB), and sponsor and will provide the reason(s) for the termination or suspension. Study participants will be contacted, as applicable, and be informed of changes to study visit schedule.
Circumstances that may warrant termination or suspension include, but are not limited to:

- Determination of unexpected, significant, or unacceptable risk to participants
- Insufficient compliance to protocol requirements
- Determination of futility

Study may resume once concerns about safety, protocol compliance, and data quality are addressed, and satisfy the sponsor, IRB and/or Food and Drug Administration (FDA).]
In terminating the study, the Sponsor and the Principal Investigator will assure that adequate consideration is given to the protection of the subjects' interests.

9.1.3 Future Use of Stored Specimens and Data

Blood samples will be obtained for research purposes at baseline and at all study visits. 16mL (4mL SST and 12 mL EDTA) will be obtained at baseline and 12mL (4mL SST and 8mL EDTA) at the subsequent 3, 6, 9 and 12 month visits. At the anthracycline chemotherapy cycle infusion visits, 5.5ml (3.5ml SST and 2ml EDTA) will be collected. Samples will be immediately processed and from these samples, plasma, serum, and buffy coat will be banked for future use. This protocol encompasses the measurement of specific biomarkers, but the objective is also to include these samples in our biobank for future research in cardiotoxicity of cancer therapies and risk-guided interventions, and patients will be consented to have their blood used for the testing of additional novel biomarkers. Blood sampling for research purposes will occur in conjunction with clinical blood draws when possible. Blood sampling will be done in the usual fashion with standard phlebotomy precautions. These specimens will be banked for future use in Dr. Ky's lab at the Perelman School of Medicine (see Attachment for Blood Processing Protocol).

9.1.4 Monitoring

The investigator will allocate adequate time for all monitoring activities. The Investigator will also ensure that the monitor or other compliance or quality assurance reviewer is given access to all the above noted study-related documents and study related facilities (e.g. pharmacy, diagnostic laboratory, etc.), and has adequate space to conduct the monitoring visit.

The investigator will permit study-related monitoring, audits, and inspections by the Abramson Cancer Center DOCM, Penn IRB, government regulatory bodies, and University of Pennsylvania compliance and quality assurance groups of all study related documents (e.g. source documents, regulatory documents, data collection instruments, study data etc.). The investigator will ensure the capability for inspections of applicable study-related facilities. The Penn PI and Data Coordinating Center will perform remote auditing of the participating sites.

9.1.5 Quality Assurance and Quality Control

All monitoring and audits are to be performed according to ICH GCP E6(R2).
The principal investigator and delegated study staff will perform internal quality management of study conduct, data, and biological specimen collection, documentation, and completion. An individualized quality management plan will be developed to describe a site's quality management.
Quality control (QC) procedures will be implemented beginning with the data entry system and data QC checks that will be run on the database will be generated. Any missing data or data anomalies will be communicated to the site(s) for clarification/resolution.
The investigational site will provide direct access to all trial related sites, source data/documents, and reports for the purpose of monitoring and auditing and inspection by local and regulatory authorities.

9.1.6 Data Handling and Record Keeping

9.1.6.1 Data Collection and Management Responsibilities

Each subject will be assigned a unique identifier upon signing consent. Any information linking the subject to his or her subject ID will be maintained locally in a password protected shared-drive; access to this drive will be controlled by the site PI.

Paper copies of study records (including Case Report Forms (CRFs) and source documents) will be stored in a locked cabinet accessible to the study team only.

Enrollment and adverse event data will be recorded in PennCTMS (the University of Pennsylvania's Clinical Trials Management System). All research data will be maintained in a HIPAA compliant study database on REDCap hosted on secure servers at the University of Pennsylvania. Access to the REDCap database will be controlled by the study PI.

Blood samples will be identified by subject ID, visit number, and date collected. Samples will be shipped to the coordinating center and transferred to the lab of Dr. Bonnie Ky at the University of Pennsylvania. The sample log will be maintained electronically using Labvantage on the University of Pennsylvania servers. Access to the study on Labvantage will be controlled by the PI. Results of biomarker analysis will be entered into the REDCap database.

Echocardiographic images may be linked with the patient's EMR, depending on site operations. A clinical report of the echocardiogram will be generated locally and may be linked with the patient's EMR (depending on site operations). Images will be deidentified (pixel and metadata,

using the site's preferred method) and sent on DVD to the Echo Core Lab at the University of Pennsylvania for detailed quantitative analysis. DVDs will be labeled with subject ID, visit number, and date of echocardiogram and stored at the Penn Center for Quantitative Echocardiography. Quantitated echocardiographic data will be labeled with the subject ID, visit number, and date of echocardiogram only and entered into the REDCap database.

9.1.6.2 Study Records Retention

All study records will be maintained as described above for the duration of the study and until the last banked specimen has been exhausted or for at least one year following the final study-related publication or for at least seven years after the close out of the study (whichever is latest). After this time, financial records, source documentation, and paper CRFs may be appropriately destroyed. All versions of the protocol and all signed consent form/HIPAA authorizations will be retained permanently, in keeping with the University of Pennsylvania Records Retention Schedule.

Records in PennCTMS will be retained according to PSOM/UPHS policy.

There may be future questions of interest related to the study of the cardiovascular effects of doxorubicin and/or trastuzumab and risk-guided cardioprotective strategies. The de-identified data or samples collected from this study may be shared with academic collaborators both within and across institutions. In so doing, they will help to answer questions of mutual interest that are related to the overall aims of this study. To that end, subjects will be asked to opt in to allow the future use of their data and future use of their remaining blood samples. If they agree, their blood samples and/or data may be kept (as described above) indefinitely.

9.1.7 Protocol Deviations

A protocol deviation is any change, divergence, or departure from the study design or procedures that is under the investigator's control and that has not been approved by the IRB. A protocol violation is a deviation from the IRB-approved protocol that may affect the subject's rights, safety, or wellbeing, and/or the completeness, accuracy, and reliability of the study data. Protocol deviations/violations include, but are not limited to:

- Enrollment of an ineligible participant
- Follow-up visit at a time point different from that specified in the protocol
- Failure to obtain informed consent
- Failure to keep IRB approval up to date.

The PI and the study team should document all scenarios where the protocol is not followed and provide, in particular:

- Who deviated from the protocol
- What was the deviation
- When did the deviation occur
- How did the deviation happen
- What is the impact of the deviation

- A root cause analysis of why the deviation occurred

If the assessment results in a determination that any of the following are potentially affected, the deviation would be considered of significant impact:

- having the potential to adversely affect subject safety; OR
- increases risks to participants; OR
- adversely affects the integrity of the data; OR
- violates the rights and welfare of participants, OR
- affects the subject's willingness to participate in research.
- there is a potential for an overall impact on the research that should be shared with the IRB for consideration and development of next best steps to address it

Reporting to IRB will follow local requirements.

9.1.8 Publication and Data Sharing Policy

Publication authorship will be based on the relative scientific contributions of the PI, co-Investigators, and key personnel and in accordance with policies of the University of Pennsylvania. Drafting and publication of manuscripts will be carried out in a collaborative manner and pre-defined authorship roles will be set to avoid conflicts and disagreements. The PI will review progress, address concerns, prioritize analysis plans, and discuss publications.

9.1.9 Conflict of Interest Policy

All University of Pennsylvania Investigators will follow the University of Pennsylvania Policy on Conflicts of Interest Related to Research. Investigators at other sites will be expected to report all conflicts of interest to the lead site and to comply with relevant institutional policies at their home institutions

9.2 Protocol Amendment History

Protocol modifications, except those intended to reduce immediate risk to study subjects, may be made only by the Medical Director. A protocol change intended to eliminate an apparent immediate hazard to subjects may be implemented immediately, provided the IRB/IEC is notified within 5 days.

Any permanent change to the protocol must be handled as a protocol amendment. The written amendment must be submitted to the IRB/IEC and the investigator must await approval before implementing the changes. The Medical Director and coordinating center will submit protocol amendments to the appropriate regulatory authorities.

If in the judgment of, the sponsor, the IRB/IEC, and/or the investigator, the amendment to the protocol substantially changes the study design and/or increases the potential risk to the subject and/or has an impact on the subject's involvement as a study participant, the currently approved written informed consent form will require similar modification. In such cases, informed consent will be renewed for subjects enrolled in the study before continued participation.

The table below is intended to capture changes of IRB-approved versions of the protocol, including a description of the change and rationale.

Version	Date	Description of Change	Brief Rationale
2	11/16/2020	NTproBNP will not be monitored in the standard care arm	To avoid biasing the standard of care arm and be consistent with study design of trials of biomarker guided strategy in heart failure
3	07/12/2020	Specify NT-proBNP > ULN is the trigger for initiating neurohormonal blockade in the intervention arm<br><br>Specify that BNP > ULN may be used as an alternative cutoff if NTproBNP is not available.<br><br>Other administrative changes to allow protocol to be opened at other sites	This allows for an age-adjusted cutoff, since the ULN for patients 75 and older is higher than the ULN for younger patients.
3.1	11/29/2021	PENN ONLY – allow patients who are eligible for both this protocol and IRB #849691 / UPCC 11121 (CCT2, Ky PI) to consent to both studies using ICF for this protocol.	Penn's IRB has approved this as an alternate consent process for #849691. There is considerable overlap in study procedures and visit time points between the two studies.
4	2/21/2022	Clarify timing of screening labs (consider lab values closest prior to enrollment, must be checked within 28 days if not available in the EMR)<br><br>Stop performing pill counts<br><br>We will modify the intervention algorithm for patients on the intervention arm with elevated NTproBNP based on our experience implementing it so far.<br>• Emphasize provider discretion in implementing the algorithm;	

Version	Date	Description of Change	Brief Rationale
		• Recommend the use of *either* Lisinopril *or* Candesartan based on provider preference rather than only recommending Candesartan if ACE-Inhibitors are not tolerated;<br>• Add the instruction that *“The Addition/Titration process will continue while NTproBNP > ULN or until the provider determines further titration is not appropriate (for example, if patient is already at maximally tolerated therapy or guideline recommended target dose).”*	
5	1/31/2023	Increase enrollment from 100 to 105 and specify targeting up to 102 patients randomized.	Increasing number of patients consented by up to 5 patients will allow for replacement for patients who screen-failed post-consent (prior to randomization); allowing up to 102 patients to be randomized will allow for some flexibility in coordinating enrollment closure between the multiple sites.
6	7/14/2023	Increase enrollment from 105 to 115, still with the aim of randomizing up to 102 patients	More patients have screen failed post-consent or withdrawn prior to randomization than anticipated. Allowing for up to 115 patients to be enrolled will ensure at least 100 patients are able to be randomized.

Version	Date	Description of Change	Brief Rationale

10 References

1. Swain SM, Whaley FS, Ewer MS. Congestive heart failure in patients treated with doxorubicin: A retrospective analysis of three trials. *Cancer*. 2003;97(11):2869-2879. doi:10.1002/cncr.11407
2. Armenian SH, Lacchetti C, Barac A, et al. Prevention and monitoring of cardiac dysfunction in survivors of adult cancers: American society of clinical oncology clinical practice guideline. *J Clin Oncol*. 2017;35(8):893-911. doi:10.1200/JCO.2016.70.5400
3. Armenian SH, Xu L, Ky B, et al. Cardiovascular disease among survivors of adult-onset cancer: A community-based retrospective cohort study. *J Clin Oncol*. 2016;34(10):1122-1130. doi:10.1200/JCO.2015.64.0409
4. Abdel-Qadir H, Austin PC, Lee DS, et al. A population-based study of cardiovascular mortality following early-stage breast cancer. *JAMA Cardiol*. 2017;2(1):88-93. doi:10.1001/jamacardio.2016.3841
5. van Nimwegen FA, Schaapveld M, Janus CPM, et al. Cardiovascular disease after Hodgkin lymphoma treatment: 40-year disease risk. *JAMA Intern Med*. 2015;175(6):1007-1017. doi:10.1001/jamainternmed.2015.1180
6. Salz T, Zabor EC, Brown P de N, et al. Preexisting cardiovascular risk and subsequent heart failure among non-hodgkin lymphoma survivors. *J Clin Oncol*. 2017;35(34):3837-3843. doi:10.1200/JCO.2017.72.4211
7. Van Nimwegen FA, Ntentas G, Darby SC, et al. Risk of heart failure in survivors of Hodgkin lymphoma: Effects of cardiac exposure to radiation and anthracyclines. *Blood*. 2017;129(16):2257-2265. doi:10.1182/blood-2016-09-740332
8. Shelburne N, Adhikari B, Brell J, et al. Cancer treatment-related cardiotoxicity: Current state of knowledge and future research priorities. *J Natl Cancer Inst*. 2014;106(9). doi:10.1093/jnci/dju232
9. Zamorano JL, Lancellotti P, Muñoz DR, et al. 2016 ESC position paper on cancer treatments and cardiovascular toxicity developed under the auspices of the ESC committee for practice guidelines: The task force for cancer treatments and cardiovascular toxicity of the european society of cardiology (ESC. *Russ J Cardiol*. 2017;143(3):105-139. doi:10.15829/1560-4071-2017-3-105-139
10. Guglin M, Krischer J, Tamura R, et al. Randomized Trial of Lisinopril Versus Carvedilol to Prevent Trastuzumab Cardiotoxicity in Patients With Breast Cancer. *J Am Coll Cardiol*. 2019;73(22):2859-2868. doi:10.1016/j.jacc.2019.03.495
11. Boekhout AH, Gietema JA, Kerklaan BM, et al. Angiotensin II Receptor inhibition with candesartan to prevent trastuzumab-related cardiotoxic effects in patients with early breast cancer a randomized clinical trial. *JAMA Oncol*. 2016;2(8):1030-1037. doi:10.1001/jamaoncol.2016.1726
12. Gulati G, Heck SL, Ree AH, et al. Prevention of cardiac dysfunction during adjuvant breast cancer therapy (PRADA): A 2 × 2 factorial, randomized, placebo-controlled,

double-blind clinical trial of candesartan and metoprolol. *Eur Heart J*. Published online 2016. doi:10.1093/eurheartj/ehw022
13. Pituskin E, Mackey JR, Koshman S, et al. Multidisciplinary approach to novel therapies in cardio-oncology research (MANTICORE 101-Breast): A randomized trial for the prevention of trastuzumab-associated cardiotoxicity. *J Clin Oncol*. 2017;35(8):870-877. doi:10.1200/JCO.2016.68.7830
14. Avila MS, Ayub-Ferreira SM, de Barros Wanderley MR, et al. Carvedilol for Prevention of Chemotherapy-Related Cardiotoxicity: The CECCY Trial. *J Am Coll Cardiol*. 2018;71(20):2281-2290. doi:10.1016/j.jacc.2018.02.049
15. Siegel RL, Miller KD, Jemal A. Cancer statistics, 2017. *CA Cancer J Clin*. 2017;67(1):7-30. doi:10.3322/caac.21387
16. Gong IY, Verma S, Yan AT, et al. Long-term cardiovascular outcomes and overall survival of early-stage breast cancer patients with early discontinuation of trastuzumab: a population-based study. *Breast Cancer Res Treat*. 2016;157(3):535-544. doi:10.1007/s10549-016-3823-y
17. Mehta LS, Watson KE, Barac A, et al. Cardiovascular Disease and Breast Cancer: Where These Entities Intersect: A Scientific Statement From the American Heart Association. *Circulation*. 2018;137(8):e30-e66. doi:10.1161/CIR.0000000000000556
18. Gadi VK, Davidson NE. Practical Approach to Triple-Negative Breast Cancer. *J Oncol Pract*. 2017;13(5):293-300. doi:10.1200/jop.2017.022632
19. Blum JL, Flynn PJ, Yothers G, et al. Anthracyclines in early breast Cancer: The ABC Trials—USOR 06-090, NSABP B-46-I/USOR 07132, and NSABP B-49 (NRG Oncology). In: *Journal of Clinical Oncology*. Vol 35. American Society of Clinical Oncology; 2017:2647-2655. doi:10.1200/JCO.2016.71.4147
20. Echouffo-Tcheugui JB, Erqou S, Butler J, Yancy CW, Fonarow GC. Assessing the Risk of Progression From Asymptomatic Left Ventricular Dysfunction to Overt Heart Failure: A Systematic Overview and Meta-Analysis. *JACC Hear Fail*. 2016;4(4):237-248. doi:10.1016/j.jchf.2015.09.015
21. Yancy CW, Jessup M, Bozkurt B, et al. 2013 ACCF/AHA guideline for the management of heart failure: A report of the American college of cardiology foundation/american heart association task force on practice guidelines. *J Am Coll Cardiol*. 2013;62(16). doi:10.1016/j.jacc.2013.05.019
22. Felker GM, Thompson RE, Hare JM, et al. Underlying causes and long-term survival in patients with initially unexplained cardiomyopathy. *N Engl J Med*. 2000;342(15):1077-1084. doi:10.1056/NEJM200004133421502
23. Chen J, Long JB, Hurria A, Owusu C, Steingart RM, Gross CP. Incidence of heart failure or cardiomyopathy after adjuvant trastuzumab therapy for breast cancer. *J Am Coll Cardiol*. 2012;60(24):2504-2512. doi:10.1016/j.jacc.2012.07.068
24. Stone NJ, Robinson JG, Lichtenstein AH, et al. 2013 ACC/AHA guideline on the treatment of blood cholesterol to reduce atherosclerotic cardiovascular risk in adults: A report of the American college of cardiology/American heart association task force on practice guidelines. *J Am Coll Cardiol*. 2014;63(25 PART B):2889-2934. doi:10.1016/j.jacc.2013.11.002
25. Lip GY. The CHA2DS2-VASc Score for Stroke Risk Stratification in Patients with Atrial Fibrillation: A Brief History. *Eur Heart J*. 2015;36:2880-2885.
26. Ledwidge M, Gallagher J, Conlon C, et al. Natriuretic peptide-based screening and

collaborative care for heart failure: The STOP-HF randomized trial. *JAMA - J Am Med Assoc*. 2013;310(1):66-74. doi:10.1001/jama.2013.7588
27. Zhang KW, Finkelman BS, Gulati G, et al. Abnormalities in 3-Dimensional Left Ventricular Mechanics With Anthracycline Chemotherapy Are Associated With Systolic and Diastolic Dysfunction. *JACC Cardiovasc Imaging*. 2018;11(8):1059-1068. doi:10.1016/j.jcmg.2018.01.015
28. Finkelman BS, Putt M, Wang T, et al. Arginine-Nitric Oxide Metabolites and Cardiac Dysfunction in Patients With Breast Cancer. *J Am Coll Cardiol*. 2017;70(2):152-162. doi:10.1016/j.jacc.2017.05.019
29. Narayan HKHK, Finkelman B, French B, et al. Detailed Echocardiographic Phenotyping in Breast Cancer Patients: Associations with Ejection Fraction Decline, Recovery, and Heart Failure Symptoms over 3 Years of Follow-Up. *Circulation*. 2017;135(15):1397-1412. doi:10.1161/CIRCULATIONAHA.116.023463
30. Narayan HK, French B, Khan AM, et al. Noninvasive Measures of Ventricular-Arterial Coupling and Circumferential Strain Predict Cancer Therapeutics–Related Cardiac Dysfunction. *JACC Cardiovasc Imaging*. 2016;9(10):1131-1141. doi:10.1016/j.jcmg.2015.11.024
31. Demissei B, Hubbard RA, Zhang L, et al. Changes in Cardiovascular Biomarkers with Breast Cancer Therapy and Associations with Cardiac Dysfunction. *J Am Heart Assoc*. 2020;9(2).
32. Demissei BG, Finkelman BS, Hubbard RA, et al. Cardiovascular Function Phenotypes in Response to Cardiotoxic Breast Cancer Therapy. *J Am Coll Cardiol*. 2019;73(2):248-249. doi:10.1016/j.jacc.2018.10.057
33. Demissei BG, Finkelman BS, Hubbard RA, et al. Detailed phenotyping reveals distinct trajectories of cardiovascular function and symptoms with exposure to modern breast cancer therapy. *Cancer*. 2019;125(16). doi:10.1002/cncr.32149
34. Beer LA, Kossenkov A V., Liu Q, et al. Baseline Immunoglobulin e Levels as a Marker of Doxorubicin- and Trastuzumab-Associated Cardiac Dysfunction. *Circ Res*. 2016;119(10):1135-1144. doi:10.1161/CIRCRESAHA.116.309004
35. Upshaw JN, Finkelman BS, Hubbard RA, Al E. Detailed Assessment of Changes in Diastolic Function with Contemporary Breast Cancer Therapy. *JACC Cardiovasc Imaging*. 2019;manuscript.
36. Upshaw JN, Finkelman B, Hubbard RA, et al. Detailed Assessment of Changes in Diastolic Function with Contermporary Breast Cancer Therapy. *Manuscr Revis*. Published online 2020.
37. Weiner RB, Baggish AL, Chen-Tournoux A, et al. Improvement in structural and functional echocardiographic parameters during chronic heart failure therapy guided by natriuretic peptides: Mechanistic insights from the ProBNP Outpatient Tailored Chronic Heart Failure (PROTECT) study. *Eur J Heart Fail*. 2013;15(3):342-351. doi:10.1093/eurjhf/hfs180
38. Januzzi JL. The role of natriuretic peptide testing in guiding chronic heart failure management: Review of available data and recommendations for use. *Arch Cardiovasc Dis*. 2012;105(1):40-50. doi:10.1016/j.acvd.2011.10.007
39. Lainchbury JG, Troughton RW, Strangman KM, et al. N-Terminal Pro-B-Type Natriuretic Peptide-Guided Treatment for Chronic Heart Failure. Results From the BATTLESCARRED (NT-proBNP-Assisted Treatment To Lessen Serial Cardiac

Readmissions and Death) Trial. *J Am Coll Cardiol*. 2009;55(1):53-60. doi:10.1016/j.jacc.2009.02.095

40. Stevens PL, Lenihan DJ, Ky B, et al. Prediction and Early Detection of Anthracycline-Related Cardiotoxicity Using Cardiac Biomarkers. *J Clin Oncol*. 2014;32(15_supplement):9644.
41. Chatterjee S, Biondi-Zoccai G, Abbate A, et al. Benefits of blockers in patients with heart failure and reduced ejection fraction: Network meta-analysis. *BMJ*. 2013;346(7893):f55. doi:10.1136/bmj.f55
42. Yancy CW, Januzzi JL, Allen LA, et al. 2017 ACC Expert Consensus Decision Pathway for Optimization of Heart Failure Treatment: Answers to 10 Pivotal Issues About Heart Failure With Reduced Ejection Fraction: A Report of the American College of Cardiology Task Force on Expert Consensus Decision Pathways. *J Am Coll Cardiol*. 2018;71(2):201-230. doi:10.1016/j.jacc.2017.11.025
43. Januzzi JL, Camargo CA, Anwaruddin S, et al. The N-terminal Pro-BNP investigation of dyspnea in the emergency department (PRIDE) study. *Am J Cardiol*. 2005;95(8):948-954. doi:10.1016/j.amjcard.2004.12.032
44. Januzzi JL, Chen-Tournoux AA, Christenson RH, et al. N-Terminal Pro–B-Type Natriuretic Peptide in the Emergency Department: The ICON-RELOADED Study. *J Am Coll Cardiol*. 2018;71(11):1191-1200. doi:10.1016/j.jacc.2018.01.021
45. Fadol A, Mendoza T, Gning I, et al. Psychometric Testing of the MDASI-HF: A Symptom Assessment Instrument for Patients With Cancer and Concurrent Heart Failure. *J Card Fail*. 2008;14(6):497-507. doi:10.1016/j.cardfail.2008.01.012
46. Zhao F, Cella D, Manola J, DiPaola RS, Wagner LI, Haas NSB. Fatigue among patients with renal cell carcinoma receiving adjuvant sunitinib or sorafenib: patient-reported outcomes of ECOG-ACRIN E2805 trial. *Support Care Cancer*. 2018;26(6):1889-1895. doi:10.1007/s00520-017-4027-7
47. Acaster S, Dickerhoof R, DeBusk K, Bernard K, Strauss W, Allen LF. Qualitative and quantitative validation of the FACIT-fatigue scale in iron deficiency anemia. *Health Qual Life Outcomes*. 2015;13(1). doi:10.1186/s12955-015-0257-x
48. Çinar D, Yava A. Validity and reliability of functional assessment of chronic illness treatment-fatigue scale in Turkish patients with type 2 diabetes. *Endocrinol Diabetes y Nutr*. 2018;65(7):409-417. doi:10.1016/j.endinu.2018.01.010
49. Holloway L, Humphrey L, Heron L, et al. Patient-reported outcome measures for systemic lupus erythematosus clinical trials: A review of content validity, face validity and psychometric performance. *Health Qual Life Outcomes*. 2014;12(1). doi:10.1186/s12955-014-0116-1
50. Estabrook R, Cella D, Zhao F, et al. Longitudinal and dynamic measurement invariance of the FACIT-Fatigue scale: an application of the measurement model of derivatives to ECOG-ACRIN study E2805. *Qual Life Res*. 2018;27(6):1589-1597. doi:10.1007/s11136-018-1817-4
51. Salsman JM, Beaumont JL, Wortman K, Yan Y, Friend J, Cella D. Brief versions of the FACIT-fatigue and FAACT subscales for patients with non-small cell lung cancer cachexia. *Support Care Cancer*. 2015;23(5):1355-1364. doi:10.1007/s00520-014-2484-9
52. Garcia SF, Cella D, Clauser SB, et al. Standardizing patient-reported outcomes assessment in cancer clinical trials: A patient-reported outcomes measurement information system initiative. In: *Journal of Clinical Oncology*. Vol 25. J Clin Oncol;

2007:5106-5112. doi:10.1200/JCO.2007.12.2341

53. Hays RD, Bjorner JB, Revicki DA, Spritzer KL, Cella D. Development of physical and mental health summary scores from the patient-reported outcomes measurement information system (PROMIS) global items. *Qual Life Res*. 2009;18(7):873-880. doi:10.1007/s11136-009-9496-9
54. Basch E, Reeve BB, Mitchell SA, et al. Development of the national cancer institute's patient-reported outcomes version of the common terminology criteria for adverse events (PRO-CTCAE). *J Natl Cancer Inst*. 2014;106(9). doi:10.1093/jnci/dju244
55. Basch E, Pugh SL, Dueck AC, et al. Feasibility of Patient Reporting of Symptomatic Adverse Events via the Patient-Reported Outcomes Version of the Common Terminology Criteria for Adverse Events (PRO-CTCAE) in a Chemoradiotherapy Cooperative Group Multicenter Clinical Trial. In: *International Journal of Radiation Oncology Biology Physics*. Vol 98. Elsevier Inc.; 2017:409-418. doi:10.1016/j.ijrobp.2017.02.002
56. Godin G, Shephard RJ. A simple method to assess exercise behavior in the community. *Can J Appl Sport Sci*. 1985;10(3):141-146.
57. Amireault S, Godin G, Lacombe J, Sabiston CM. Validation of the Godin-Shephard Leisure-Time Physical Activity Questionnaire classification coding system using accelerometer assessment among breast cancer survivors. *J Cancer Surviv*. 2015;9(3):532-540. doi:10.1007/s11764-015-0430-6
58. Peipert JD, Badawy SM, Baik SH, et al. Development of the nih patient-reported outcomes measurement information system (Promis) medication adherence scale (pmas). *Patient Prefer Adherence*. 2020;14:971-983. doi:10.2147/PPA.S249079

END OF DOCUMENT
