# Supplementary material for: Biomarker-Guided Cardioprotection for Patients Treated With Anthracyclines: A Randomized Clinical Trial
Source: JAMA Netw Open. 2025 Dec 3;8(12):e2546201. doi: 10.1001/jamanetworkopen.2025.46201 (PMC12676363; doi:10.1001/jamanetworkopen.2025.46201)
Supplement: Supplement 2. — eMethods 1. Biomarker Assessment eMethods 2. Echocardiography Acquisition and Quantification eMethods 3. Questionnaires eMethods 4. Statistical Analyses eTable 1. Eligibility Criteria eTable 2. Distribution of Total Daily Dose (mg) of Neurohormonal Antagonist Therapy for Participants in the NT-proBNP-Guided Arm Across All Study Timepoints eTable 3. Estimated Mean Change from Baseline in Echocardiographic Parameters Over Time Between the Two Arms eTable 4. Estimated Mean Change From Baseline in Echocardiographic Parameters Over Time Between the Two Arms, Adjusted for Baseline Use of Neurohormonal Therapy and Treatment With Pembrolizumab eTable 5. Estimated Mean Change From Baseline in Echocardiographic Parameters Over Time Between the Two Arms eFigure 1. Study Design eFigure 2. Recommended Algorithm for Neurohormonal Therapy in the NT-proBNP-Guided Study Arm eFigure 3. Neurohormonal Therapy Use by Treatment Arm Over Time eFigure 4. Patient-Reported Outcomes for Cardiac Symptoms by Treatment Arm eReferences. [file jamanetwopen-e2546201-s002.pdf]

Supplementary Online Content

Xia C, Smith AM, Lefebvre B, et al. Biomarker-guided cardioprotection for patients treated with anthracyclines: a randomized clinical trial. *JAMA Netw Open.* 2025;8(12):e2546201. doi:10.1001/jamanetworkopen.2025.46201

eMethods 1. Biomarker Assessment
eMethods 2. Echocardiography Acquisition and Quantification
eMethods 3. Questionnaires
eMethods 4. Statistical Analyses
eTable 1. Eligibility Criteria
eTable 2. Distribution of Total Daily Dose (mg) of Neurohormonal Antagonist Therapy for Participants in the NT-proBNP-Guided Arm Across All Study Timepoints
eTable 3. Estimated Mean Change from Baseline in Echocardiographic Parameters Over Time Between the Two Arms
eTable 4. Estimated Mean Change From Baseline in Echocardiographic Parameters Over Time Between the Two Arms, Adjusted for Baseline Use of Neurohormonal Therapy and Treatment With Pembrolizumab
eTable 5. Estimated Mean Change From Baseline in Echocardiographic Parameters Over Time Between the Two Arms
eFigure 1. Study Design
eFigure 2. Recommended Algorithm for Neurohormonal Therapy in the NT-proBNP-Guided Study Arm
eFigure 3. Neurohormonal Therapy Use by Treatment Arm Over Time
eFigure 4. Patient-Reported Outcomes for Cardiac Symptoms by Treatment Arm
eReferences.

This supplementary material has been provided by the authors to give readers additional information about their work.

© 2025 Xia C et al. *JAMA Network Open.*

eMethods 1. Biomarker Assessment

The Elecsys NT-proBNP II assay (Roche Diagnostics 0931568190) on the Cobas e411 platform (Roche Diagnostics) was used for the post-hoc, central quantitation of NT-proBNP concentrations from EDTA plasma samples. This assay has a measurement range of 10 to 35,000 ng/L and a coefficient of variation of 2.9% to 6.1%. All assays were conducted blinded to randomization arm at the Biomarker Discovery and Validation Core of the University of Ottawa Heart Institute.

© 2025 Xia C et al. *JAMA Network Open.*

eMethods 2. Echocardiography Acquisition and Quantification

Transthoracic echocardiograms were performed by dedicated sonographers for all participants according to a standardized protocol. Briefly, images from the apical, parasternal short axis at the mid-papillary level and parasternal long axis views were acquired and digitally archived at 60–80 frames/second. Doppler interrogations of the left ventricular (LV) outflow tract, mitral inflow, and mitral annulus were performed in the apical view. All data were stored as DICOM images for the quantitation of the trial's secondary outcomes, including systolic function (LVEF), structure (LV volumes, mass), diastolic function (E/e'), longitudinal and circumferential strain, and ventricular-arterial coupling (Ea/Ees, ratio of effective arterial elastance [Ea], and end-systolic elastance [Ees]). LV volumes and mass were indexed to body surface area. Measurements were quantified by an experienced, certified sonographer blinded to clinical characteristics and treatment arm on the Tomtec Imaging Systems software platform at the Penn Center for Quantitative Echocardiography, using established recommendations by the American Society of Echocardiography[1] or established protocols. The reproducibility of echocardiographic measures from the core lab has been well established, with an intraobserver coefficient of variation for LVEF at 4.4%.[2]

© 2025 Xia C et al. *JAMA Network Open.*

eMethods 3. Questionnaires

Questionnaires were collected at baseline and 3, 6, 9, and 12 months. Self-reported symptomatic adverse events were recorded according to the patient-reported outcomes version of the Common Terminology Criteria for Adverse Events (PRO-CTCAE).[3] Selected PRO-CTCAEs included shortness of breath, cough, chest pain, arm or leg swelling, palpitations, fatigue, and dizziness. For each symptom, up to three additional items assessed frequency, severity, and interference. Scores for each attribute were combined into a single composite score ranging from 0-3 for each symptom via an established algorithm.[4,5] A higher composite score was indicative of more significant symptoms. To assess medication compliance, the patient-reported outcomes measurement information system (PROMIS) Medication Adherence Scale (PMAS) was used for participants in the NT-proBNP-guided arm when neurohormonal antagonists were prescribed. A higher score on the PMAS indicated greater medication adherence (score range 9 to 45).[6]

© 2025 Xia C et al. *JAMA Network Open.*

eMethods 4. Statistical Analyses

An intention-to-treat approach with all randomized participants analyzed according to randomization arm was used for the primary outcomes analyses. Baseline characteristics were summarized using descriptive statistics according to the two randomized arms. Frequency and percentages were reported for categorical variables; mean (standard deviation) or median (Quartile 1, Quartile 3) were reported for continuous variables. Proportions of participants with grade 3 or higher adverse events by randomization arm were compared using the Chi-square test or Fisher's exact test as appropriate. The PRO-CTCAE was adjusted for baseline scores using the baseline subtraction method.[4] Specifically, for each participant, symptoms recorded during follow-up were considered only if they were worse than the baseline score. The distribution of individual symptoms according to grade was graphically displayed.

A modified intention-to-treat approach, defined as participants with a baseline and at least one follow-up measurement, was used in the secondary outcomes analysis that evaluated the change in NT-proBNP and echocardiographic outcomes in the two arms over time. NT-proBNP values were log2-transformed given their skewed distribution, where each 1-unit increase represents a doubling. Trajectories of NT-proBNP over time were assessed graphically by plotting the estimated marginal mean changes from baseline separately for each arm. Mean changes were estimated using linear regression models via generalized estimating equations (GEE) for repeated measures. Models were adjusted for baseline NT-proBNP concentrations and included natural spline terms for time since anthracycline chemotherapy initiation with three degrees of freedom, accounting for the potential non-linear relationship of time since initiation of cancer therapy and log2 NT-proBNP values, based on previously published work.[7] To evaluate the possible differences in the trajectories over time between the two arms, we introduced a treatment-by-time interaction term. To compare the mean change in each echocardiographic outcome over time across the two arms, GEE models included a factorial treatment-by-visit interaction term, with adjustments for baseline echocardiographic values and an unstructured correlation matrix for the clustering of participants. The Wald test was used to assess statistical significance.

In sensitivity analysis, separate GEE models were fully adjusted for baseline echocardiographic values, baseline use of neurohormonal therapy (yes/no) and treatment with pembrolizumab (yes/no) to account for their potential confounding influences on the treatment effect. Additionally, outcomes between the two arms were explored, restricting the analysis to only those patients with NT-proBNP elevations during the study.

Given that secondary outcomes were *a priori* considered to be exploratory, we did not adjust the significance level for multiple testing. A two-sided p-value of <0.05 was considered statistically significant. All statistical analyses were conducted using R 4.4.0 (R Foundation for Statistical Computing).

© 2025 Xia C et al. *JAMA Network Open.*

eTable 1. Eligibility Criteria

Participants had to meet all the inclusion criteria to be included<br><br>**Inclusion criteria**<br>1. Male or female, ⩾ 18 years of age<br>2. Diagnosed with breast cancer or lymphoma (any subtype), planned to receive an anthracycline-based chemotherapy regimen<br>3. Provision of written informed consent and HIPAA authorization<br>4. Stated willingness to comply with all study procedures and availability for the duration of the study<br>5. Patients may be enrolled up to their first dose of anthracycline even if they have already received other chemotherapeutic or targeted agents as part of neo-adjuvant or adjuvant systemic therapy.
Patients who met any one of the following exclusion criteria were excluded<br><br>**Exclusion criteria**<br>1. Diagnosed with Stage IV breast cancer<br>2. Uncontrolled blood pressure defined by SBP > 180mmHg on two or more occasions and taking three or more antihypertensives within 1 month prior to enrollment.<br>3. Baseline systolic blood pressure < 90mmHg within 1 month prior to enrollment (if multiple blood pressures are available in the medical record within 1 month prior to enrollment, the average SBP will be considered)<br>4. Patient must not have any of the following<br>4-1 Severe hepatic impairment, defined as serum bilirubin > ULN, or AST or ALT > 5.0 ULN on most recent labs prior to enrollment. Results of serum bilirubin, AST, and ALT must be checked for screening if no results available in the EMR within 28 days prior to enrollment.<br>4-2 End-stage renal failure on dialysis<br>4-3 Hyperkalemia with a potassium > 5.5 mEq/L on most recent labs prior to enrollment. Serum potassium must be checked for screening if no results available in the EMR within 28 days prior to enrollment.<br>4-4 A history of kidney transplant<br>4-5 An eGFR < 30 mL/min/1.73m^2 at most recent check prior to enrollment. Creatinine must be checked for screening if no results available in the EMR within 28 days prior to enrollment<br>4-6 Cardiogenic shock<br>4-7 Decompensated heart failure requiring the use of IV inotropic therapy<br>5. Women must not be pregnant or breast-feeding due to the potential harm to an unborn fetus and possible risk for adverse events in nursing infants with some anti-hypertensives, including angiotensin receptor blockers. All females of childbearing potential must have a blood test or urine study within 10 days prior to enrollment to rule out pregnancy. All females of childbearing potential must be strongly advised to use accepted and effective methods of contraception or to abstain from sexual intercourse for the duration of their participation in the study. A female of childbearing potential is defined as any woman, regardless of sexual orientation or whether they have undergone tubal ligation, who meets the following criteria: 1) has achieved menarche at some point, 2) has not undergone a hysterectomy or bilateral oophorectomy; or 3) has not been naturally postmenopausal (amenorrhea following cancer therapy does not rule out childbearing potential) for at least 24 consecutive months (i.e., has had menses at any time in the preceding 24 consecutive months).<br>6. Prior or concurrent malignancy, which in the opinion of the investigator, has the potential to interfere with the safety or efficacy assessment of the investigational regimen<br>7. Non-English speaking

© 2025 Xia C et al. *JAMA Network Open.*

eTable 2. Distribution of Total Daily Dose (mg) of Neurohormonal Antagonist Therapy for Participants in the NT-proBNP-Guided Arm Across All Study Timepoints

	**N**	**Min**	**Q1**	**Median**	**Q3**	**Max**
Carvedilol	9	6.25	6.25	12.5	25	50
Metoprolol	5	50	50	100	125	175
Lisinopril	19	2.5	2.5	6.25	25	40
Losartan	4	25	25	50	100	100
Irbesartan	1	75	75	75	75	75
Valsartan	5	40	40	40	73	320
Spironolactone	2	25	25	25	50	50
Eplerenone	5	12.5	25	25	50	50
Valsartan-hydrochlorothiazide	1	320-25	320-25	320-25	320-25	320-25
Sacubitril-valsartan	2	48-52	48-52	48-52	48-52	48-52

N: Number of participants on medication; Q: Quartile

© 2025 Xia C et al. *JAMA Network Open.*

eTable 3. Estimated Mean Change from Baseline in Echocardiographic Parameters Over Time Between the Two Arms

Parameter	Visits	NT-proBNP-guided	Usual care	Estimated Difference	P value
Left ventricular ejection fraction (%)	3 months	0.9 (-0.4, 2.1)	-1.2 (-2.0, -0.3)	2.0 (0.5, 3.5)	0.007
	6 months	0.9 (-0.2, 2.0)	-0.2 (-1.0, 0.6)	1.1 (-0.3, 2.4)	0.13
	9 months	0.7 (-0.4, 1.8)	0.3 (-0.6, 1.1)	0.4 (-1.0, 1.8)	0.57
	12 months	0.5 (-0.6, 1.5)	0.4 (-0.6, 1.4)	0.0 (-1.4, 1.5)	0.96
LV end systolic volume index to BSA (mL/m^2)	3 months	0.1 (-0.7, 0.9)	1.0 (0.3, 1.7)	-0.9 (-2.0, 0.1)	0.09
	6 months	0.9 (0.0, 1.7)	0.7 (0.1, 1.3)	0.1 (-0.9, 1.2)	0.78
	9 months	1.0 (0.1, 1.9)	0.7 (-0.1, 1.4)	0.3 (-0.8, 1.5)	0.57
	12 months	1.3 (0.5, 2.1)	0.2 (-0.5, 1.0)	1.0 (-0.1, 2.1)	0.07
LV end diastolic volume index to BSA (mL/m^2)	3 months	1.5 (0.0, 3.0)	1.8 (0.5, 3.0)	-0.3 (-2.2, 1.6)	0.78
	6 months	3.3 (1.7, 4.9)	2.0 (1.0, 3.1)	1.3 (-0.6, 3.2)	0.18
	9 months	3.1 (1.8, 4.5)	2.3 (1.1, 3.5)	0.8 (-1.0, 2.6)	0.39
	12 months	3.7 (2.2, 5.3)	1.7 (0.3, 3.0)	2.1 (0.0, 4.1)	0.05
LV mass index to BSA (g/m^2)	3 months	1.2 (-2.0, 4.3)	-1.8 (-5.2, 1.6)	2.9 (-1.7, 7.6)	0.22
	6 months	2.3 (-0.8, 5.3)	-0.1 (-2.7, 2.6)	2.3 (-1.7, 6.4)	0.26
	9 months	0.6 (-2.2, 3.4)	1.0 (-1.6, 3.7)	-0.4 (-4.3, 3.4)	0.82
	12 months	0.7 (-2.4, 3.7)	-3.0 (-5.2, -0.7)	3.6 (-0.2, 7.5)	0.06
E/e’	3 months	0.2 (-0.5, 1.0)	0.9 (0.0, 1.8)	-0.7 (-1.9, 0.5)	0.23
	6 months	0.2 (-0.5, 1.0)	0.6 (-0.2, 1.4)	-0.4 (-1.4, 0.7)	0.5
	9 months	0.1 (-0.6, 0.7)	0.1 (-0.5, 0.6)	0.0 (-0.8, 0.9)	0.99
	12 months	0.9 (-1.0, 2.7)	-0.1 (-0.6, 0.4)	0.9 (-0.9, 2.7)	0.31
Global longitudinal peak strain (%)	3 months	0.0 (-0.6, 0.6)	0.6 (-0.1, 1.2)	-0.5 (-1.4, 0.4)	0.24
	6 months	0.1 (-0.5, 0.8)	0.6 (0.0, 1.3)	-0.5 (-1.4, 0.4)	0.29
	9 months	0.2 (-0.4, 0.8)	0.3 (-0.4, 1.0)	-0.1 (-1.0, 0.8)	0.85
	12 months	0.9 (0.3, 1.4)	0.6 (0.0, 1.2)	0.3 (-0.6, 1.1)	0.54
Global circumferential peak strain (%)	3 months	0.4 (-1.2, 2.0)	1.5 (0.1, 2.9)	-1.0 (-3.3, 1.2)	0.36
	6 months	0.5 (-1.1, 2.1)	1.3 (-0.1, 2.7)	-0.8 (-3.0, 1.4)	0.47
	9 months	1.9 (0.3, 3.4)	1.9 (0.6, 3.1)	-0.0 (-2.1, 2.1)	>0.99
	12 months	0.6 (-0.8, 2.0)	0.8 (-0.5, 2.0)	-0.2 (-2.1, 1.8)	0.87
Ea/Ees	3 months	0.08 (0.02, 0.14)	0.09 (0.03, 0.15)	-0.01 (-0.09, 0.07)	0.77

© 2025 Xia C et al. *JAMA Network Open.*

	6 months	0.11 (0.04, 0.17)	0.08 (0.02, 0.14)	0.03 (-0.06, 0.12)	0.49
	9 months	0.08 (0.03, 0.13)	0.08 (0.03, 0.13)	-0.00 (-0.07, 0.07)	0.97
	12 months	0.07 (0.02, 0.12)	0.08 (0.03, 0.13)	-0.01 (-0.08, 0.07)	0.82

Data are estimated mean (95% confidence interval) and represent the Visit Number – Baseline for the NT-proBNP-guided and Usual care arms. Generalized estimating equations with an unstructured within-subject correlation were used. Models included factorial interactions for treatment and visits and were adjusted for baseline echocardiographic measures. The estimated difference represents the NT-proBNP-guided – Usual care. As an example, the NT-proBNP-guided arm has an estimated 2.0% higher LVEF than Usual care at 3 months. LV = Left ventricular; BSA = body surface area; E/e' = E velocity (cm/s) / average of septal and lateral e' (reflects diastolic function); Ea/Ees = Effective arterial elastance / end-systolic elastance (reflects ventricular-arterial coupling ratio)

© 2025 Xia C et al. *JAMA Network Open.*

eTable 4. Estimated Mean Change From Baseline in Echocardiographic Parameters Over Time Between the Two Arms, Adjusted for Baseline Use of Neurohormonal Therapy and Treatment With Pembrolizumab

Parameter	Visits	NT-proBNP-guided	Usual care	Estimated Difference	P value
Left ventricular ejection fraction (%)	3 months	0.9 (-0.3, 2.1)	-1.1 (-2.0, -0.1)	1.9 (0.4, 3.4)	0.01
	6 months	0.9 (-0.2, 2.0)	-0.1 (-1.0, 0.9)	1.0 (-0.4, 2.3)	0.16
	9 months	0.7 (-0.5, 1.8)	0.4 (-0.6, 1.4)	0.3 (-1.1, 1.7)	0.68
	12 months	0.4 (-0.6, 1.5)	0.5 (-0.6, 1.6)	-0.1 (-1.5, 1.4)	0.91
LV end systolic volume index to BSA (mL/m^2)	3 months	0.1 (-0.7, 1.0)	1.0 (0.3, 1.7)	-0.9 (-1.9, 0.2)	0.11
	6 months	0.9 (0.0, 1.8)	0.7 (0.0, 1.4)	0.2 (-0.8, 1.2)	0.69
	9 months	1.0 (0.1, 2.0)	0.6 (-0.2, 1.5)	0.4 (-0.7, 1.5)	0.49
	12 months	1.3 (0.5, 2.1)	0.2 (-0.6, 1.0)	1.1 (-0.0, 2.2)	0.05
LV end diastolic volume index to BSA (mL/m^2)	3 months	1.9 (0.3, 3.5)	2.2 (0.9, 3.5)	-0.3 (-2.1, 1.6)	0.78
	6 months	3.7 (2.1, 5.4)	2.4 (1.3, 3.6)	1.3 (-0.6, 3.1)	0.17
	9 months	3.5 (2.1, 5.0)	2.8 (1.4, 4.1)	0.8 (-1.0, 2.5)	0.39
	12 months	4.1 (2.6, 5.7)	2.1 (0.5, 3.7)	2.0 (0.0, 4.0)	0.05
LV mass index to BSA (g/m^2)	3 months	1.0 (-2.1, 4.1)	-1.5 (-5.3, 2.3)	2.6 (-2.1, 7.2)	0.28
	6 months	2.3 (-0.6, 5.2)	0.2 (-2.5, 2.9)	2.1 (-2.0, 6.2)	0.31
	9 months	0.6 (-2.1, 3.3)	1.4 (-1.4, 4.1)	-0.7 (-4.6, 3.1)	0.7
	12 months	0.6 (-2.2, 3.5)	-2.7 (-5.2, -0.1)	3.3 (-0.6, 7.2)	0.1
E/e’	3 months	0.5 (-0.3, 1.2)	1.3 (0.4, 2.2)	-0.9 (-2.0, 0.3)	0.14
	6 months	0.4 (-0.4, 1.2)	1.1 (0.2, 1.9)	-0.6 (-1.7, 0.5)	0.27
	9 months	0.3 (-0.3, 0.9)	0.5 (-0.0, 1.1)	-0.3 (-1.1, 0.6)	0.55
	12 months	1.0 (-0.7, 2.8)	0.4 (-0.2, 0.9)	0.7 (-1.1, 2.4)	0.46
Global longitudinal peak strain (%)	3 months	0.1 (-0.5, 0.8)	0.5 (-0.2, 1.2)	-0.4 (-1.2, 0.5)	0.43
	6 months	0.2 (-0.5, 0.9)	0.6 (-0.0, 1.3)	-0.4 (-1.3, 0.5)	0.4
	9 months	0.3 (-0.4, 0.9)	0.3 (-0.4, 1.0)	-0.0 (-1.0, 0.9)	0.96
	12 months	1.0 (0.4, 1.5)	0.7 (0.0, 1.3)	0.3 (-0.5, 1.1)	0.48
Global circumferential peak strain (%)	3 months	-0.1 (-1.6, 1.5)	0.9 (-0.6, 2.3)	-0.9 (-3.0, 1.2)	0.39
	6 months	0.0 (-1.6, 1.6)	0.6 (-0.9, 2.1)	-0.6 (-2.7, 1.5)	0.58
	9 months	1.4 (-0.3, 3.1)	1.2 (-0.1, 2.5)	0.2 (-1.8, 2.1)	0.88
	12 months	0.1 (-1.4, 1.6)	0.0 (-1.4, 1.4)	0.1 (-1.8, 2.0)	0.93

© 2025 Xia C et al. *JAMA Network Open.*

Ea/Ees	3 months	0.09 (0.03, 0.15)	0.10 (0.05, 0.16)	-0.02 (-0.09, 0.06)	0.66
	6 months	0.12 (0.05, 0.19)	0.09 (0.03, 0.15)	0.03 (-0.05, 0.11)	0.49
	9 months	0.09 (0.03, 0.14)	0.09 (0.03, 0.14)	-0.00 (-0.07, 0.07)	0.99
	12 months	0.08 (0.03, 0.13)	0.09 (0.03, 0.15)	-0.01 (-0.08, 0.06)	0.8

Data are estimated mean (95% confidence interval) and represent the Visit Number – Baseline for the NT-proBNP-guided and Usual care. Generalized estimating equations with an unstructured within-subject correlation were used. Models included factorial interactions for treatment and visits and were adjusted for baseline echocardiographic measures, baseline use of neurohormonal therapy and treatment with pembrolizumab. The estimated difference represents the NT-proBNP-guided – Usual care. LV = Left ventricular; BSA = body surface area; E/e’ = E velocity (cm/s) / average of septal and lateral e’ (reflects diastolic function); Ea/Ees = Effective arterial elastance / end-systolic elastance (reflects ventricular-arterial coupling ratio)

© 2025 Xia C et al. *JAMA Network Open.*

eTable 5. Estimated Mean Change From Baseline in Echocardiographic Parameters Over Time Between the Two Arms

Analyses were restricted to participants with NT-proBNP elevations in each arm

Parameter	Visits	NT-proBNP-guided	Usual care	Estimated Difference	P value
Left ventricular ejection fraction (%)	3 months	0.6 (-1.2, 2.4)	-1.6 (-2.5, -0.8)	2.2 (0.3, 4.2)	0.03
	6 months	0.3 (-1.0, 1.5)	-0.7 (-1.8, 0.5)	0.9 (-0.8, 2.7)	0.28
	9 months	0.1 (-1.4, 1.6)	-0.7 (-2.0, 0.5)	0.8 (-1.1, 2.8)	0.39
	12 months	-0.2 (-1.5, 1.1)	0.2 (-1.1, 1.4)	-0.3 (-2.1, 1.4)	0.7
LV end systolic volume index to BSA (mL/m^2)	3 months	0.7 (-0.5, 1.9)	1.4 (0.6, 2.3)	-0.7 (-2.3, 0.8)	0.33
	6 months	1.9 (0.8, 3.0)	1.2 (0.3, 2.0)	0.8 (-0.6, 2.2)	0.28
	9 months	2.0 (0.7, 3.3)	1.5 (0.3, 2.6)	0.5 (-1.2, 2.3)	0.54
	12 months	2.2 (1.1, 3.2)	0.9 (-0.2, 2.0)	1.3 (-0.2, 2.8)	0.09
LV end diastolic volume index to BSA (mL/m^2)	3 months	2.4 (-0.1, 4.8)	2.2 (0.4, 4.0)	0.2 (-2.8, 3.2)	0.89
	6 months	5.0 (2.6, 7.4)	2.5 (1.1, 4.0)	2.5 (-0.3, 5.3)	0.08
	9 months	4.5 (2.6, 6.5)	3.0 (1.2, 4.8)	1.5 (-1.1, 4.1)	0.24
	12 months	4.9 (2.6, 7.1)	2.7 (0.9, 4.5)	2.2 (-0.7, 5.0)	0.13
LV mass index to BSA (g/m^2)	3 months	4.1 (0.8, 7.4)	-0.7 (-4.5, 3.1)	4.8 (-0.3, 9.8)	0.06
	6 months	5.3 (1.4, 9.2)	-0.7 (-3.8, 2.5)	6.0 (0.9, 11.0)	0.02
	9 months	3.0 (0.0, 6.0)	3.1 (-0.2, 6.4)	-0.1 (-4.5, 4.4)	0.98
	12 months	3.2 (-0.3, 6.8)	-2.2 (-4.7, 0.3)	5.5 (1.1, 9.8)	0.01
E/e’	3 months	0.2 (-1.0, 1.5)	0.6 (-0.3, 1.5)	-0.4 (-2.0, 1.2)	0.62
	6 months	0.5 (-0.7, 1.7)	0.7 (-0.5, 1.8)	-0.2 (-1.8, 1.5)	0.85
	9 months	0.1 (-0.9, 1.1)	0.3 (-0.6, 1.2)	-0.2 (-1.5, 1.2)	0.77
	12 months	1.9 (-0.9, 4.7)	-0.1 (-0.8, 0.6)	2.0 (-0.8, 4.8)	0.15
Global longitudinal peak strain (%)	3 months	-0.0 (-1.0, 0.9)	0.5 (-0.4, 1.3)	-0.5 (-1.8, 0.8)	0.43
	6 months	-0.1 (-1.1, 0.8)	0.0 (-0.9, 0.9)	-0.2 (-1.5, 1.2)	0.81
	9 months	0.0 (-0.8, 0.8)	-0.0 (-1.0, 0.9)	0.1 (-1.2, 1.3)	0.93
	12 months	0.5 (-0.1, 1.1)	0.7 (-0.0, 1.5)	-0.2 (-1.2, 0.7)	0.63
Global circumferential peak strain (%)	3 months	1.3 (-0.9, 3.4)	2.1 (0.5, 3.8)	-0.9 (-3.7, 2.0)	0.56
	6 months	0.2 (-2.0, 2.3)	1.4 (-0.3, 3.1)	-1.2 (-4.0, 1.6)	0.4
	9 months	3.0 (1.0, 4.9)	2.3 (0.6, 4.0)	0.7 (-2.0, 3.3)	0.62
	12 months	1.3 (-0.8, 3.3)	2.1 (0.9, 3.4)	-0.9 (-3.4, 1.6)	0.49

© 2025 Xia C et al. *JAMA Network Open.*

Ea/Ees	3 months	0.08 (0.00, 0.15)	0.08 (0.01, 0.16)	-0.01 (-0.11, 0.10)	0.92
	6 months	0.09 (0.01, 0.18)	0.06 (-0.02, 0.14)	0.03 (-0.08, 0.15)	0.58
	9 months	0.10 (0.03, 0.17)	0.07 (0.01, 0.13)	0.03 (-0.06, 0.13)	0.5
	12 months	0.03 (-0.03, 0.10)	0.08 (0.02, 0.14)	-0.05 (-0.14, 0.04)	0.27

Analyses were restricted to participants with NT-proBNP elevations in each arm. Data are estimated mean (95% confidence interval) and represent the Visit Number – Baseline for the NT-proBNP-guided and Usual care. Generalized estimating equations with an unstructured within-subject correlation were used. Models included factorial interactions for treatment and visits and were adjusted for baseline echocardiographic measures. The estimated difference represents the NT-proBNP-guided – Usual care. LV = Left ventricular; BSA = body surface area; E/e' = E velocity (cm/s) / average of septal and lateral e' (reflects diastolic function); Ea/Ees = Effective arterial elastance / end-systolic elastance (reflects ventricular-arterial coupling ratio)

© 2025 Xia C et al. *JAMA Network Open.*

eFigure 1. Study Design

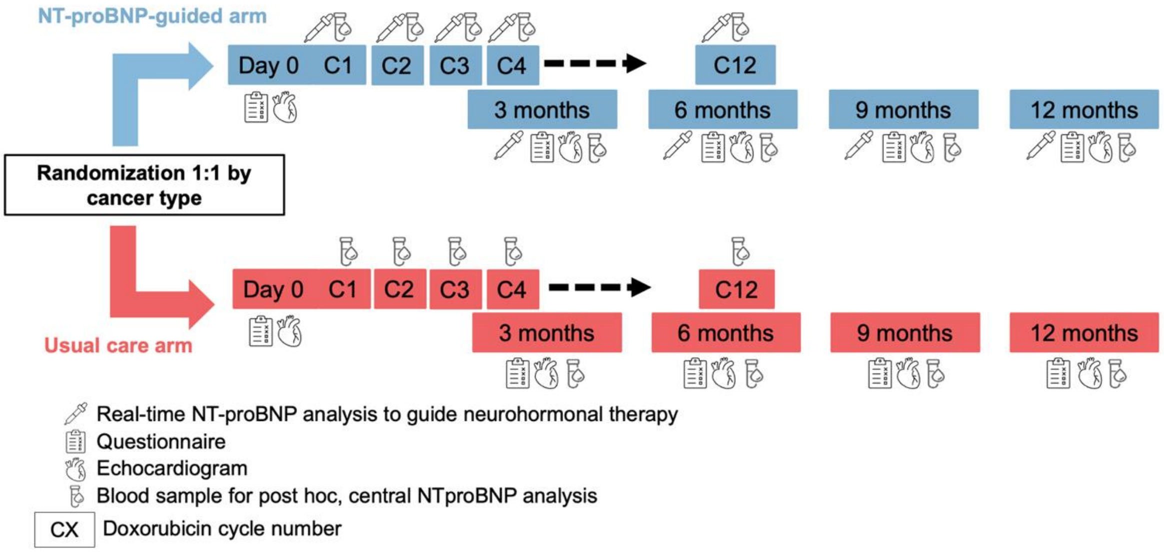

If an anthracycline cycle occurred in the window for the scheduled 3 or 6-month visit, it was combined with that visit and blood was collected only once.

© 2025 Xia C et al. *JAMA Network Open.*

eFigure 2. Recommended Algorithm for Neurohormonal Therapy in the NT-proBNP-Guided Study Arm

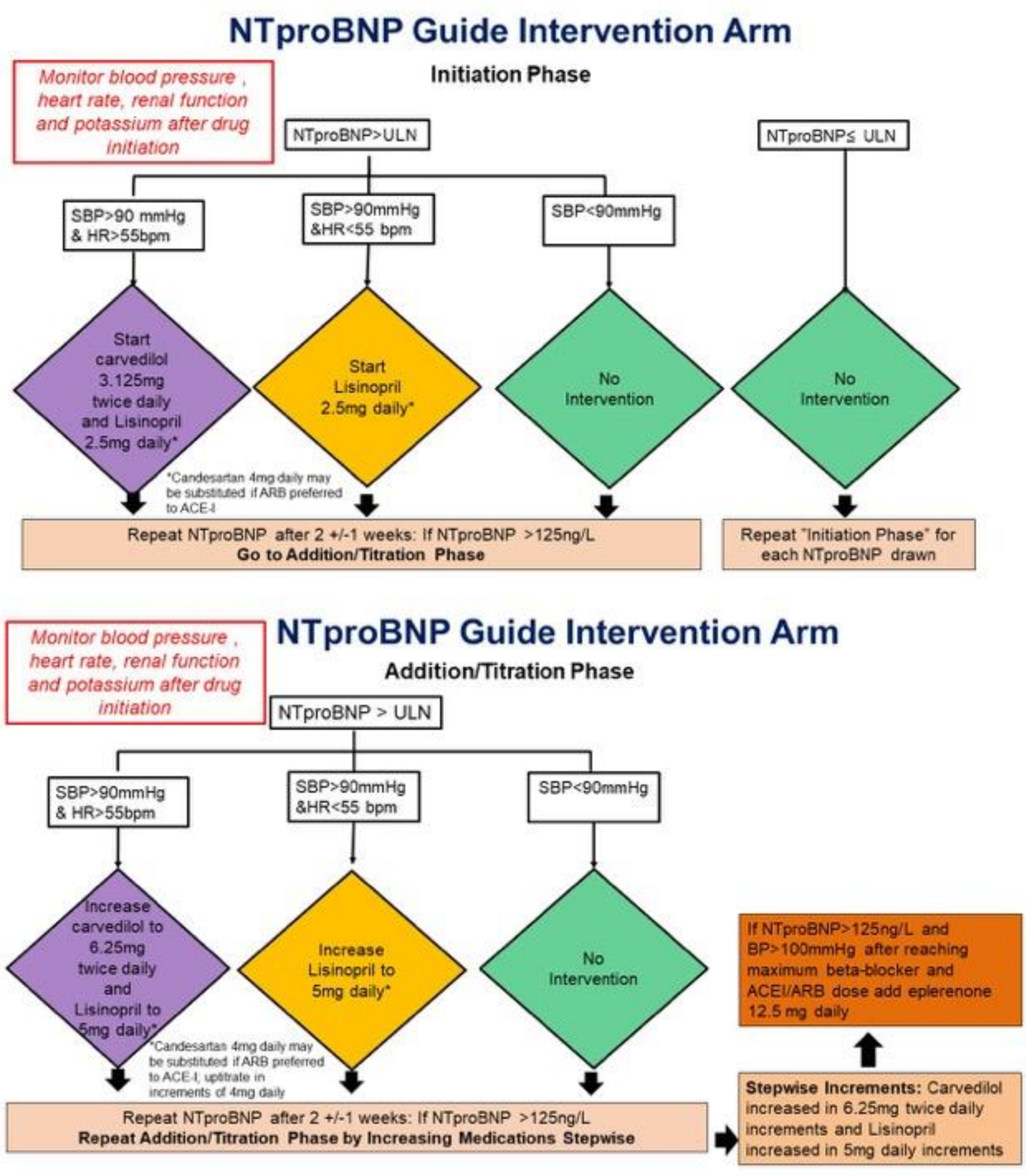

© 2025 Xia C et al. *JAMA Network Open.*

eFigure 3. Neurohormonal Therapy Use by Treatment Arm Over Time

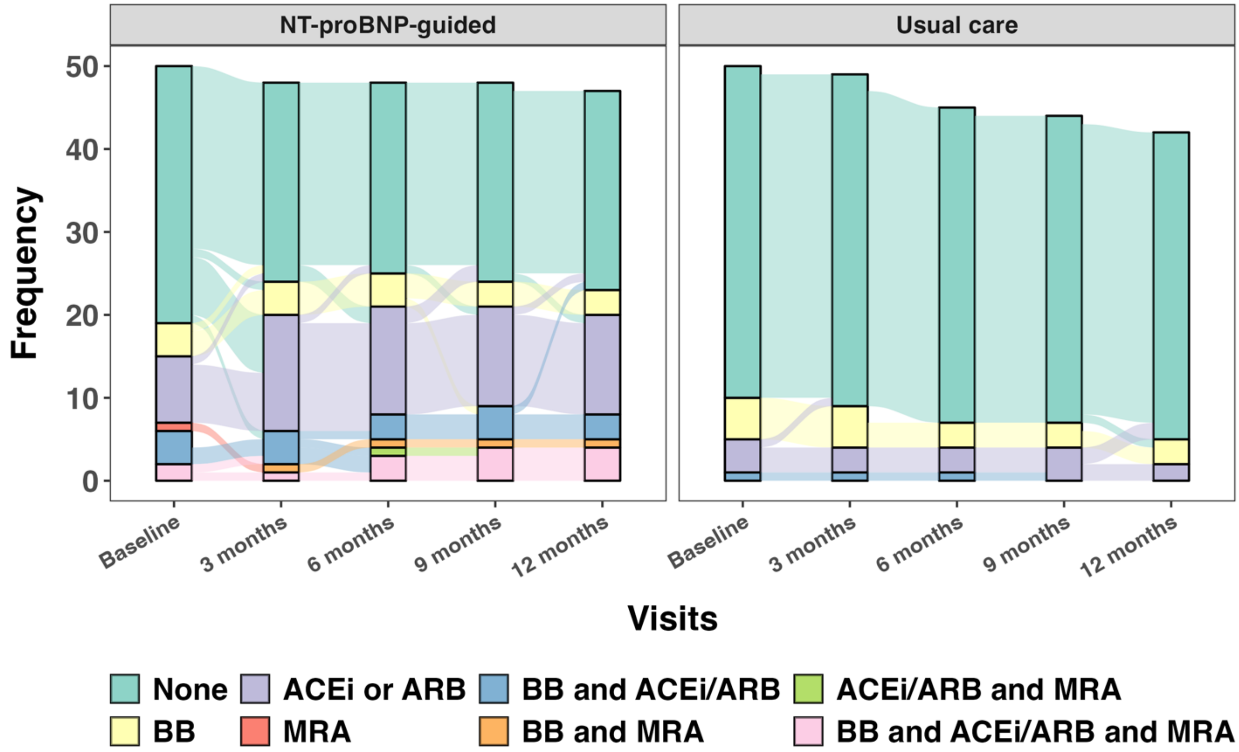

Changes in the frequency of different neurohormonal therapies used in the NT-proBNP-guided arm and usual care at baseline, 3, 6, 9, and 12 months. ACEi: angiotensin-converting enzyme inhibitors; ARB: angiotensin receptor blockers; BB: beta-blockers; MRA: mineralocorticoid receptor antagonist

© 2025 Xia C et al. *JAMA Network Open.*

eFigure 4. Patient-Reported Outcomes for Cardiac Symptoms by Treatment Arm

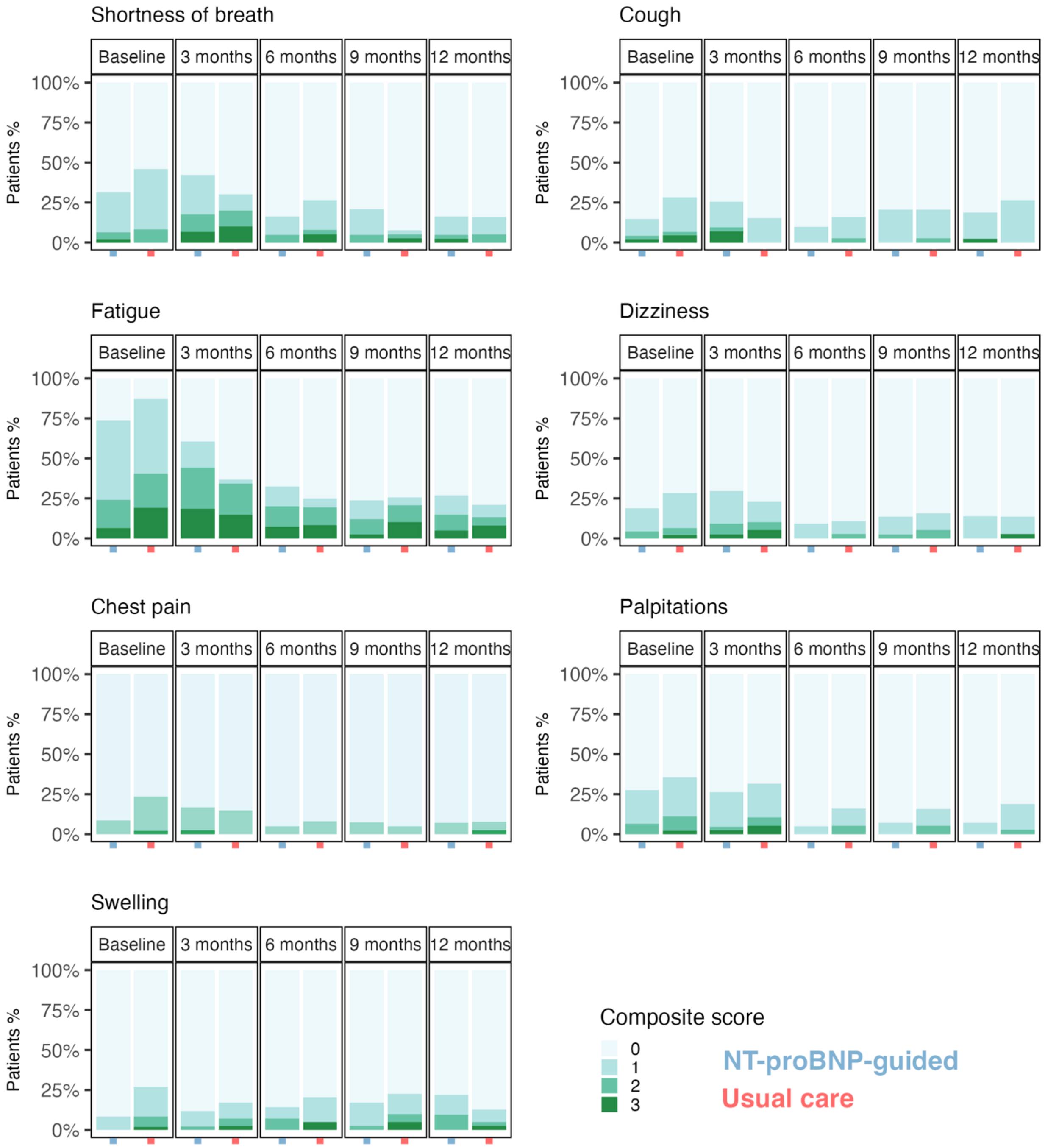

Patient-Reported Outcomes version of the Common Terminology Criteria for Adverse Events (PRO-CTCAE) composite score distributions for each selected symptom (shortness of breath, cough, fatigue, dizziness, chest pain, palpitations, and swelling) in the NT-proBNP-guided arm (blue) and usual care (red), at baseline and at 3, 6, 9, and 12 months, adjusted for baseline symptoms.[4] Higher composite scores (darker green shades) indicate worse symptoms. The lightest green shade denotes a score of zero.

© 2025 Xia C et al. *JAMA Network Open.*

eReferences.

1. Lang RM, Badano LP, Mor-Avi V, et al. Recommendations for cardiac chamber quantification by echocardiography in adults: an update from the American Society of Echocardiography and the European Association of Cardiovascular Imaging. *J Am Soc Echocardiogr*. Jan 2015;28(1):1–39.e14. doi:10.1016/j.echo.2014.10.003
2. Narayan HK, Finkelman B, French B, et al. Detailed Echocardiographic Phenotyping in Breast Cancer Patients: Associations With Ejection Fraction Decline, Recovery, and Heart Failure Symptoms Over 3 Years of Follow-Up. *Circulation*. Apr 11 2017;135(15):1397–1412. doi:10.1161/circulationaha.116.023463
3. Basch E, Reeve BB, Mitchell SA, et al. Development of the National Cancer Institute's patient-reported outcomes version of the common terminology criteria for adverse events (PRO-CTCAE). *J Natl Cancer Inst*. Sep 2014;106(9)doi:10.1093/jnci/dju244
4. Basch E, Rogak LJ, Dueck AC. Methods for Implementing and Reporting Patient-reported Outcome (PRO) Measures of Symptomatic Adverse Events in Cancer Clinical Trials. *Clin Ther*. Apr 2016;38(4):821–30. doi:10.1016/j.clinthera.2016.03.011
5. Basch E, Becker C, Rogak LJ, et al. Composite grading algorithm for the National Cancer Institute's Patient-Reported Outcomes version of the Common Terminology Criteria for Adverse Events (PRO-CTCAE). *Clin Trials*. Feb 2021;18(1):104–114. doi:10.1177/1740774520975120
6. Peipert JD, Badawy SM, Baik SH, et al. Development of the NIH Patient-Reported Outcomes Measurement Information System (PROMIS) Medication Adherence Scale (PMAS). *Patient Prefer Adherence*. 2020;14:971–983. doi:10.2147/ppa.S249079
7. Demissei BG, Hubbard RA, Zhang L, et al. Changes in Cardiovascular Biomarkers With Breast Cancer Therapy and Associations With Cardiac Dysfunction. *J Am Heart Assoc*. Jan 21 2020;9(2):e014708. doi:10.1161/jaha.119.014708

© 2025 Xia C et al. *JAMA Network Open.*
