# Supplementary material for: Biomarker-Guided Cardioprotection for Patients Treated With Anthracyclines: A Randomized Clinical Trial
Source: JAMA Netw Open. 2025 Dec 3;8(12):e2546201. doi: 10.1001/jamanetworkopen.2025.46201 (PMC12676363; doi:10.1001/jamanetworkopen.2025.46201)
Supplement: Supplement 3. — Data Sharing Statement [file jamanetwopen-e2546201-s003.pdf]

Data Sharing Statement

Xia. Biomarker-Guided Cardioprotection for Patients Treated With Anthracyclines. *JAMA Netw Open*. Published December 03, 2025. doi:10.1001/jamanetworkopen.2025.46201

Data
Additional Information: NCT04737265
Data available: No
